# Explicit and Implicit Own's Body and Space Perception in Painful Musculoskeletal Disorders and Rheumatic Diseases: A Systematic Scoping Review

**DOI:** 10.3389/fnhum.2020.00083

**Published:** 2020-04-09

**Authors:** Antonello Viceconti, Eleonora Maria Camerone, Deborah Luzzi, Debora Pentassuglia, Matteo Pardini, Diego Ristori, Giacomo Rossettini, Alberto Gallace, Matthew R. Longo, Marco Testa

**Affiliations:** ^1^Department of Neuroscience, Rehabilitation, Ophthalmology, Genetics, Maternal and Child Health, University of Genova, Savona, Italy; ^2^Department of Neuroscience, Rehabilitation, Ophthalmology, Genetics, Maternal and Child Health, University of Genova, Genoa, Italy; ^3^Policlinico S. Martino IRCCS, Genova, Italy; ^4^Neuromi, Università di Milano-Bicocca, Milan, Italy; ^5^Mind and Behavior Technological Center- Mibtec, Università di Milano-Bicocca, Milan, Italy; ^6^Department of Psychological Sciences, Birkbeck, University of London, London, United Kingdom

**Keywords:** musculoskeletal disorders, rheumatic diseases, chronic pain, somatoperception, body perception, body representation, body ownership, scoping review

## Abstract

**Background:** Pain and body perception are essentially two subjective mutually influencing experiences. However, in the field of musculoskeletal disorders and rheumatic diseases we lack of a comprehensive knowledge about the relationship between body perception dysfunctions and pain or disability. We systematically mapped the literature published about the topics of: (a) somatoperception; (b) body ownership; and (c) perception of space, analysing the relationship with pain and disability. The results were organized around the two main topics of the assessment and treatment of perceptual dysfunctions.

**Methods:** This scoping review followed the six-stage methodology suggested by Arksey and O'Malley. Ten electronic databases and grey literature were systematically searched. The PRISMA Extension for Scoping Reviews was used for reporting results. Two reviewers with different background, independently performed study screening and selection, and one author performed data extraction, that was checked by a second reviewer.

**Results:** Thirty-seven studies fulfilled the eligibility criteria. The majority of studies (68%) concerned the assessment methodology, and the remaining 32% investigated the effects of therapeutic interventions. Research designs, methodologies adopted, and settings varied considerably across studies. Evidence of distorted body experience were found mainly for explicit somatoperception, especially in studies adopting self-administered questionnaire and subjective measures, highlighting in some cases the presence of sub-groups with different perceptual features. Almost half of the intervention studies (42%) provided therapeutic approaches combining more than one perceptual task, or sensory-motor tasks together with perceptual strategies, thus it was difficult to estimate the relative effectiveness of each single therapeutic component.

**Conclusions:** To our knowledge, this is the first attempt to systematically map and summarize this research area in the field of musculoskeletal disorders and rheumatic diseases. Although methodological limitations limit the validity of the evidence obtained, some strategies of assessment tested and therapeutic strategies proposed represent useful starting points for future research. This review highlights preliminary evidence, strengths, and limitations of the literature published about the research questions, identifying key points that remain opened to be addressed, and make suggestions for future research studies. Body representation, as well as pain perception and treatment, can be better understood if an enlarged perspective including body and space perception is considered.

## Introduction

The body is a unique multisensory object (Longo et al., 2008a) integrating a large variety of inputs both from the outside and from within the body (Gallace and Spence, 2008), thus offering the opportunity for a better interaction with the complex surrounding world (Medina and Coslett, 2016a). We can experience our own body through the basic somatic sensations of touch, warmth, cold, proprioception, nociception and itch coming from peripheral receptors to central specific cortical areas (*somatosensation)*. However, our body interaction with the surrounding world is also made by more rich and complex experiences, as the estimation of body size and shape, or the perception of body parts localization in external space *(somatoperception)* (Taylor-Clarke et al., 2004; Longo and Haggard, 2010) for which there are no specialized sensory receptors. The achievement of this more sophisticated perceptual experience requires moving beyond pure *somatosensation* to a higher-order level of neural machinery in which a combination of somatic information converges in associative areas (Murata and Ishida, 2007; Murata et al., 2016) to produce a multimodal representation of the body as a whole (the so called body matrix) (Moseley et al., 2012). This “on line” organization of somatic information is checked for congruence against internal body models (*somatorepresentations)* (Schwoebel and Coslett, 2005; de Vignemont, 2007; Carruthers, 2008; Tsakiris and Fotopoulou, 2008; Berlucchi and Aglioti, 2010; Longo, 2015; Medina and Coslett, 2016a): if the “on line” representation does not match (Azañón and Haggard, 2009) the “off line” body memory (Riva, 2018) we experience a body incoherence, from which misperceptions and bodily illusions may arise.

In addition, as a part of our body interaction with the surrounding world, how we experience our own's body relates also to our sense of self, understood as the perceptual feeling that a body part belong to us (*ownership*), and is under our own control (*agency*) (Tsakiris et al., 2006; Longo et al., 2008a). Internal mental representation of the body includes the shape and contours of own body, the perceived location of body parts, and the boundaries between them and external objects. Body ownership and body agency can be tested experimentally through the Rubber Hand Illusion (RHI) paradigm (Botvinick and Cohen, 1998), in which tactile stimuli are applied synchronously over a prosthetic hand placed in front of the participant, and on his actual hand hidden from view. This produces an illusory sense of incorporation of the rubber hand as it was the participant's own hand (Botvinick, 2004). Overall, how we experience our body and space around us results from the integration of at least three different sub-functions: (a) the perception we have of our own body (*somatoperception*—*SoP*); (b) the perception of the space around us in which we are immersed (space perception—SpP); and (c) the integration of the two body experiences in order to produce a coherent sense of self (*body ownership*—*BO*). Up to now we have only a partial knowledge of the operational mechanisms guiding *SoP* because a large number of studies conducted in the fields of experimental psychology and neurophysiology have mainly studied the basic mechanisms of *somatosensations* while we know much less about the higher-order mechanisms involved in SoP (Longo et al., 2010). Moreover, the research lines have increased the interest on *BO* and SpP only in the last one or two decades (Ramakonar et al., 2011; Trojan et al., 2014).

Musculoskeletal Disorders and Rheumatic Diseases (MDRDs) are a group of diseases commonly affecting bones, muscles and joints (van der Heijde et al., 2018) that often cause chronic pain with a severe impact on the quality of life of patients (March et al., 2014; Blyth et al., 2019), loss of work productivity (Daneshmandi et al., 2017), and significant economic costs for the community (Bevan, 2015; Vos et al., 2017; Briggs et al., 2018). Notably, pain and body perception are essentially two subjective mutually influencing perceptual experiences (Haggard et al., 2013; Trojan et al., 2014): the fast and accurate perception of pain is essential to protect the body, and the perception of body integrity is needed to avoid pain (Wand et al., 2016). Thus, the study of errors in processing the *explicit* (conscious) and *implicit* (unconscious) body experience, as in the case of illusion phenomena (Medina and Coslett, 2016b), may represent a useful opportunity to understand how the brain constructs functional representations of the body in patients with MDRDs, and on pain perception itself (Pamment and Aspell, 2017; Fang et al., 2019) in these clinical conditions. However, existing studies on *SoP*, SpP, and *BO* were largely conducted on healthy subjects (Longo et al., 2008a; Fuentes et al., 2013; Longo, 2017), and clinical research has mostly investigated neurological conditions (Haggard and Wolpert, 2005; Pia et al., 2013, 2016), eating disorders (Keizer et al., 2011; Scarpina et al., 2014; Spitoni et al., 2015; Gadsby, 2017), and neuropathic pain syndromes such as Complex Regional Pain Syndrome-CRPS (Galer and Jensen, 1999; Förderreuther et al., 2004; Lewis et al., 2007; Reinersmann et al., 2013). A large body of literature on the field of MDRDs has instead investigated primary somatosensations (Tsay et al., 2015), mainly tactile acuity (Catley et al., 2013, 2014; Harvie et al., 2018) and proprioceptive precision (Stanton et al., 2016; Tong et al., 2017; Lin et al., 2019), referring generically to disturbances at the level of perception or mental representations. However, both two-point discrimination and joint repositioning error (two of the most frequently investigated tasks) cannot be considered as having a higher-order somatoperceptual involvement (Longo and Haggard, 2010; Hillier et al., 2015; Spitoni et al., 2015). The area of MDRDs thus lacks a comprehensive knowledge about the more complex implicit and explicit body and space perception.

Evidence supporting the interaction between pain and the three mentioned domains of body experience (SoP, SpP, BO) have been found in experimentally-induced pain (Moseley et al., 2008; Gallace et al., 2011; Mancini et al., 2011; Fang et al., 2019) (e.g., distorting the visual appearance of the body). Moreover, a correlation between body and space perception dysfunctions with pain intensity and its duration (Förderreuther et al., 2004; Peltz et al., 2011; Reinersmann et al., 2012), were found in CRPS, thus it would be clinically relevant to clarify if this interaction exists also in MDRDs.

In order to have a comprehensive and structured knowledge of how body experience has been investigated in MDRDs, we systematically reviewed the literature published about the implicit and explicit mechanisms of: (a) *somatoperception* (and indirectly on *somatorepresentations*); (b) *body ownership*; and (c) *space perception*.

The primary goal of this study was to map and examine the quantity and the nature of the scientific literature concerning the implicit and explicit own's body and space perception, organizing the findings around three main topics:

- the adopted strategies of assessment for perceptual dysfunctions;
- the impact of perceptual disorders in MDRDs compared to other disorders (e.g., CRPS) and in sub-groups of MDRDs;
- the interventions proposed to approach perceptual disorders associated to MDRDs.

## Materials and Methods

The scoping methodology has been adopted because represents the most appropriate method to overview the literature about an emergent research area that is still fragmented, complex, wide, poorly understood or not deeply investigated before (Colquhoun et al., 2014, 2017). The review followed the PRISMA Extension for Scoping Reviews (PRISMA-ScR) (Tricco et al., 2018). A detailed PRISMA-ScR is provided Additional File 1. Every deviation from the published protocol (Viceconti et al., 2018) or added procedure were declared. Neuroscientists (ML, AG, MP) and physiotherapists (AV, DL, DP, DR, GR, MT) constituted an inter-professional and interdisciplinary research team with both clinical and scientific background to better approach, from a rehabilitative perspective, a research area that has been historically treated by neuropsychological disciplines (Head and Holmes, 1911; Haggard and Wolpert, 2005; Medina and Coslett, 2016b). In agreement with the concept of a “literature map,” the results are graphically presented in **Figures 3**–**8**.

### Eligibility Criteria

**Figure 1:**
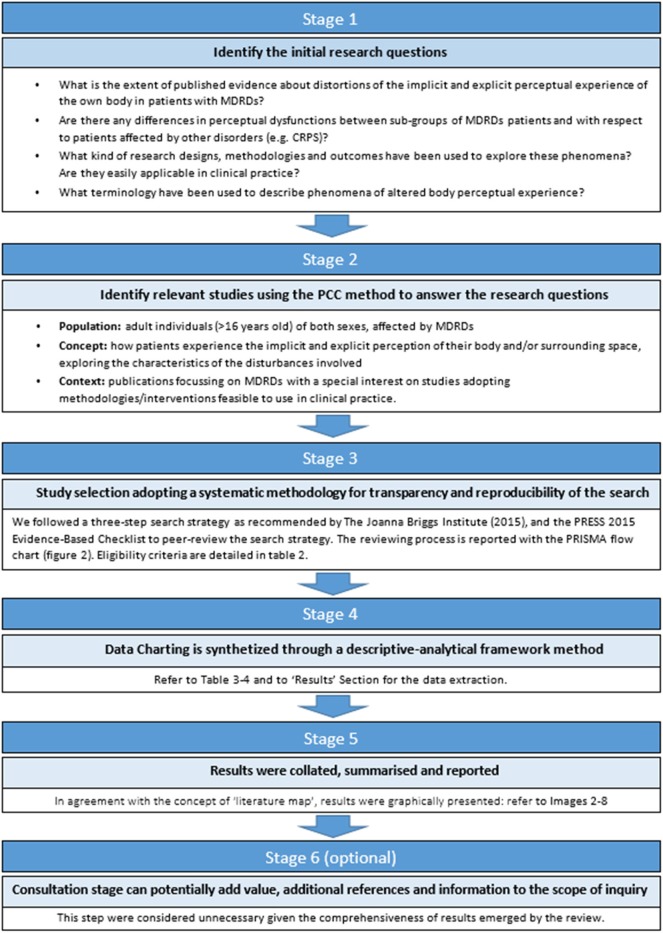
Summary of the adopted guidance framework for scoping reviews.

Inclusion and exclusion criteria are reported in **Table 2**. An iterative process, rather than a fixed and pre-established searching schema, is one of the features characterizing scoping reviews: eligibility criteria were updated in progress by an iterative process based on feedback provided by authors, in order to better refine the searching process according to the research questions (see the step 1 of the Figure 1).

We adopted *a priori* operational definitions of the key terms used (see Table 1 for terminological aspects) in order to avoid terminological misunderstanding. Themes like those dealing with *somatosensation, somatosensory representation*, body scheme, body structural description, body concept, and body affect were not considered as the main focus of this review. In particular, s*ensory representations* and *somatosensations* were often used in literature as a surrogate for *perceptual representations* and *somatoperception*, mainly to describe their associated dysfunctions. Readers are invited to see Flor et al. (1997), Flor (2003), and Hotz-Boendermaker et al. (2016) for maladaptive reorganizations of *somatosensory representations*, and Tsay et al. (2015) for a comprehensive review on *somatosensations*. Moreover, we have deliberately avoided the use of the umbrella term “body image” given the controversies and interpretational difficulties with this term (de Vignemont, 2010; Pitron et al., 2018; Gadsby, 2019): it has often been used as a “*passepartout*” term, lumping together phenomena and psychological capacities quite different from each other, often referred to beliefs and affective attitudes related to the body (Mohr et al., 2010). We have to consider that words used in literature to describe body perception, mental representations and the relative assigned meanings are sometimes ambiguous or contradictory (Gallagher, 1986) and often depend on the observer's professional background., Thus, to better organize the results emerged from the review we have referred to the theoretical model proposed by Longo et al. (2010), Longo (2016) and adapted it for the purposes of this study.

**Table 1:** Terminological definitions.

**Mechanisms**	**Meanings**	**Tasks (examples)**	**Neural bases**
**Somatosensations**	“*How owns body is felt to be like?”* They are the basics mechanisms producing the sensations of touch, proprioception, cold, warm, nociception, vision, etc., for which we own specific receptors, encoding the input information according to the particular type of stimulus processed.	Tactile stimuli detection, precision of touch (e.g., two-point discrimination threshold), repositioning accuracy (e.g., joint position sense).	***Sensorial Representations:*** Primary Somatosensory cortices.
**Somatoperceptions**	“*How owns body is perceived to be like?”* It is referred to the complex perceptual tasks, for which we not own specialized receptors.	Perception of body parts' size and location, the skin localization of tactile stimuli, tactile object recognition, spatial localisation of touch.	***Perceptual representations:*** Superficial schema, postural schema and body model. Parietal cortices, especially in right hemisphere.
**Somatorepresentations** • *Somatosensory Representation* • *Perceptual Representation* • *Cognitive Representation*	With this “umbrella term” it can be grouped a variety of functional and neural configurations about different body characteristics (e.g., sensorial, perceptual or cognitive). In this sense, the conceptual term “*representation”* can assume a variety of meanings on the base of what features are specifically analysed.		
**Cognition**	“*How owns body is believed, remembered to be like?”* It is referred to a cognitive reflection about the body.	Body structural description, general semantic knowledge, formation of attitudes and emotion toward the body.	***Cognitive Representations:** E*specially in left hemisphere.

*Adapted and modified from Longo et al. (2010). Substantial differences exist between the two high-order processes represented by the somatoperception and somatorepresentation, from the basics mechanisms of the somatosensations (Longo et al., 2010). Despite these three different mechanisms are integrated and linked (Moseley et al., 2012), they may be dissociable, at least partially, because each one is based on different functional and neuroanatomical underlying structures and mechanisms: see Longo et al. (2010) for a review on the neural bases of body representations and Medina and Coslett (2016b) for descriptions of clinical cases highlighting this dissociation*.

### Searching Strategy and Information Sources

In line with the published protocol, we followed a three-step search strategy as recommended by The Joanna Briggs Institute (2015). A preliminary search strategy was developed, pilot-tested and peer-reviewed by two authors with different background (a physiotherapist expert in research methodology—DR, and a neuropsychologist—AG), by using the PRESS 2015 Evidence-Based Checklist (McGowan et al., 2016). Modifications to the search string were made after reviewers' suggestions. Searching history and the peer-reviews of the search strategy are available under request. In addition to the procedure described in the protocol, the “Similar Articles” function of PubMed was used and the snowball technique adopted when additional articles were found (Greenhalgh and Peacock, 2005).

#### Electronic Databases

Electronic search was conducted by one author (AV) between May 2018 until September 2018 on 10 electronic databases and grey literature (a full description is provided in the Additional File 2). Very broad search terms were employed for a more sensitive rather than specific search of the literature aimed at meeting the primary goal of the scoping review to systematically map the literature. A secondary review was made by scanning the Gray Matters Checklist.^1^

### Study Selection

**Figure 2:**
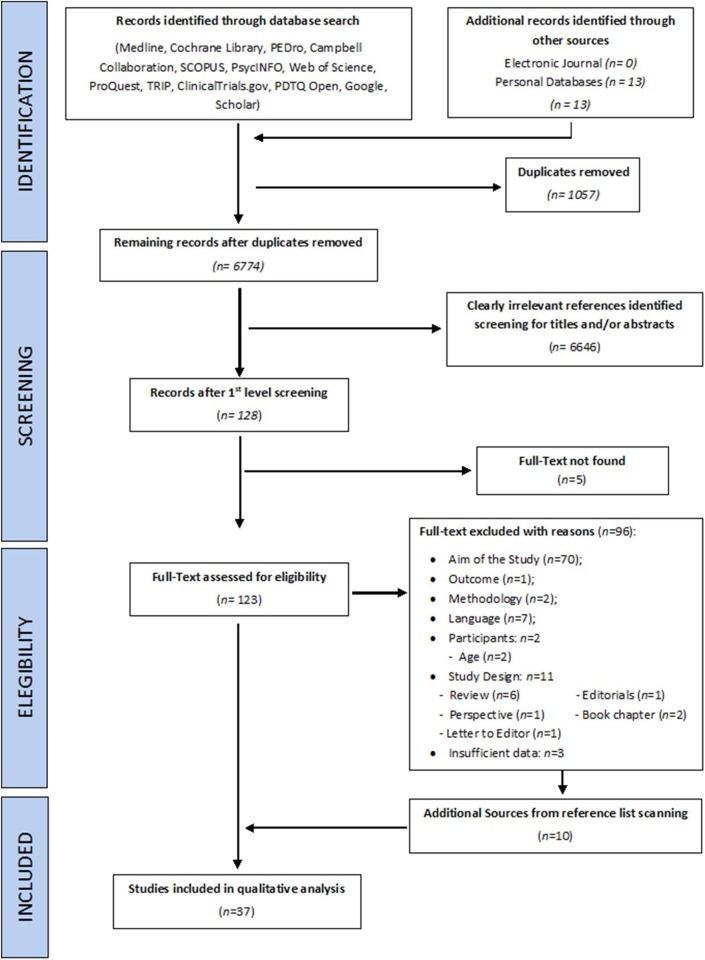
PRISMA flow diagram detailing the selection of sources of evidence.

Two reviewers with different background independently evaluated records for eligibility of title/abstract (DL and EC) and full texts screening (AV and MP). Any disagreement was resolved by discussion between reviewers or, in case of persistent disagreement, a third reviewer (MT) was introduced to reach a consensus. The reviewing process is detailed on the PRISMA flow chart (Figure 2).

### Calibration Phase

Both for the screening of titles/abstracts and for the selection of full-texts the raters performed a series of pilot tests as a calibration exercise to improve the reliability of judgments and agreement between evaluators (Tricco et al., 2018). Each round of pilot testing was accompanied by explanatory documents in which eligibility criteria were updated and clarified, and specification about potentially conflicting terminology were provided in order to avoid interpretation errors. For all pre-formal screening test, inter-rater percentage agreement had to be >90% before starting the formal screening (Colquhoun et al., 2017) (further information are provided in the published protocol). Feedback from evaluators were used to refine the inclusion/exclusion criteria (see the difference between Table 2 in the present study and its counterpart in the published protocol).

**Table 2:** Eligibility criteria for inclusion and exclusion studies.

**Inclusion criteria**	**Exclusion criteria**
**Aim:** studies investigating alterations of the implicit/explicit body perception or perceptual dysfunctions of peri-/extra- personal space. Studies investigating the body ownership (e.g., the rubber hand illusion phenomena). Intervention studies on specific form of perceptual training (e.g., localization sensory training) or involving the perceptual manipulation of body parts as the main content of the therapy (>50%). Intervention studies not adopting perceptual rehabilitation or perceptual manipulations were included only if they considered the effectiveness of proposed interventions with respect to objectively or subjectively measured body perception dysfunctions, as a primary or secondary outcome. **Language:** full text in English Language^a^. **Setting:** experimental or clinical^b^. **Participants:** • Studies on humans (>16 y old), male and female. • Patients affected by musculoskeletal disorders or rheumatic diseases (e.g., LBP, neck pain, osteoarthritis, rheumatoid arthritis, fibromyalgia, etc.), including radicular syndromes (radicular pain and radiculopathies). **Study Design:** • Primary research studies: ∘Quantitative design (including proceedings^c^, conference abstracts^c^): - Experimental designs (RCT, controlled clinical trials); - Observational designs (descriptive studies, surveys, cohort studies, cross-sectional studies, observer-reported or patient-reported outcome studies, case studies^d^/series^d^, proceedings^d^; ∘ Qualitative designs: all types of qualitative research designs. • Secondary research studies^e^: systematic review with or without meta-analysis, meta-summary, meta-synthesis. **Outcomes:** • Quantitative research designs: ∘ Primary Outcomes: - measures, methodologies and tests assessing implicit/explicit body perception dysfunctions and/or alterations of surrounding space perception; self-reported pain, neurophysiologic threshold measures of pain (e.g., electrical pain threshold or pain pressure threshold);-the association between pain (intensity and duration) and disturbances of the implicit/explicit body perception, space perception and body ownership. ∘Secondary Outcomes: - self-reported disability and measures of physical functionality; - the association between neuro-anatomical and/or neurophysiologic correlates and measures of body perception. • Qualitative research designs: ∘ Primary Outcomes: - Interpretation of body image drawings; - the frequency and typology of words used by patients in describing the alterations of own implicit and explicit body experience. Themes and subthemes will be derived by the analysis of patients' interview.	**Aim:** studies investigating body perception in relation to action, both in congruent and incongruent conditions (e.g., the illusion of virtual walking, the mirror therapy, the ability to imagine movements of body parts or to mentally rotate body parts as in the case of motor imagery, the left/right discrimination tasks, or the video-interpretation of own's body in dynamic conditions). Studies investigating the body/self-image with the meaning of the satisfaction about own bodily appearance, physical efficacy and general health, or concerning the body esteem, and self-acceptance. Studies investigating the concept of the body awareness or interoception referred as the general a-specific ability to notice subtle internal bodily sensations/states and emotions, or referred to the generic concept of the “mind-body” connection. Studies using body awareness-oriented intervention (e.g., breath relaxation, concentration, body scan) or belong to the broad umbrella of Body Awareness Interventions (BAI). Studies investigating the balance or posturography, tactile acuity (two-point discrimination threshold), joint repositioning error (or repositioning accuracy), sensorimotor mismatch and sensory/sensorimotor training with tasks involving aspects of somatosensation (e.g., tactile acuity training or JPS training). **Language:** full text and abstract not in English Language^f^. **Participants:** • Patients affected by: - neuropathic pain (e.g., Complex Regional Pain Syndrome—CRPS, Phantom Limb Pain) or myelopathies; - eating disorders (e.g., anorexia, bulimia); - psychiatric or neurological conditions (e.g., personality dissociation, somatoform disorders, Body Identity Integrity Disorders – BIID, dementia, Alzheimer and Parkinson diseases, Multiple Sclerosis, Stroke, Cerebral Palsy, Spinal Cord Diseases); - congenital, hereditary or endocrine abnormalities and deformities (e.g., pectus carinatum, phocomelia, acromegaly, gigantism, Marfan Syndrome, benign joint hypermobility syndrome); - neoplastic or post-neoplastic conditions (e.g., breast cancer). **Study Design:** • Narrative review^g^, editorials^g^^,^^h^, commentaries or expert opinion articles^g^^,^ ^h^, point of view^g^, brief communications^g^^,^ ^h^, debate^g^, perspectives^g^, letters to editors^g^^,^ ^h^, correspondences or replies to letters^g^^,^ ^h^, book reviews or chapters^g^, study protocol^h^.

a *Or full text not in English language but with the abstract in English language*.

b *With a particular focus on studies reporting methodologies or test/measures feasible to translate in clinical practice*.

c The correspondent paper were searched where available; included only if the abstract was available and if was described with a rigorous methodology and with clearly reported results on the base of the construct analyzed.

d Included only if it was described with a rigorous methodology, with clearly reported results on the base of the construct analyzed.

e Included studies and reference lists of secondary studied were manually scanned in order to find additional sources.

f Excluded from the analysis but reported in order to provide a general overview of the amount of international literature published.

g Reference list of records of interest for the topic were be manually scanned.

h *Included in qualitative analysis only if containing additional information to an earlier or ongoing trial study report, or information about a study or experiment not reported elsewhere*.

### Data Analysis

Findings emerged from the retrieved studies were organized around the research questions. Due to the heterogeneity of studies, in terms of research designs, methodological issues and clinical conditions investigated, we adopted a qualitative-descriptive synthesis, as suggested by the PRISMA-ScR (Tricco et al., 2018), and following the approach recommended by the Cochrane Group (Higgins and Green, 2008) and the (Centre for Reviews Dissemination, 2009). In case of incomplete or missing data, the authors of the included papers were contacted for further information. Data were extracted by a single reviewer (AV) using a standardized Excel spreadsheet designed for this study and adapted after the pilot trial charting exercise. A second author (DP) performed the crosschecking of data extracted. Information extracted from each study are detailed in Supplementary Files of the published study protocol. With respect to the original data-extraction form, the item “Future research direction” was deleted because it was not considered relevant The difference between groups means was used as an unstandardized measure for the size of the effect in intervention studies: in case of missed aggregated data, pooled mean and pooled standard deviation were calculated, as well as the 95% Interval Confidence (95% CI). The assessment of studies for clinical relevance was based on the Minimal Clinically Important Difference (MCID) thresholds established in literature for the outcomes used in included studies (see notes of the Supplementary Table S2).

### Critical Appraisal of Individual Sources of Evidence

The primary goal of scoping studies is to systematically map and synthesize results coming from an emerging research area (Canadian Institutes of Health Research, 2010; Colquhoun et al., 2014), rather than provide the best available evidence. Considering also the methodological heterogeneity expected from the studies published on this topic, a qualitative appraisal for risk of bias was not conducted, in accordance to published guidelines on the conduct of scoping reviews (Peters et al., 2015).

### Clinical Relevance of Studies Included in Qualitative Analysis

Clinical relevance was assessed by one author (AV) using the recommendations of the Cochrane Collaboration Back Review Group (Furlan et al., 2009) (a description of the items is provided in Supplementary Table S2). For evaluation studies, the item “Are the likely treatment benefits worth the potential harms?” was replaced with a “The involved procedure/s and/or setting are accessible in terms of cost and advanced technical knowledge required?” and the item “Are the interventions and treatment settings described well enough so that you can provide the same for your patients?” was replaced with “Are the settings and/or the methodology adopted, described well enough so that you can provide the same for your patients?”. Finally, referring to case-controls studies, the item “Is the size of the effect clinically important?” was substituted with “Are there clinically significant differences in the population investigated respect to the control group/s?” In studies without control groups it was used the item “Is there a correlation of the dysfunction detected with at least one out the two clinical variables of pain and disability?” **Figures 7**, **8** summarize the evaluation for clinical relevance, respectively for assessment and for intervention studies.

## Results

### Study Selection

A summary of the main findings is presented in Tables 3, 4, organized with the acronym PCC-Population-Concept-Context (more detailed data can be found in Additional Materials).

**Table 3:** Synopsis of included assessment studies.

**Study**	**Design**	**Population**	**Concept and core outcomes**	**Context and main results**
**CLINICAL SETTING STUDIES**				
***Superficial schema (tactile localisation task)—implicit somatoperception***				
Wand et al. (2013a)	Case-control Study	CLBP patients (*n* = 24) Healthy controls (*n* = 24)	11-NRS (0–10), RMDQ (0–24), Localization task for tactile and painful stimuli (n° of mislocalizations).	67% of subjects with CLBP reported at least 1 mislocalization with respect to 25% of controls (***p=*** **0.034**). Of the possible maximum 28 mislocalizations, five were reported in the worst cases by patients and three by controls. Correlation Analysis: no significant SD were found between mislocalizations errors and other variables, as Pain and Disability.
***Model of body size and shape, and postural schema (body size perception and tactile localization task)—implicit somatoperception***				
Adamczyk et al. (2018a)	Two-Case Report Study	CLBP (*n* = 2)	11-NRS (0–10), ODI (0–100%), 2-PET (mm) for subject A and B; PTP test (mm) for subject A; qualitative version of the PTP test for subject B.	Patient A) overestimated the painful site compared to all non-painful locations 2-PET; range: 45–206%, and also at PTP test: 24–84%. Subjects, by contrast, underestimated the distance at the 2-PET; range: 12–22% smaller than contralateral side.
***Model of body size and shape (body size estimation)—implicit somatoperception***				
Adamczyk et al. (2018b)	Preliminary Validation Study	CLBP patients (*n* = 20)	11-NRS (0–10), ODI (0–100%), Two-Point Estimation (TPE) Task (manual and verbal version).	CLBP patients underestimated the caliper distance by 56.2% (manual version) and 45.9% (verbal version), irrespective of the examiner and location. Reliability: the manual version was more reliable than the verbal one: Inter-rater agreement: manual TPE (ICC = 0.75–0.91); verbal TPE (ICC: 0.53–0.88). Measures in manual version reach a stability after two repetition at painful side: ICC = 0.91 (0.77–0.97). Inter-examiner agreement was god to excellent for manual version (ICC = 0.75–0.91). Intra-rater agreement was good to excellent both at two-day interval (ICC = 0.75–0.91) and at 10-min interval (ICC = 0.66–0.96). In regression Analysis pain duration and pain intensity accounted for 42% of the total variance.
***Model of body size and shape (letter recognition task)- implicit somatoperception***				
Wand et al. (2010)	Case-control Study	CLBP patients (*n* = 19) Healthy controls (*n* = 19)	11-NRS (0–10), SF-36: item 3—physical function (10–30), Letter recognition error rate (n°).	Letter error rate were significantly larger in CLBP group of about 10% (***p=*** **0.016**). No significant correlations were found between Letter error rate and 2-PDT in LBP group (raw data N.R.), nor between Letter error rate and any clinical data (*p*>0.094).
***Depictive methods (body image drawings)—explicit somatoperception***				
Moseley (2008)	Exploratory case-control study	CLBP patients (*n* = 6) Subjects with upper limb pain (*n* = 10)	101-VAS (0–100 mm), Clinical interview and Body Image Drawing of the trunk.	Five out of the six patients reported difficulties in delineating the full extent of their trunk: they all verbatim refer that they “can't find it.” Two subjects reported that “It feels as though it has shrunk.” No patients drew all vertebrae and missing vertebrae coincided with the level of the lost trunk delineation and of the usual pain. There was a tendency of vertebrae displacement from the midline in body drawings.
Lauche et al. (2012a)	a) Qualitative study embedded in a RCT (Lauche et al., 2012b)	CNP patients (*n* = 6)	Themes and sub-themes emerged from interviews in which patients were asked to talk about their body image drawings, Visual interpretation of the Body Image Drawing for neck and shoulders (modified version of that described by Moseley, 2008).	Interviews: patients refer changes in body perception of the neck as a feeling of swollen or distorted in proportion. These overestimations persist even when patients were aware of their actual appearance. Body Image Drawing: at the baseline, the drawn body showed noticeable discrepancies respect to a “normal” body (missing lines and augmented dimension of shoulders and neck) in 4 out 6 subjects more symmetric and complete.
Mibu et al. (2015)	Case-control study	CNP patients (*n* = 20) Healthy controls (*n* = 20)	101-VAS (0–100 mm), Visual interpretation of the Body Image Drawing for neck and shoulders (modified version of that described by Moseley, 2008).	Body image is significantly (***p=*** **0.0017)** distorted in neck pain patients (50%) than in healthy controls (5%).
Nishigami et al. (2015)	Case-control study	CLBP patients (*n* = 42) Healthy controls (*n* = 17)	101-VAS (0–100 mm), RMDQ (0–24), Body Image Drawing of the trunk as described by Moseley (2008). Moreover, subjects were asked to judge the perceived image of their trunk as “normal,” “expanded,” or “shrunken.”	42.8% of subjects with CLBP had a normal perceive image of the lower back, 28.5% had an expanded image, and 28.5% had a shrunken image. There was no significant differences for VAS scores, pain duration, RMDQ, and PCS scores between three perceived image subgroups; *p*> 0.127.
Moreira et al. (2017)	Exploratory case-control Study	CNP patients (*n* = 7) Healthy controls (*n* = 7)	11-VAS (0–10 mm), Modified version of the Body Image Drawing of the trunk as described by Moseley (2008).	Qualitative analysis of the body image drawing: In both groups, two subjects were not able to draw one side of the neck: comparing the drawings it seems that patients delineate neck and shoulders outline less symmetric and uniform than controls, and necks appear shorter. Moreover, two participants drew neck and shoulders more enlarged than they really were, and these perceptions coincided with pain location. Participants that not draw part of the neck, or drawing a clearly distorted neck tend to report pain of higher level and/or duration.
***Qualitative studies—explicit somatoperception***				
Valenzuela-Moguillansky (2013)	Qualitative Study	FM patients (*n* = 12)	Themes and sub-themes.	Interviewees refer modifications in different aspects of body perception: body size, weight, localization and ownership. They talk about enlarged, thicker and heavy body parts. They also refer that near space is perceived as smaller, as if it was shrinking while their body become larger. At the peak of the pain stage some patients described the perception that the painful body parts did not belong to them (loss of the sense of body ownership), expressing the paradoxical experience of being in extreme pain while not feeling it. Moreover, they refer the inability to localize their painful body parts and pain.
***FreBAQ—explicit somatoperception***				
Wand et al. (2014)	Psychometric Validation Study	CLBP patients (*n* = 51) Healthy controls (*n* = 51)	101-VAS (0–100 mm), RMDQ (0–24), FreBAQ (0–36).	Fifty of 51 (98%) CLBP patients endorsed some level of distortion in self-perception, with only one subject recording zero for all items. FreBAQ mean total mean score in CLBP patients was 10.8 (range = 0–26), in healthy subjects was 0.5; Median Difference: 11; ***p<*** **0.001**. FreBAQ score was clinically correlated with pain duration [ρ = 0.357), pain intensity *[r* = 0.400] and disability [*[r* = 0.365]: overall ***p<*** **0.05**.
Wand et al. (2016)	Cross-sectional Study	CLBP patients (*n* = 251)	11-NRS (0–10), RMDQ (0–24), FreBAQ (0–36).	FreBAQ mean total score in CLBP patients was 9.8 (SD = 6.6); median score = 9.0 (IQR = 4.0–14.0) and it was correlated with disability (0.319; ***p<*** **0.001**) and pain intensity (0.265); ***p<*** **0.001**) in bivariate association**.
Beales et al. (2016)	Case-control questionnaire based study	Post-Partum LPP patients (*n* = 24) Women with no post-pregnancy pain (*n* = 26)	Short-form MGPQ (0–45), ODI (0–100%), FreKAQ (0–36)	FreBAQ median difference: 6 (‡) between Moderate Disability sub-groups and pain free controls; ***p=*** **0.02**; Difference in others group comparison were not statistically significant
Wand et al. (2017)	Exploratory cross-sectional questionnaire based study	Pregnancy-related LPP patients (*n =* 42)	11-NRS (0–10), PGQ (0–100), FreBAQ (0–36).	FreBAQ median difference between pain and pain-free groups: 2.5; (‡); ***p=*** **0.005**.FreBAQ score was significantly associated with pain intensity (*r =* 0.378; ***p=*** **0.027**) but not with disability (*r =* 0.256; *p =* 0.143).
Nishigami et al. (2018)	Psychometric Validation Study	CLBP patients (*n =* 100)	101-VAS (0–100 mm), RMDQ (0–24), FreBAQ (0–36).	FreBAQ mean total score in CLBP patients was 11.7 (6.4), and it was significantly correlated with pain in motion (*p* = 0.25) and disability (*p* = 0.36), **overall** ***p*** **<** **0.05**. The questionnaire showed excellent 2w Reliability (*n =* 40): ICC_3, 1_ = 0.81 (0.67 to 0.89).
Janssens et al. (2017)	Psychometric Validation Study	CLBP patients (*n =* 73) Healthy controls (*n =* 73)	11-NRS (0–10), ODI (0–100%), FreBAQ (0–36).	FreBAQ mean total score in CLBP group (*n =* 73*)* was 11 points (7) and median score = 3 (IQR = ±9) in control group (*n =* 73*)*; ***p=*** **0.001**. Sub-groups analysis revealed that patients with higher disability (ODI ≥20%) scored significantly higher on FreBAQ with respect to those with lower level (ODI <20%: 13 (8) vs. 8 (6); ***p=*** **0.005**. FreBAQ was significantly correlated with ODI (rho = 0.30; ***p=*** **0.010**). The reliability on 1-w interval was moderate (ICC_2, 1_= 0.69; 0.51 to 0.82), however the MDC (95%) was the 30% of the scale (10.8 points), referring to a non-sufficient measurement error.
Ehrenbrusthoff et al. (2018)	Psychometric Validation Study	CLBP patients (*n =* 35) Healthy controls (*n =* 48)	Short Form BPI: Pain Severity (0–10), Pain Interference (0–7), RMDQ (0–24), FreBAQ (0–36).	Global FreBAQ mean total score was significantly different in CLBP group (*n =* 35*)* respect to control group (*n =* 48*)*: 8.8 (6.1) vs. 4.0 (3.3); ***p=*** **0.001**. MD adjusted for Age, Gender and BMI = 5.4 (3.0 to 7.8); ***p<*** **0.01**. The 1w-Reliability and Inter-observer reliability were good: ICC for absolute agreement were, respectively, 0.88 (95% CI: 0.77–0.94) and 0.88 (95%CI: 0.75–0.94). FreBAQ score was significantly correlated with Pain (BPI-Pain Interference: *rs =* 0.47; ***p<*** **0.001**) and Disability (RMDQ: *rs =* 0.46; ***p<*** **0.001**).
***FreKAQ—explicit somatoperception***				
Nishigami et al. (2017)	Psychometric Validation Study	Knee OA patients (*n =* 65) Healthy subjects (*n =* 65)	101-VAS (0–100 mm), OKSQ (0–48), FreKAQ (0–36)	FreKAQ mean total score was significantly higher in knee patients vs. healthy controls: 12.4 (7.6) vs. 3.4 (4.4); (***p=*** **0.001**). The reliability at 2 w was good ICC (*n =* 23): 0.76 (0.52 to 0.89). FreKAQ score was significantly correlated with Pain during motion (101-VAS: rho = 0.37; ***p=*** **0.002**) and Disability (OKSQ: rho = −0.41; ***p=*** **0.001**).
***Neglect-like symptoms questionnaire—explicit somatoperception***				
Magni et al. (2018)	Case-Control Study	Hand OA Patients (*n =* 20) Healthy subjects (*n =* 19)	11-NRS (0–10), DASH (0–100), NLSQ (5–30).	The hand OA group reported neglect-like symptoms (median score: 5.5; IQR: 3) significantly *(**p<*** **0.001**) more often than the control group (median score: 5; IQR: 0), however the difference was very low: 0.5 points (‡); χ^2^(1)=12.8; Cramer's V =0.6.
Hirakawa et al. (2014)	Longitudinal Study	Total Knee Arthroplasty for Knee OA patients (*n =* 90)	101-VAS (0–100 mm), NLSQ (0–500): Motor Neglect (MN) and Cognitive Neglect (CN) sub-scales.	The percentage of patients with a total NLSQ ≥100 was 36% (MN, 40%; CN, 18%) at 3 w and 19% (MN, 19%; CN, 5%) at 6 w. The MN subscale of NLS was associated with Pain at 3 w (β = 0.50; ***p<*** **0.01**) and 6w (β = 0.53; ***p<*** **0.01**). The total score of NLSQ (for both MN, and CN sub-scales) decreased at 6 w from 77.7 (87) to 41.2 (62.1); Cronbach's α ≥0.92 (+) for the total score; however, the SD was high, indicating a large variation among patients.
**EXPERIMENTAL SETTING STUDIES**				
***Visual estimation task—explicit somatoperception***				
Gilpin et al. (2015)	Case-control Study	Hand OA patients (*n =* 12) Healthy controls (*n =* 12)	Visual size estimation task (% of the real hand size).	Hand size estimations were significantly smaller for the OA group: −8.01 (3.07 to 12.94); t(22) = 2.39, ***p=*** **0.026**, indicating an underestimation of hand dimensions.
***RHIP—body ownership***				
Martínez et al. (2018)	Case-control Study	FM patients (*n =* 14) Healthy controls (*n =* 13)	11-VAS (0–100 mm), Short-form BPI (0–20), FIQ (0–100), 5-point Likert Scale measuring proprioceptive drift (0.35), ownership (0–35) and agency (0–30).	FM patients were more prone to experiment the misperceptions produced by the RHIP. They scored significantly (***p<*** **0.05***)* higher in all 5-items of the proprioceptive drift scale and in 4 out 5 items of the agency scale (Effect Size varying between 0.88 and 3.10): differences were largest in the proprioceptive drift domain, where large effect sizes were found across all items.
***Perception of subjective visual vertical/ horizontal—extra-personal space perception***				
Treleaven and Takasaki (2015)	Case-control Study	CNP patients (*n =* 36) WAD patients (*n =* 42) Healthy controls (*n =* 48)	11-NRS (0–10), NDI (0–100%), Short Form DHI (0–13), SVV: Computerized Rod And Frame (CRAF) test as described by Takasaki et al. (2012). Error calculation: mean AE (°), mean VE (°), mean CE (°), mean RMSE (°).	CNP group had significantly larger variability of SVV errors vs. the other two groups: 1) VE = 0.5 (0.23 to 0.77) vs. healthy controls (***p=*** **0.001**); 0.37 (0.07 to 0.67) vs. WAD (***p=*** **0.02**); 2) RMSE = 0.51 (0.09 to 0.93) vs. healthy controls (***p=*** **0.01**); 0.58 (0.20 to 0.96) vs. WAD (***p=*** **0.01**). The AE and DE were not able to detect group differences (*p*-value respectively of 0.06 and 0.99). Despite the higher level of disability of the WAD group, there were no significant differences in SVV error between this group and healthy subjects (*p =* 0.91). SVV errors and Disability (DHI) seemed to be unrelated, where a sample of the scatter plot for VE is presented (raw data N.R.).
Docherty et al. (2012)	Case-control Study	CNP patients (*n =* 50) Healthy controls (*n =* 50)	11-NRS (0–10), NDI (0–50), SVV and SVH: Computerized Rod And Frame (CRAF) test (°).	In absence of surrounding frame, significant difference were found in mean errors *(**p<*** **0.05**) both for SVV and SVO test between groups, however they fell within a range considered normal (<0.5°). Significant between-groups difference both for the SVV and SVO (***p<*** **0.001** in all cases) was recorded in tilting the frame clockwise or counter clockwise by 18°, although the difference between the medians values for these tests were still small (<2°). Of the 50 CNP patients, a subgroup of 8 subjects (16%) exhibited higher than normal errors in both the SVV and SVO: these patients scored higher on the NDI than patients whose errors fell within the reference range (*U* = 74.0, *p < * 0.016).
(Grod and Diakow, 2002)	Cohort study	Acute or recurrent NP patients (*n =* 19) Healthy controls (*n =* 17)	Computerized Rod And Frame (CRAF) test as used by Docherty et al. (2012). SVV (°) and SVH (°).	Statistically significant differences in SVV and SVO were found between symptomatic and asymptomatic subjects (F = 13.37, p = **0.001**); pooled Mean Difference = 1.99° (pooled SD = 1.61).

ACR, American College of Rheumatology; CLBP, Chronic Low Back Pain; LPP, Lumbo-Pelvic Pain; FM, Fibromyalgia; OA, Osteoarthritis; NP, Neck Pain; CNP, Chronic Neck Pain; VAS, Visual Analog Scale; NRS, Numeric Rating Scale; Chronic Pain Grade, CPG; BPI, Brief Pain Inventory; MGPQ, McGill Pain Questionnaire; ODI, Oswestry Disability Index; RMDQ, Roland Morris Disability Questionnaire; DASH, NDI, Neck Disability Index; DHI, Dizziness Handicap Inventory; BPQ, Body Perception Questionnaire; Disabilities of the Arm, Shoulder and Hand Questionnaire; PGQ, Pelvic Girdle Questionnaire; TSK, Tampa Scale of Kinesiophobia; PCS, Pain Catastrophizing Scale; FPQ, Fear of Pain Questionnaire; HADS, Hospital Anxiety and Depression Scale; BDI, Beck Depression Inventory; DASS, Depression Anxiety Stress Scale; STAI, State-Trait Anxiety Inventory; FIQ, Fibromyalgia Impact Questionnaire; SIQ, Symptoms Impact Questionnaire; OKSQ, Oxford Knee Score Questionnaire; MAIA, The Multidimensional Assessment of Interceptive Awareness; DSM-IV, Diagnostic and Statistical Manual of Mental Disorders IV; PU, Pain Unpleasantness; PI, Pain Intensity; PeT, Perception Threshold; PT, Pain Threshold; PTo, Pain Tolerance; FreKAQ, Fremantle Knee Awareness Questionnaire; NLSQ, Neglect-like symptoms questionnaire, TPT, Tactile Perception Threshold; 2PDT, 2-Point Discrimination Threshold; PTP, Poin-to-Poin Test; 2-PET, two Point Estimation Test; ROM, Range Of Motion; JPS, Joint Position Sense; TEMPA, Test d'Evaluation de la performance des Membres Supérieurs des Personnes Agées; BMI, Body Mass Index; RHIP, Rubber Hand Illusion Paradigm; SVV, Subjective Visual Vertical; SVH, Subjective Visual Horizontal; AE, Absolute Error; VE, Variable Error; AE, Absolute Error; VE, Variable Error; AE, Absolute Error; VE, Variable Error; DE, Direction of Error; RMSE, Root Mean Square Error; MDC, Minimal Detectable Change; ICC, Interclass Correlation Coefficients; MD, Mean Difference; NR, not reported; NA, Not Applicable; SD, Standard Deviation; IQR, Interquartile Range; SEM, Standard Error of Measurement; CI95%, Confidence Interval; +, p-value not reported; ‡, 95% CI not reported; SD, statistical difference; y, year/s; m, month/s; h, hour/s, min., minute; s, second/s, ms, millisecond. ***p-value***is reported in bold if statistically significant; **sub-groups were obtained through the median split with established ODI categorisation.

**Table 4:** Synopsis of included intervention studies.

**Study**	**Design**	**Population**	**Concept and core outcomes**	**Context and main results**
**CLINICAL SETTING STUDIES**				
***Tactile localization training***—***implicit somatoperception***				
Wand et al. (2013b)	Randomized COT	CLBP patients (*n =* 24)	1) Patients were assigned to: 2) acupuncture treatment involving sensory discrimination training (single session); a true acupuncture treatment (single session); 11-NRS (0–10) after performing 10 repeated spine movement in the most provocative direction reported in the initial physical examination.	a) Pain was significantly lower in both groups at the end of treatment, regardless of treatment order (*p =* 0.182), but the magnitude of pre-post treatment change was not clinically significant: 11-NRS = −0.9 (−0.3 to −1.5), *p =* **0.008**. b) Pain was lower in EG but the magnitude of change was not clinically relevant: 11-NRS: −0.8 (−1.4 to −0.3) in favour to EG, ***p=*** **0.011**.
Louw et al. (2017)	Case-series	CLBP patients (*n =* 16)	It was administered a perceptual localization task of 5 min. in a single session. 11-NRS (0–10), Functionality: active lumbar flexion (cm), FABQ (0–92).	a) Pain was lower after treatment (11-NRS: –**1.9**; (‡) range: 0 to 6; +) but the magnitude of pre-post treatment change was not clinically significant. Functionality improves after treatment with clinical significance (**4.8**; ‡; range: −1 to 21; +).
Barker et al. (2008)	Single-blinded, RCNIT	CLBP patients (*n =* 60)	It was tested the non-inferiority of the FairMed (device for sensory discrimination training), administered for 30 min (twice a day, for 3 weeks), respect to the TENS (same dosage). 0–11 VAS (0–10 mm) as a mean of patients' present pain intensity level, their average and worst pain intensity levels recorded over a week, ODI (0–100%), Physical Functioning: 5 min walking distance (5'-WD), 1 min stair climb (1'-SC) and 1 min sit-to—stand (1'-STS).	a) Both groups improved in pain scores: 0–11 VAS = −0.8 (−1.5 to −0.1) for EG; –**7.3** (−8.1 to −6.6) for CG but the differences were not statistically significant (*p =* 0.83). The same positive trend was recorded for disability: ODI = −0.6 (−3.8 to 2.7) for EG, and −0.9 (−3 to −1.1) for CG. Even in this case the difference was neither statistically (*p =* 0.85), nor clinically significant. All other functional measures (5'-WD, 1'-SC, 1'-STS) improved in both groups, without statistically significant difference between pre- and post- recordings (*p*>0.05). FairMed device was not inferior respect to TENS. There were minimal and no statistically significant difference between groups both for pain (0–11 VAS: −0.1; −0.7 to 0.3; *p =* 0.82) and disability (ODI: 0.4; −0.7 to 0.4; *p =* 0.85). The same trend was recorded for all the other functional measures 5'-WD, 1'-SC, 1'-STS, without statistically significant differences.
***Mixed perceptual training (graded perceptual training+graded motor retraining)***—***implicit somatoperception***				
Wand et al. (2011)	Three single-case study	CLBP patients (*n =* 3)	It was tested a mixed treatment program composed by education and graded perceptual retraining program (localization and graphaestesia training) combined with graded motor retraining (minimum 10 w of home exercises). 11-NRS (0–10), RMDQ (0–24).	Both Pain and Disability improves with clinical and statistical significance: 11-NRS = –**2.9**; (1.2 to 4.6) at T1; ***p<*** **0.001**; –**3.9**; (1.6 to 6.2) at T2; ***p<*** **0.001;** RMDQ = –**5.2** (2.4 to 8) at T1; ***p<*** **0.001**; –**9.6** (4.2 to 15) at T2; ***p<*** **0.001**.
(Ryan et al., 2014)	Mixed-methods pilot RCT	CLBP patients (*n =* 24)	Patients were assigned to: 1) EG: combined tactile acuity and graphaestesia acuity training as used by Wand et al. (2011), plus usual physiotherapy (3 sessions + 21 at home); 2) CG: tactile stimulation alone (placebo) plus usual care (same dosage). 101-VAS (0–100 mm), RMDQ (0–24).	Tactile acuity training was not superior to sham therapy. Both groups improved pain and disability post-treatment but only values for CG were statistically significant (101-VAS: –**33.2**, CI95%: −58.3 to −8; RMDQ: –**4**; CI95%: −6.7 to −1.3); ***p<*** **0.05)**. Between-groups comparison was in favour to sham group both for both 101-VAS (25.6; −0.7 to 51.9) and RMSQ (2.2; −1.6 to 6.0), despite with no statistical significance (*p =* 0.056 and *p =* 0.237 respectively).
***Mixed perceptual training (SuPerR Treatment)***—***implicit Somatoperception***				
Morone et al. (2012)	Single-blinded, RCT	CLBP patients (*n =* 75)	Patients were assigned to: 1) EG: SuPeR treatment (perceptive surface plus active exercises, 45', 3 x week for 1 month, and usual pharmacological care); 2) CG1: Back school program (10 session for 1 m, and usual pharmacological care), CG2: Medical and pharmacological assistance only (as the other two groups). 11-VAS (0–10 mm), ODI (0–100) post-treatment (T1), at 12 w (T2) and 24 w (T3).	Both groups treated with SuPeR and with Back School obtain an overall improvement both for Pain and Disability at T1 and T3: 11-VAS = –2* (‡) (***p<*** **0.001**) at T1 and T3 for EG; −1* (‡) at T1 and –3* (‡) at T3 (***p<*** **0.001**) for CG; ODI = –18* (‡) at T1 and –14* (‡) at T3 for EG; −10* at T1 (‡) and –16* (‡) at T3 for CG (***p<*** **0.001**). CG2 maintained substantially unaltered the level of Pain and Disability at T1 and T3 (*p>*0.05). EG patients recorder statistically less Pain at T1 respect to CG1 (11-VAS: −1*; ‡) and CG2 (11-VAS: –3*; ‡) (***p<*** **0.001)**, but at T3 the improvement was in favour to CG1 (11-VAS: 2*; ‡; ***p<*** **0.001**), despite the magnitude of the effect was not clinically relevant. The effect of the EG for Disability at T1 was not statistically different respect to CG1 and CG2 (*p =* 0.403). At T3 EG reduced Disability significantly lower than CG2 (ODI: –16*; ‡; ***p=*** **0.023**), but no difference was found respect to CG2 (ODI: −2*; ‡); (p = 0.169).
Paolucci et al. (2012)	Single-blinded, RCT	CLBP patients (*n =* 45)	Patients were assigned to: 1) EG: SuPeR: the same protocol of Morone et al. (2012); 2) Back school program with: the same protocol of Morone et al. (2012). MGPQ (0–78).	The SuPereR treatment reduce pain more than the CG performing the Back School program, but the difference was not statistically significant: MGPQ = 44 ± 24% for EG^†^ and 39 ± 15% for CG^†^; *p =* 0.436. ^†^Authors reported the % improvement with respect to the maximum achievable improvement.
Vetrano et al. (2013)	Single-blind, RCT	CLBP patients (*n =* 40)	Patients were assigned to: 1) EG: modified SuPeR treatment (more deformable cones at midline level, decreasing tactile-pressure inputs at midline level and without taking consciousness of the body midline; 2) Standard SuPeR treatment: the same protocol of Morone et al. (2012) but with 5' less of treatment duration and with an increase of tactile-pressure inputs at midline level. 11-VAS (0–10 mm), ODI (0–100), post-treatment (T1), at 4 (T2) and 12 weeks (T3).	Both groups improved pain and disability scores at T1 and T3 respect to the baseline: 11-VAS: −2* (‡) at T1, –3* (‡) at T3 (***p<*** **0.001**); ODI: –14* (‡) at T1, –20* (‡) at T3 (***p<*** **0.001**) for EG. 11-VAS: –2.5* (‡) at T1, –5.5* (‡) at T3 (***p<*** **0.001**); ODI: –16* (‡) at T1, –21* (‡) at T3 (***p<*** **0.001**) for CG. The modified SuPerR treatment was substantially as effective as the standard version both for pain and disability: 11-VAS and ODI scores were lower for CG at T1 and T3 but differences were not statistically significant: 11-VAS: −2* (‡) at T1 (*p =* 0.179), 2.5* (‡) at T3 (*p =* 0.868); ODI: 2* (‡) at T1 (*p =* 0.299), 1* (‡) at T3 (*p =* 0.922).
***Qualitative Studies***—***Explicit Somatoperception***				
Lauche et al. (2012a)	Qualitative study embedded in a RCT (Lauche et al., 2012b)	CNP patients (*n =* 6)	Patients were interviewed before and 3 d after a single traditional cupping treatment (for half of the patients); the other half of patients were in waiting list. Themes and sub-themes emerged from interviews in which patients were asked to talk about their body image drawings, Visual interpretation of the Body Image Drawing (modified version for trunk as described by Moseley, 2008).	Interviews: subjects in EG refer a reduction in neck size (smaller) as a relief from pain.Body Image Drawing: Body image appears to be changed in EG after treatment (smaller dimension of body parts, and lines more symmetric and complete). Even the CG subjects improve in drawings their own's body, but they were no more complete, nor matched a “normal” silhouette.
**Experimental Study**				
***Manipulation of visual body appearance***—***Explicit Somatoperception***				
Preston and Newport (2011)	Exploratory experimental study	Hand OA patients (*n =* 20)	Patients were administered the MIRAGE system: visuo-tactile illusion involving manipulations (stretching or shrinking) of patient's hand (affected and unaffected) while experimenter gently pulling or pushing on part of the hand. 21-NRS (0–20).	85% of patients reported reduction in pain for at least one of the experimental conditions (stretching and shrinking), but only manipulating visual appearance of the affected hand. For subjects in whom the stretching was beneficial the condition produced ~50% on pain reduction (–**3.09**, ‡; +), while for those in whom the shrinking condition was beneficial pain decrease of ~45% (–**2.68**, ‡; +).
Diers et al. (2013)	Experimental study	Bilateral TP patients (*n =* 18) Healthy Controls (*n =* 18)	Patients were administered: 1) EG: an on-line video feedback of the neck in enlarged and downscaled fashion; 2) CG: video feedback of neutral (hand dorsum) body part, and affected neck in unaltered fashion. PU for pressure stimulation applied to the TrP1 of the Trapezius muscle: 11-NRS (0–10), PU and PI for electrical stimulation applied to the TrP1 of the Trapezius muscle: 11-NRS (0–10).	There was no significant influence on Electric and Pressure PU (*p < * 0.986) and PI (*p < * 0.825) for back hand condition (CG). Visual feedback conditions (CG) significantly influenced the Electric and Pressure PU and PI: (***p<*** **0.001**), in Downscaled, Enlarged and Size Control Back condition, even if the differences were never over the clinical significance.
Stanton et al. (2018)	Pilot-experimental study	Knee OA patients (*n =* 12)	Patients were administered the MIRAGE system as described in Preston and Newport (2011), applied to the knee in 8 conditions: congruent (CO) and incongruent (IN) X vision only (VO), tactile only (TO) and visuotactile (VT) X stretch (ST) and shrink (SR); 30s with 2 min. rest. Session 1: Total duration: 1 h. Session 2: the CO condition producing the greatest pain reduction was applied for 3 min (sustained condition-SU) and repeated for 10 trials (repeated condition-RE), minimum 2 w apart. Session 3: the RE condition of the Session 2 was repeated maximum 3 w apart. 101-NRS (0–100) immediately and 48h after Session 2, prior Session 3.	VT illusion decreased pain by an average of 7.8 points (2.0 to 13.5), corresponding to a 25% reduction in pain both in CO (t_1, 11_ =2.96, ***p=*** **0.013**) and in IN conditions. SU condition prolonged analgesia, but did not increase it: (Session 1: t_1, 10_ =0.52, *p =* 0.61; Session 3: t_1, 7 =_-0.697, *p =* 0.51). RE condition (with congruent VT illusion) increased the analgesic effect: 101-NRS: –**20** (−6.9 to −33.1), corresponding to a 40% pain reduction. Between Conditions Difference: The CO-VT condition did not differ from the IN-VT condition, controlled for vision: no effect of Condition (F_1, 11_ =0.032, *p =* 0.86), Condition x Time interaction (F_1, 11_ = 0.34, *p =* 0.57), suggesting that analgesia was provided by both conditions when identical visual manipulations occurred; in contrast CO-VT reduce more pain than IN-VT when controlled for tactile input: Time effect (F_1, 11_ =5.23, ***p=*** **0.043**), Condition x Time interaction (F_1, 11_ = 5.29, ***p=*** **0.042**).
***Manipulation of visual body appearance+cognitive manipulation***—***explicit somatoperception***				
Nishigami et al. (2019)	Pilot-experimental study	CLBP patients (*n =* 2)	Patients were administered the MIRAGE system as described in Preston and Newport (2011), applied to low back during a lifting task in three different conditions. Participants watched: a) a modified version of their back (muscled, fit-looking strong, back); b) - reshaped image of their back; c) a normal shaped condition 101-NRS (0–100), Fear: 101-NRS (0–100).	Visual illusion of a strong back vs. normal condition reduce pain and fear only in subject having distorted explicit perceptual representation of his back (FreBAQ: 29/36): 101-NRS for pain = –**30**; 101-NRS for fear: −12. No significant reduction for pain and fear in the second subject without distorted perceptual representation.

ACR, American College of Rheumatology; CLBP, Chronic Low Back Pain; LBP, Low Back Pain; TP, Thoracic Pain; CNP, Chronic Neck Pain; OA, Osteoarthritis; MPQ, VAS, Visual Analog Scale; 11-NRS, 11-point Numeric Rating Scale; MGPQ, McGill Pain Questionnaire; ODI, Oswestry Disability Index; RMDQ, Roland Morris Disability Questionnaire; TSK, Tampa Scale of Kinesiophobia; RCT, randomized controlled trial; SuPeR, Surface for Perceptive Rehabilitation; TENS, Transcutaneous Electrical Nerve Stimulation; NSAIDs, Nonsteroidal Anti-Inflammatory Drug; TrP, Trigger Point; PU, Pain Unpleasantness; PI, Pain Intensity; PeT, Perception Threshold; PT, Pain Threshold; PTo, Pain Tolerance; RCT, Randomized Controlled Trial; COT, Cross-Over Trial; RCNIT, Randomized Controlled Non-Inferiority Trial; N.R., not reported; SD, Standard Deviation; MD, Mean Difference; CI95%, Confidence Interval; ^*^, median difference; +, p-value not reported; ‡, 95% CI not reported; SD, statistical difference; y, year/s; m, month/s; h, hour/s; EG, Experimental Group; CG, Control Group. **p-values** are reported in bold if statistically significant; other values reported in **bold** if clinically significant based on the established Minimal Clinically Important Difference: for LBP was considered as clinically significant 13 points on the ODI (Copay et al., 2008; Johnsen et al., 2013), 30% on VAS/NRS for pain (Farrar et al., 2001; Ostelo et al., 2008), 2–3 points (or 8–12%) on the RMDQ for function (Bombardier et al., 2001; Ostelo et al., 2008) and 4.5 cm for the Active Lumbar Flexion (Ekedahl et al., 2012). For neck pain, was considered 3.5 to 5 U on the 50-U Neck Pain Disability Index or 7 to 10% change (Pool et al., 2007; Stratford et al., 2009) for function and 2.5 on an 10-U NRS (25% change) for pain (Pool et al., 2007).

**Figure 3:**
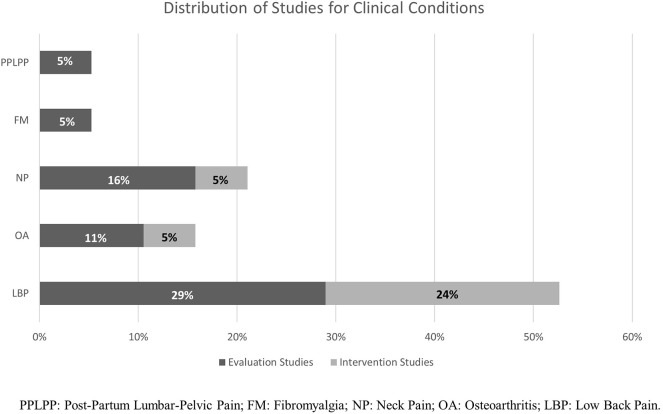
Summary of the distribution of included studies for clinical conditions examined.

**Figure 4:**
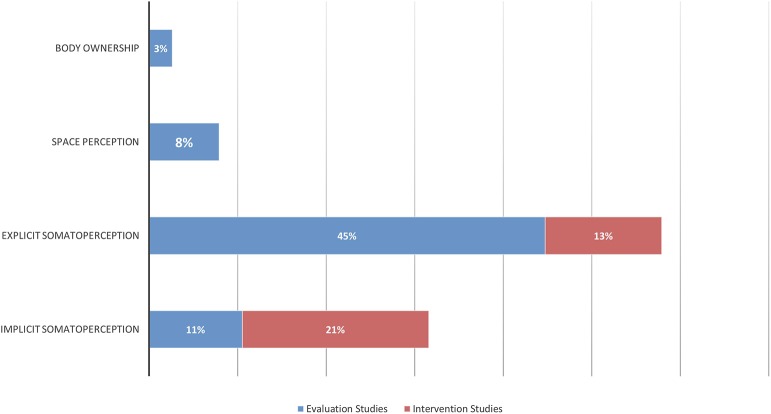
Summary of the distribution of domain investigated for study typology.

The first calibration test was conducted on 239 titles and abstracts, and the second on 12 full-texts: the inter-rater agreement was, respectively, of 93 and 100%, and was reached for both procedures at the third round, at the end of which the reviewers express no need of further training. The search strategies initially produced 7,818 records from all the databases, and 13 from authors' personal databases (Morone et al., 2012; Paolucci et al., 2012, 2016; Wand et al., 2013a; Hirakawa et al., 2014; Louw et al., 2017; Stanton et al., 2017, 2018; Adamczyk et al., 2018a,b; Ehrenbrusthoff et al., 2018; Magni et al., 2018; Nishigami et al., 2019). After removal of duplicates and exclusion of clearly irrelevant records on the basis of the title and abstract, 123 full-texts were screened. Five full-texts were not found and 96 were excluded with reasons (see the Supplementary Table S7). Ten additional studies (Grod and Diakow, 2002; Barker et al., 2008; Wand et al., 2010, 2011; Preston and Newport, 2011; Nishigami et al., 2012; Diers et al., 2013; Ryan et al., 2014; Treleaven and Takasaki, 2015; Beales et al., 2016) were identified and considered eligible by searching the reference lists of included papers and reviews considered of interest for the aim of this work. Thirty-seven studies, analysing an overall sample of 1291 patients (1,094 in evaluation studies, and 197 for interventions ones), were included in the qualitative analysis and the end of the selection process (see the Figure 2 for a flow-chart of the entire process). Agreement between raters in formal screening was 95% for titles/abstracts screening, and 91% in full-texts inclusion: all disagreements were resolved upon discussion and clarification of eligibility criteria, and the intervention of the third independent assessor (MT) was not needed. A graphical distribution of included studies grouped by clinical conditions examined is shown in Figure 3. Twenty-five studies (Grod and Diakow, 2002; Moseley, 2008; Wand et al., 2010, 2013b, 2014, 2016; Docherty et al., 2012; Lauche et al., 2012a; Valenzuela-Moguillansky, 2013; Hirakawa et al., 2014; Gilpin et al., 2015; Mibu et al., 2015; Nishigami et al., 2015, 2017, 2018; Treleaven and Takasaki, 2015; Beales et al., 2016; Janssens et al., 2017; Moreira et al., 2017; Adamczyk et al., 2018a,b; Ehrenbrusthoff et al., 2018; Magni et al., 2018; Martínez et al., 2018) studies concerned the assessment of SoP dysfunctions, while twelve interventional studies (Barker et al., 2008; Preston and Newport, 2011; Wand et al., 2011, 2013a; Morone et al., 2012; Paolucci et al., 2012; Diers et al., 2013; Vetrano et al., 2013; Ryan et al., 2014; Louw et al., 2017; Stanton et al., 2018; Nishigami et al., 2019) investigated the effects of perception-based intervention to reduce pain or to correct perceptual distortions. One study, (Lauche et al., 2012a) was included both in assessment and in intervention studies: it is a qualitative study investigating the explicit SoP in chronic neck pain (CNP) patients at baseline, and also at follow-up because it was embedded in a RCT study investigating the effect of cupping therapy (Lauche et al., 2012b). Figure 4 displays the domains investigated by assessment and intervention studies.

### Research Designs of Included Studies

**Figure 5:**
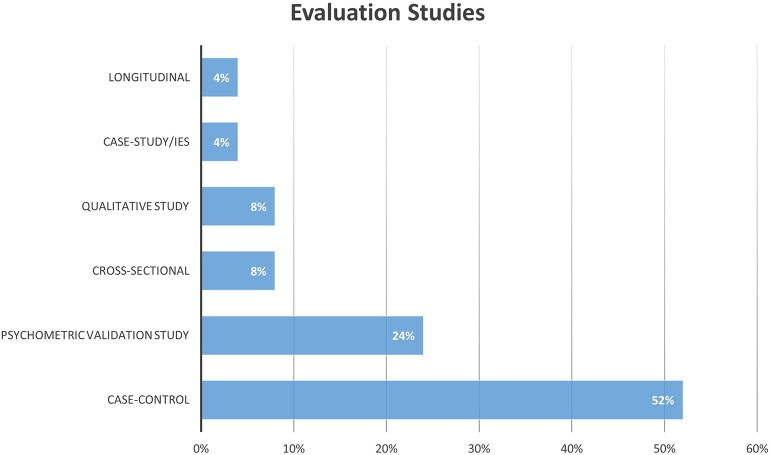
Summary of the distribution of evaluation studies for research design adopted.

**Figure 6:**
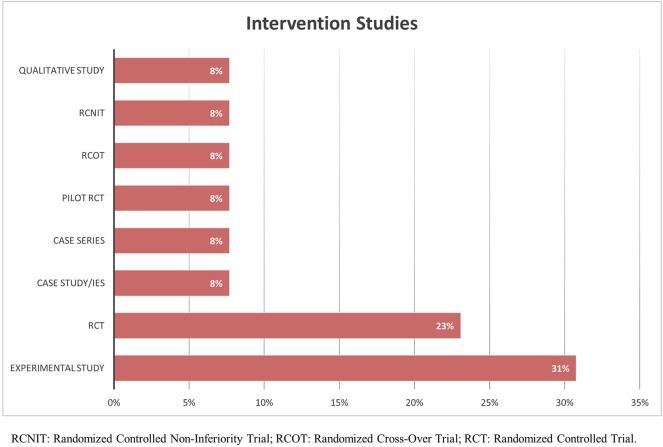
Summary of the distribution of intervention studies for research design adopted.

The distribution of research designs adopted for assessment studies is graphically represented in Figure 5. A conspicuous number of these studies is composed by those validating the Fremantle Back Awareness Questionnaire (FreBAQ) (Wand et al., 2014) and the Fremantle Knee Awareness Questionnaire (FreKAQ) (Nishigami et al., 2017), including validation studies into other languages (Janssens et al., 2017; Ehrenbrusthoff et al., 2018; Nishigami et al., 2018), and cross-sectional or case-control investigations across different clinical samples (Wand et al., 2013a, 2016; Beales et al., 2016). Figure 6 shows the research designs adopted in the intervention studies included, of which a large part were pre-clinical experimental studies (Diers et al., 2013; Nishigami et al., 2019).

### Assessment Studies

Five studies were conducted in an experimental setting (Grod and Diakow, 2002; Docherty et al., 2012; Gilpin et al., 2015; Treleaven and Takasaki, 2015; Martínez et al., 2018), while the remaining 19 studies were clinical investigations (Moseley, 2008; Wand et al., 2010, 2013a,b, 2014, 2016; Lauche et al., 2012a; Valenzuela-Moguillansky, 2013; Hirakawa et al., 2014; Mibu et al., 2015; Nishigami et al., 2015, 2017, 2018; Beales et al., 2016; Janssens et al., 2017; Adamczyk et al., 2018a,b; Ehrenbrusthoff et al., 2018; Magni et al., 2018).

The main domain studied was explicit SoP (58%; 22/38), followed by implicit SoP (32%; 12/38), and SpP (8%; 3/38), while the BO made up only the 2% (1/38) of the sample.

#### Implicit Somatoperception

Only 4/24 (17%) of the included assessment studies investigated implicit SoP. Adamczyk et al. (2018a,b) preliminarily validated a methodology for the objective evaluation of implicit body size perception, the two-point estimation (2-PET) task. Among all the included assessment studies, it is the only one adopting an objective methodology to assess metric features of SoP. Authors found an underestimation of the distance between two tactile stimuli delivered with a caliper on the back (46 and 56%, respectively on the verbal and manual version of test) in both the painful and pain-free low-back side. Duration and pain intensity predicted the presence of perceptual dysfunctions and accounted for the 42% of the total variance of 2-PET scores in the regression analysis. The same 2-PET task were used by the same authors in a two-case report study (Adamczyk et al., 2018a) in which one patient showed an overestimation of the painful side, compared to non-painful locations (range: 45–206%), while a second patient showed the opposite pattern with an underestimation ranging between 12 and 22%. In this double case-study, authors used also another test, the point-to-point test (PTP): the distance error between the site touched by the examiner and that touched by the patient was greater on the painful side than on the pain-free location, for a magnitude of 24–84%. With this second patient, the authors used also a qualitative version of the PTP test: subject were asked to point with a pen to the site stimulated by the examiner. In case of error, the examiner drew the error trajectories directly on the patient's back by moving the pen from the incorrect location indicated by the patient to the correct one: on the painful side all the trajectories were outside the referred symptomatic area and considerably spaced between them, indicating large errors in pointing the site of tactile stimuli.

Only one study (Wand et al., 2013b) adopted the localization task of tactile stimuli to assess the superficial schema. Subjects, after being stimulated by the experimenter with tactile and painful stimuli, were asked to mark on a body chart with 12 pre-defined areas of the trunk and thighs, the perceived localization of the applied stimuli. Authors found that 67% of chronic lower back pain (CLBP) patients made at least one localization error compared to only 25% of healthy controls, but no correlations were found between mislocalization errors and either pain and disability. Of the possible maximum 28 mislocalizations, five were reported in the worst cases by patients and three by controls. The study involved tactile and pinprick stimuli, but the authors reported combined results for both type of stimulations, without differentiating between types of task (*personal communication with authors*).

The Letter recognition task (or graphesthesia) involves the recognition of letters drawn on the skin. This task was tested only in the study of Wand et al. (2010): CLBP patients showed 10% more errors respect to healthy controls (*p* < 0.05), however this score was not correlated with clinical data.

#### Explicit Somatoperception

##### Body image drawing task

The majority of selected assessment studies evaluated the explicit SoP: five studies adopted the Body Image Drawing (BID) task (Moseley, 2008; Lauche et al., 2012a; Mibu et al., 2015; Nishigami et al., 2015; Moreira et al., 2017), eight studies used the FreBAQ (Wand et al., 2014, 2016; Wand et al., 2013a; Beales et al., 2016; Janssens et al., 2017; Nishigami et al., 2017, 2018; Ehrenbrusthoff et al., 2018) or the FreKAQ (Nishigami et al., 2017), two studies used the Neglect-Like Symptoms Questionnaire (NLSQ) (Hirakawa et al., 2014; Magni et al., 2018), one study investigate the visual size estimation in an experimental setting (Gilpin et al., 2015), one study investigated the rubber hand illusion (RHI) (Martínez et al., 2018), and one was a qualitative study on subjectively referred body perception (Valenzuela-Moguillansky, 2013).

Of the five study using the BID task, two were conducted on CLBP patients (Moseley, 2008; Nishigami et al., 2015) and three on Chronic Neck Pain (CNP) patients (Lauche et al., 2012a; Mibu et al., 2015; Moreira et al., 2017). All studies reported distortions in BID in a variable percentage of patients: Moseley (2008) found that five out of six patients with CLBP reported difficulties in drawing their trunk along all the entire extension. Four out of six showed the tendency to draw their vertebrae displaced from the midline toward the painful side. Moreover, two patients reported a feeling of shrunken trunk. In a larger sample of patients (*n* = 42), matched with 17 healthy controls, Nishigami et al. (2015) found distorted BID in about 50% of patients: half of them showed an enlarged image of their back, while the other half drew a shrunken BID. The other 50% of patients had a normal BID. However, the authors found no significant differences in pain intensity, duration, or disability between the three groups. Mibu et al. (2015) found a distorted neck drawing in 50% of patients with CNP (significantly more than the 5% of healthy controls), however there were no differences either for pain duration or intensity within CNP sub-groups, or with respect to healthy controls. Moreira et al. (2017) in their preliminary case-control study found a less symmetric and uniform outline of neck and shoulders in CNP patients than in controls. Two patients drew their neck and shoulders enlarged, while another two were unable to delineate one side of the neck, as well as two subjects in control group. Participants with a clearly distorted neck image or unable to draw body parts, tended to report higher pain intensity and duration. Finally, in the qualitative study of Lauche et al. (2012a) the authors used both interviews and BID. In four out six CNP patients, the qualitative analysis of the drawings showed noticeable discrepancies compared to a normal body silhouette, with missing lines and overestimated size of the neck and shoulders.

##### Visual size estimation procedure

Among the assessment studies analysing the subjective visual appearance of body parts, only that of Gilpin et al. (2015) was conducted in an experimental setting. Patients with hand osteoarthritis were asked to judge what photograph corresponded to their actual hand. Photographs were experimentally manipulated in percentage of the real length dimension: patients significantly underestimated the size of their hand, selecting photos showing hands 8% smaller compared to healthy subjects (99.8% of the real hand dimension in patients vs. to 107.8% in healthy controls). Although the MIRAGE system used in this last study induced a visual illusion correcting the distortion evaluated at the baseline, we considered this study only as an evaluation study because the authors did not provide pain measure, nor disability questionnaire as outcome measure (see Table 2 for exclusion criteria).

##### Self-administered questionnaire

*Fremantle back and knee awareness questionnaire* Seven studies were conducted adopting the FreBAQ validated by Wand et al. (2014, 2016) on CLBP patients (Janssens et al., 2017; Ehrenbrusthoff et al., 2018; Nishigami et al., 2018), in post-partum (Beales et al., 2016) or pregnancy-related pelvic pain (Wand et al., 2017), and knee osteoarthritis (Nishigami et al., 2017). Three studies are psychometric validations of the FreBAQ in other languages (Janssens et al., 2017; Ehrenbrusthoff et al., 2018; Nishigami et al., 2018). Fifty of 51 patients with CLBP (98%) reported some level of misperception, and only one patient recorded zero points (corresponding to no misperceptions). The mean score ranged between 8.8 (Ehrenbrusthoff et al., 2018) and 11.7 (Nishigami et al., 2018) in patients, and 0.5 to 3.3 points in healthy controls (Wand et al., 2014; Janssens et al., 2017; Ehrenbrusthoff et al., 2018) on a 0–36 scale where higher scoring indicating larger number of misperceptions. Ehrenbrusthoff et al. (2018) also reported a significant (*p* < 0.01) mean difference between patients and controls in German population adjusted for age, gender and body mass index (5.4 points; 95% CI = 3.0–7.8). However, this was lower than that found by Wand et al. (2014) of 11 points (*p* < 0.001). In all included studies investigating CLBP there was a significant correlation between FreBAQ score with, pain (intensity, duration or interference) (Wand et al., 2014, 2016; Janssens et al., 2017; Ehrenbrusthoff et al., 2018; Nishigami et al., 2018) and disability (Wand et al., 2014, 2016; Ehrenbrusthoff et al., 2018; Nishigami et al., 2018). Janssens et al. (2017) found also a difference between patients at different level of disability: the sub-group with higher disability (Oswestry Disability Index ≥20%) scored significantly higher (*p* = 0.005) than lower-disability group (13 ± 8 vs. 8 ± 6 points).

Beales et al. (2016) administered the FreBAQ to women with lumbo-pelvic pain (LPP) raised minimum 3 months post-partum, and found significantly (*p* = 0.02) more disturbances in explicit SoP in the moderate disability sub-groups of patients (median score: 8/36 points) than in pain free controls (median score: 2/36 points). They also found more perceptual dysfunctions in moderate-disability sub-groups respect to low-disability patients (median score: 6.5/36 points) and pain free controls (median score: 2/36 points), however there was no statistical significance (respectively, *p* = 0.282 and *p* = 0.095; *personal communication)*. Wand et al. (2017) instead collected data on pregnancy-related LPP (within the 3rd trimester of pregnancy and not over the 38th week): women with pain referred significantly (*p* = 0.005) more perceptual dysfunctions than those pain-free (median score: 3.5/36 vs. 1/36), and authors found a significant correlation (*p* = 0.027) of FreBAQ score with pain intensity (*r* = 0.378), despite it was not correlated with self-reported disability (*p* = 0.143).

Finally, Nishigami et al. (2017) adopted the FreBAQ for knee osteoarthritis patients, validating the FreKAQ. They found significantly (*p* = 0.001) more perceptual dysfunctions in patients than in controls (median difference: 9 points), and a significant correlation (*p* < 0.002) between pain in motion (*rho* = 0.37*)* and disability (*rho* = −0.41), but not with pain duration (rho = −0.06, *p* = 0.76).

*Neglect-like symptoms questionnaire* Two studies using the NLSQ developed by Galer and Jensen (1999) and Frettlöh et al. (2006) in CRPS. The NLSQ measures the cognitive and motor neglect, with higher scoring indicating more neglect referred symptoms. Hirakawa et al. (2014) found that 36% of patients with knee osteoarthritis scored more than 100 on a 0–500 range three weeks after arthroplasty, decreasing at 19% at six weeks (*p*-value not reported). The mean NLSQ score decreased from 77.7 to 42.2 points (*p*-value not reported); however, the standard deviation was high due to a large inter-subject variation. The motor neglect sub-scale (MNss) was associated in multiple regression analysis with pain both at 3 (β = 0.50; *p* < 0.01) and 6 weeks (β = 0.53; *p* < 0.01), where β represents points on MNss per unit of pain intensity, and with the improvement of range of motion at 6 weeks (β = −0.28; *p* < 0.01), with β describing changes in MNss score per range of motion degrees. Magni et al. (2018) reported a presence of neglect-like symptoms in hand osteoarthritis more often than in control healthy subjects (*p* < 0.001)(prevalence rate not reported); however, the magnitude of the difference was very low (median difference = 0.5 points; *personal communication*).

#### Body Ownership

The study of Martínez et al. (2018) is the only one to analyse body ownership through the RHI paradigm. They found that fibromyalgic patients were more susceptible to experience the illusion compared to healthy controls, scoring significantly (*p* < 0.05) higher both in proprioceptive drift sub-scale and in 4 out 5 items of the agency scale.

#### Perception of Surrounding Space

No studies assessed the personal and peri-personal space in MDRDs. Three studies investigated perception of extra-personal space using the Computerized Rod And Frame test (CRAF) in (Docherty et al., 2012; Treleaven and Takasaki, 2015) acute/recurrent and CNP (Grod and Diakow, 2002). During the CRAF subjects were asked to set a rod to the true vertical or horizontal: it provided a measure of the absolute error for the subjective perception of visual verticality/horizontality (SVV—SVO), and assessed the dependence on visual input for spatial orientation. Treleaven and Takasaki (2015) found a significant difference (*p* < 0.05) between patients with idiopathic CNP and both whiplash affected patients for SVV error (mean difference: 0.37°), and healthy controls (mean difference: 0.5°). This difference was referred to the Variable Error (VE), indicating the variability of the performance. Also the Root Mean Square Error (RMSE), representing the overall accuracy in achieving the true vertical, resulted significantly different (*p* = 0.01) between idiopathic neck pain patients and the other two groups (mean difference: 0.51° respect to healthy controls, and 0.58° respect to whiplash patients. By contrast, the absolute error and the direction of error were not able to detect between-groups differences (*p-*values respectively of 0.99 and 0.6). Unexpectedly, difference between patients with Whiplash Associated Disorders (WAD) and healthy controls was not significant in all error measurements evaluated, despite a higher level of disability in this sub-group respect to that with idiopathic neck pain. Docherty et al. (2012) (Docherty et al., 2012) assessed the (SVH error) in addition to the SVV error. Although both parameters were significantly different (*p* < 0.05) between CNP patients and healthy controls, they nevertheless fell into the range considered normal (<0.5°). A significant greater error (*p* < 0.001) in patients than in healthy controls was also found when using a variant with the frame tilted clockwise or counter-clockwise by 18°, but even in this case the median difference was small (<2°). Notably, 16% of patients with CNP scored higher than normal error in both the SVV and SVO, and they reported significantly (*p* < 0.016) higher disability at the Neck Disability Index respect to other patients with errors falling within the reference range of normality. The same small difference between groups (mean difference = 1.99°; *p* < 0.001) in SVV was found by (Grod and Diakow, 2002): in this case the experimental group was constituted by acute or recurrent neck pain, instead of CNP.

#### Qualitative Studies

From the interviews administered by Lauche et al. (2012a) emerged a distorted subjective perception of neck proportion (as if it was swollen) in CNP patients persisting even when patients were aware that this perception did not match actual appearance. The perception of enlarged body parts was found also by Valenzuela-Moguillansky (2013) in fibromyalgic patients through the administration of 'elicitation interviews', a methodology stemming from the phenomenological approach. They also reported other modification of the explicit SoP as changes in perceived heaviness, thickness and ownership: in stages of elevated level of pain, some patients described a paradoxical experience as if the painful body parts did not belong to them. Finally, they referred also the inability to localize painful body parts and an associated narrowing of the near space, as if it was shrunk while the body became larger.

### Intervention Studies

Four out twelve intervention studies were conducted in an experimental setting (Preston and Newport, 2011; Diers et al., 2013; Stanton et al., 2018; Nishigami et al., 2019), while the remaining eight were conducted in clinical settings (Barker et al., 2008; Wand et al., 2011, 2013a; Morone et al., 2012; Paolucci et al., 2012; Vetrano et al., 2013; Ryan et al., 2014; Louw et al., 2017). Supplementary Table 6 in Additional materials reports the methodology applied in each study, the clinical characteristics of patients, the outcome measures, the follow-up periods, and results. Only three studies monitored the treatment effect at follow-up periods (Wand et al., 2011; Morone et al., 2012; Vetrano et al., 2013). The study of Gilpin et al. (2015), despite adopting an intervention tool (the MIRAGE system), was excluded from the intervention studies because it lacked an end-point that measured pain and/or disability, thus it was assessed only under the evaluation studies for baseline data reported the perceived distortion of osteoarthritis patients' hand. One study (Diers et al., 2013), adopted the term 'upper back pain' with no details about the definition and boundaries of the functional diagnosis: first author declared to have enrolled patients with CNP (*personal communication)*.

#### Tactile Localization Training

Three studies (Barker et al., 2008; Wand et al., 2013a; Louw et al., 2017) adopted the concept of the somatic localization of touch (Longo et al., 2010), as a trainable perceptual ability. Wand et al. (2013a), in their cross-over randomized trial, administered a single session of acupuncture on the low back of two groups of 25 CLBP patients, asking in one group to localize where the needles have been inserted by depicting the point of needle insertion on a body chart. Pain was significantly (*p* = 0.008) less post-treatment (11-Numeric Rating Scale—NRS: −0.9; 95% CI = −0.3 to −1.5), regardless of the order of treatment administration (*p* = 0.182), but the magnitude of change was not clinically relevant. Pain reduction was higher (*p* = 0.011) for the group where the acupuncture was associated with the sensory discrimination training, however the effect size was not clinically relevant (11-Numeric Rating Scale: −0.8; 95% CI = −1.4 to −0.3). Louw et al. (2017) described a series of sixteen CLBP patients on which they administered a single, 5-min session of tactile localization, measuring pain intensity (11-NRS) and functionality (active lumbar flexion in centimetres). Patients were touched with the back of a pen on nine zones of the lower back in a random order; they were asked to localize the stimuli on a corresponding 9-block grid. Immediately after the treatment, pain decreased by 1.9 points (range: 0–6), and lumbar flexion increased of 4.8 centimetres (range: −1 to 21), in both cases over the clinical significance. Barker et al. (2008) compared the effect of a device (the FairMed) that is based on the principle of the localisation task, with a conventional TENS. The FairMed contains 16 vibrating points, controlled at distance and randomly activated. The subject has to localise where the vibrating point is acting and the device signals the correct responses through a visual and auditory feedback. Pain and disability improved significantly (*p* = 0.05), but without significant difference between the two devices.

#### Combined Therapy

Three studies (Morone et al., 2012; Paolucci et al., 2012; Vetrano et al., 2013) adopted the “SuPeR” (Surface for Perceptive Rehabilitation tool) and other two used a gradual perceptual re-training program (Wand et al., 2011; Ryan et al., 2014); all studies enrolled CLBP patients. The SuPeR treatment provided the adoption of postures and the execution of active exercises while lying supine on a table with a series of deformable latex cones of different hardness having the goal of stimulating the trunk skin surface: patients were asked to count and localize tactile stimuli, or to discriminate the hardness of cones. Morone et al. (2012) found an effectiveness in pain and disability levels reduction both for SuPeR treatment and Back School program respect to control group (medical and pharmacological assistance only) post-treatment and after 24 weeks (*p* < 0.001), but differences between two groups, despite statistically significant (*p* < 0.001), were never clinically relevant. The same trend was found also by Paolucci et al. (2012). Vetrano et al. (2013) studied a variant of the SuPeR treatment against the standard procedure described by Morone et al. (2012). The efficacy of the two proposed version of the SuPeR treatment were substantially equal (*p* > 0.05), and both procedures improves pain and disability respect to baseline values (*p* < 0.001), with variable clinical relevance (11-VAS range: −2 to −5.5, Oswestry Disability Index range: −14 to −21). Although an improvement in pain level and disability was globally reported for SuPeR approach, the effect size was variable and not always clinically significant at follow-up periods. Wand et al. (2011) described three cases of patients with CLBP treated with a mixed treatment comprising education, graded perceptual training (localization and graphesthesia tasks) and graded motor retraining for a minimum of 10 weeks: pain decreased at the end of treatment and after 1 month (2.9 and 3.9 on 11-NRS), as well as disability (5.2–9.6 on the Roland and Morris Disability Questionnaire-RMDQ), with statistical significance (*p* < 0.001). Finally, Ryan et al. (2014) adopted in a pilot-randomized trial controlled with a placebo group, the graded perceptual re-training protocol described by Wand et al. (2011), in adjunct to usual physiotherapy cares. Pain and disability improved after treatment in experimental group (−8 on 101-VAS and −1.6 on RMDQ), but without statistical significance (*p*>*0.05)*, respect to placebo group (−33.2 on 101-VAS and −4 on RMDQ; *p* < *0.0)*.

#### Experimental Setting

Among studies conducted in experimental settings, Diers et al. (2013) tested the visual manipulation of the neck in CNP patients and healthy subjects Authors provided visual feedback, of the neck (neutral, enlarged or downscaled visual appearance) and of a neutral body part (hand dorsum), during pressure and electrical pain stimulation of the trapezius muscle. They found that all visual conditions of the neck (*p* < *0.001)* but not of the neutral hand (*p*>*0.05)*, reduced the perceived intensity of applied acute painful stimuli, both in patients and in controls, but changes were all under the clinical significance. Preston and Newport (2011) adopted the MIRAGE-multisensory illusion system, in patients with hand osteoarthritis, while Stanton et al. (2018) applied it in knee osteoarthritis patients. The MIRAGE system involved the visuo-tactile manipulation of a body part, inducing their stretching or a shrinking visual appearance, in addition to a tactile stimulation applied by the examiner that may be directed in a congruent or incongruent modality (tactile stimulation in the same direction of the visual illusion (e.g., in stretching direction, or in the opposite way). Both studies reported an analgesic effect, with a reduction in pain varying between 45 and 50% respect to the baseline in the study of Preston and Newport (2011), and 25% in the study of Stanton et al. (2018). In this last study it was also found that repetition of the illusion, better than prolonging the exposure, produced additional pain relief (40% respect to the baseline). Finally, Nishigami et al. (2019) adapted and preliminary tested the MIRAGE system in two CLBP patients without the adjunct of the tactile stimulation to the visual manipulation. In this pilot-study, the authors proposed a visual manipulation of the trunk modifying its muscular appearance, based on the common maladaptive beliefs of CLBP subjects about robustness and perceived vulnerability of their back. This kind of “cognitive” illusion seems to reduce pain only in the subject A, having higher level of body perception dysfunction (FreBAQ score: 29/36), catastrophization (Pain Catastrophizing Scale score—PCS: 50/52) and maladaptive beliefs (Back Beliefs Questionnaire score—BBQ: 67/45), respect to the subject B with lower scoring on these outcomes (FreBAQ: 0/36; BBQ: 39/45; PCS: 8/52).

#### Qualitative Studies

The study of Lauche et al. (2012a) is a qualitative investigation embedded in a RCT on the effect of the cupping therapy in CNP patients, compared to similar patients on a waiting list to receive treatment (Lauche et al., 2012b). From the interviews emerged a subjectively referred reduction in neck size (smaller), as consequences of pain relief. The Body Image Drawing (BID) appeared changed in both groups, but drawings were more complete and lines more symmetric, in the cupping therapy group.

### Methodological Considerations

**Figure 7:**
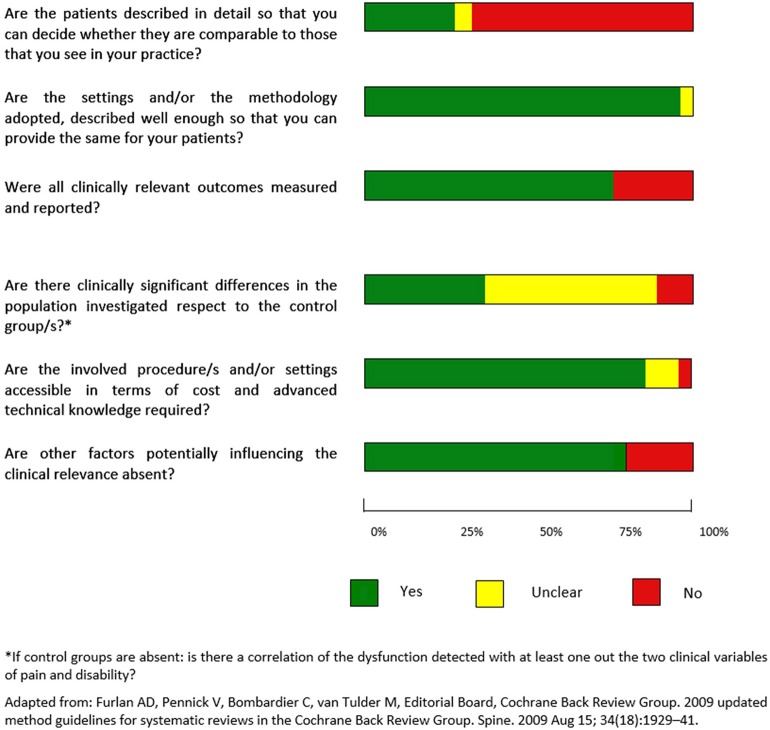
Summary of the evaluation for clinical relevance of included assessment studies.

**Figure 8:**
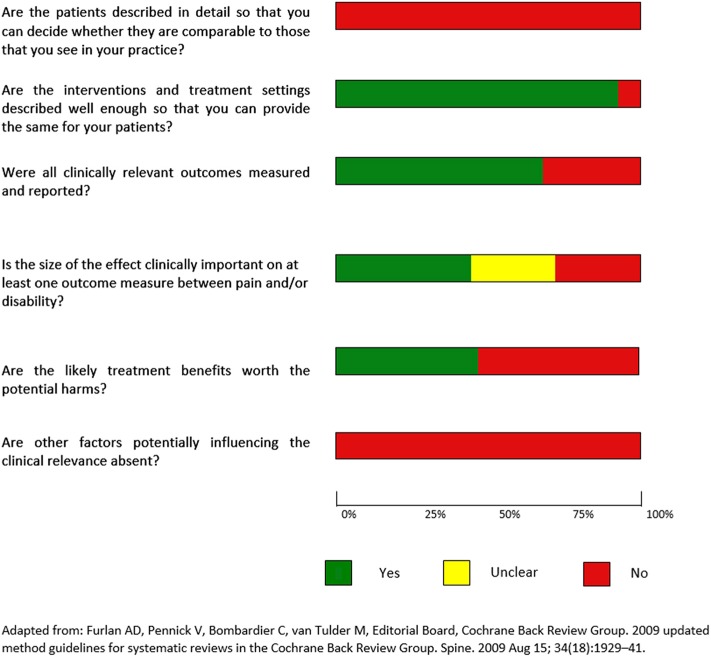
Summary of the evaluation for clinical relevance of included treatment studies.

Only 5 out of the 38 included studies (Barker et al., 2008; Wand et al., 2013a, 2016; Adamczyk et al., 2018b; Nishigami et al., 2018) provided a-prori calculations of the sample size, thus their results cannot be generalized to larger population. A rich variety of research designs was adopted: 9 out 38 studies were case studies, case series, pilot trials or preliminary investigations (aggregated they represent 24% of all included studies) (Moseley, 2008; Preston and Newport, 2011; Wand et al., 2011; Louw et al., 2017; Moreira et al., 2017; Adamczyk et al., 2018a,b; Stanton et al., 2018; Nishigami et al., 2019) This strongly limit the comparison of findings between studies. These nine studies had also small sample sizes ranging between 2 and 20 subjects in nine studies (24% of cases), limiting the generalizability of results. Inclusion and exclusion criteria have been poorly documented in 29/38 studies (76%) (Figures 7, 8): the selection of the target population may have been not adequately performed, potentially biasing the validity of results. Sixteen percent of all included studies were conducted in an experimental setting (Grod and Diakow, 2002; Wand et al., 2010; Docherty et al., 2012; Gilpin et al., 2015; Treleaven and Takasaki, 2015; Martínez et al., 2018), limiting the applicability of methodologies proposed in clinical practice.

#### Assessment Studies

Groups of control subjects were not tested in six studies (Wand et al., 2013a, 2016; Hirakawa et al., 2014; Adamczyk et al., 2018a,b; Nishigami et al., 2018), and two case-control investigations had no healthy subjects comparisons (Moseley, 2008; Beales et al., 2016): this issue represents a major limitation of these works, potentially limiting the validity of their findings. Although we can presume that explicit SoP of body parts should be normal in unaffected people (Longo and Haggard, 2012), a degree of distortion for implicit SoP has been found even in healthy people (Fuentes et al., 2013; Longo, 2017). For this reason the absence, or small size of healthy control groups weighs more on the validity of the studies investigated the implicit SoP (Wand et al., 2010, 2013b; Adamczyk et al., 2018a,b) respect to those investigated the explicit SoP.

The FreBAQ and FreKAQ questionnaires were adapted from the NLSQ of Galer and Jensen (1999) and Frettlöh et al. (2006) validated in CRPS patients. Studies adopting these outcomes represented the majority of all the assessment studies (40%). It should be noted that questionnaires measuring explicit SoP are not validated against standard measures of reference. Of course, the criterion-validity of scales measuring this construct remain unknown and the contribution of the implicit body model and of the explicit cognitive representation are difficult to disentangle. Nevertheless, it must be considered that FreBAQ and FreKAQ involve two items asking for the explicit size and shape perception of body parts (items 6–9): they could be validated adopting recently proposed objective measure for the explicit SoP itself and for the body model (Longo and Haggard, 2010; Fuentes et al., 2013; Spitoni et al., 2015; Adamczyk et al., 2018b), accounting for the implicit perception of metric sizes of body parts. Moreover, item 5 of FreBAQ/FreKAQ asked about the explicit perceived location of body parts in space: it may be validated adopting the objective methodologies proposed by Longo and Haggard (2010) for the implicit position sense. Finally, other items investigated the body ownership (item 1) and agency (items 3 and 4), two constructs that have been extensively studied through the RHI paradigm and relative psychometric measures (Longo et al., 2008a).

Notably, we found only one study investigated the responsiveness of assessment tools respect to changes in clinical status (Lauche et al., 2012a). However, the qualitative nature of this investigation provide us only indicative data.

#### Treatment Studies

As highlighted in Figure 8, some issues threatened the clinical relevance of included studies. Four studies recruited not adequate control groups: for e.g., the study of Barker et al. (2008) compared the FairMed device with the TENS in CLBP patients. However, Cochrane Reviews (Khadilkar et al., 2005, 2008) found limited evidence for the use of TENS in CLBP treatment, and the international guidelines recommend to use a mixed-type of intervention in patients with this kind of disorder, composed by physiotherapy, exercises and psychological treatments (National Guideline Centre, 2016). Thus, the sole use of the TENS cannot be considered as the gold standard treatment for LBP, and FairMed device should be tested against a placebo-control group or with another more effective treatment. The same issue involved also the study of Morone et al. (2012), Paolucci et al. (2012), and Vetrano et al. (2013) in which the experimental SuPeR treatment approach for CLBP was compared to a group of patients who performed back school exercises, and to another group who performed a variant of the standard SuPeR treatment. The qualitative study of Lauche et al. (2012a) was embedded in a RCT (Lauche et al., 2012b) with a waiting-list control group and therefore it lacks of a comparison with other usual cares or placebo interventions.

Only two studies (Stanton et al., 2018; Nishigami et al., 2019) evaluated at the baseline the presence of perceptual dysfunction thus, a large part of the treatments provided perceptual tasks aimed to reduce pain and/or disability without taking in consideration the potential relationship between SoP and pain perception. This methodological issue represents a major limitation of all intervention studies. Failure to detect possible sub-groups, as those found in some assessment studies for explicit SoP (Mibu et al., 2015; Nishigami et al., 2015; Moreira et al., 2017), may have limited the effectiveness of therapeutic procedures because authors may not have taken into account that different kind of misperception could produce variable results to the same treatment. Currently, therefore, it is not possible to draw any conclusions regarding their potential clinical value.

The adverse events were not reported across all included studies (Figure 8): although the majority of studies adopted non-invasive procedures we lack evidence about the occurrence of side effects, especially for two studies using invasive procedures (acupuncture and cupping therapy) (Lauche et al., 2012a; Wand et al., 2013a). Moreover, authors of the studies where bodily illusions were administered through mediated-reality systems (Preston and Newport, 2011; Diers et al., 2013; Stanton et al., 2018; Nishigami et al., 2019), despite the minimal invasiveness of the procedures, did not report information for the tolerability of the equipment and of the illusions itself. In eight studies, the therapeutic procedures required dedicated technological (Barker et al., 2008; Preston and Newport, 2011; Diers et al., 2013; Stanton et al., 2018; Nishigami et al., 2019) or homebuilt equipment (Morone et al., 2012; Paolucci et al., 2012; Vetrano et al., 2013): even if the materials assembly procedure is well described (Morone et al., 2012; Paolucci et al., 2012; Vetrano et al., 2013), costs were not reported, potentially limiting the clinical applicability of these therapeutic tools. Sixty-three percent of patients in the study of Barker et al. (2008) reported faults of the FairMed device during the experiments, a concern that may have limited the efficacy of the treatment tested.

Except for Morone et al. (2012) and Vetrano et al. (2013), almost all studies provided follow-up periods no longer than 4 weeks, thus limiting the possibility to assess long terms effect for treatments proposed. From a conceptual and terminological point of view, in some studies authors reported using sensory-based interventions (Barker et al., 2008; Morone et al., 2012; Ryan et al., 2014) where, instead, perceptual-based therapeutic strategies were tested. This distinction is not trivial because primary sensory processing is different respect to higher-order mechanisms underlying SoP (Longo and Haggard, 2010; Hillier et al., 2015; Mancini et al., 2015; Spitoni et al., 2015). This terminological misuse may hide an important conceptual issue: some studies may have been conceived and designed with the goal to ameliorate primary *somatosensations* (tactile acuity, proprioception, etc.), rather than SoP, and the results found may have been consequently biased by these conceptual and practical mismatches.

Finally, calculation of effect sizes' confidence intervals were not possible in four studies (Preston and Newport, 2011; Morone et al., 2012; Vetrano et al., 2013; Louw et al., 2017) due to lack of relevant information: this reporting bias may have compromised the accuracy of results.

## Discussion

To “live” our own body constitutes a fascinating and complex experience because the body represents a unique multisensory object. The body experience is not direct, as well as bodily illusions and pain perception, two perceptual experiences that illustrate the complexity of mental organizations. Rather, it is filtered by a numbers of factors such as somatosensory inputs, perceptual information and body memory (Riva, 2018). Therefore, to study perceptual disorders in painful conditions represents an opportunity to explore the mechanisms underlying how our brain generates the experience of one's body. The aim of this review was to provide a comprehensive map about the literature published on perceptual disorders in painful MDRDs, with the main goal to identify gaps in current knowledge and to obtain useful information for the future research agenda. Our findings should be interpreted considering the large methodological variety of included studies.

The amount of literature found (37 articles) attests that, since the first investigation of Moseley (2008), overall these topics have been received some attention during the last decade. Specific sub-groups of MDRSs have been investigated more extensively, as in the case of spinal pain. CLBP, CNP and Pelvic Pain, taken together, represent about 80% of all the included studies. At the same time, it is noticeable how some others clinical conditions such as the rheumatic diseases remained with little or sparse interest. For example, rheumatoid arthritis was not investigated, and only two studies (5%) investigated fibromyalgia (Valenzuela-Moguillansky, 2013; Martínez et al., 2018). Another poorly explored area is pain in upper and lower limbs (15% of included studies), among which osteoarthritis was the only studied condition (Preston and Newport, 2011; Hirakawa et al., 2014; Gilpin et al., 2015; Nishigami et al., 2017; Magni et al., 2018; Stanton et al., 2018).

### Summary of Evidence and Clinical Interpretation

#### Assessment Studies

We found a wide and heterogeneous literature published in the field of MDRDs about SoP, SpP and BO. It predominantly concerned about the assessment (66% of included studies, 25/38), respect to the intervention strategies (34%, 13/38). Some preliminary evidence of distorted body experience in MDRDs emerged, mainly in the area of the explicit SoP for spinal pain assessed through the BID task (Moseley, 2008; Lauche et al., 2012a; Mibu et al., 2015; Nishigami et al., 2015; Moreira et al., 2017), questionnaires or visual estimation tasks (Gilpin et al., 2015). These preliminary findings are in line with evidence found in CRPS (Galer and Jensen, 1999; Förderreuther et al., 2004; Frettlöh et al., 2006), although with apparent less magnitude and frequency. In fact, NLS were reported in 54.4–90.2% of CRPS patients respect to 19–36%. found in MDRDs (Hirakawa et al., 2014). Notably, the results obtained through the BID are difficult to interpret and compare to each other due to the qualitative nature of this task: the assessment of the altered explicit SoP is left to the clinician's subjective judgment, potentially leaving a large margin of error in interpreting the results of the test. Moreover, both the BID task and questionnaires like FreBAQ/FreKAQ and NLSQ, involved a self-description and depiction of own's body parts in which are involved both perceptive and cognitive/affective contributions that are not easily separable. For this reason, these tasks should be considered as a complex and multidimensional way to assess explicit body experience. On the other hand, although some promising assessment methodologies have been proposed, a substantial gap in knowledge exists in the area of the implicit mechanisms guiding perceptual abilities, like the estimation of body parts' size and its location in space. The absence of studies that investigate the sub-domain of the implicit SoP may be interpreted as a lack of appropriate tools in MDRDs able to investigate this construct, or as a sparse knowledge about the distinction between implicit and explicit mechanisms underlying SoP. This may be not surprising if we consider that: a) this area of investigation is peculiar of the neuropsychology rather than rehabilitation sciences dealing with MDRDs; and (a) the comprehension about neural and operational mechanisms of body experience has grown only in the last few years (Longo et al., 2008b, 2015; Longo, 2015; Gallace and Bellan, 2018).

We found only three studies investigating the implicit SoP in MDRDs (Wand et al., 2013b; Adamczyk et al., 2018a,b), of which one is a preliminary validation study and another is a case-study. Therefore, it appears that implicit SoP in MDRDs has received little attention, as well as in CRPS, where only sparse studies have been investigated this sub-domain of body perception (Lewis et al., 2010b; Reiswich et al., 2012). Despite this, some methodologies proposed in preliminary studies showed good psychometric values, as in the case of the 2-PET (Adamczyk et al., 2018b), and are easy to be implemented both in clinical practice (Wand et al., 2013b; Adamczyk et al., 2018a) and in future research studies.

Variable results were found for the association of SoP disorders with pain intensity, duration and disability. Higher and stronger associations were found in studies that examined the explicit SoP (mainly those adopting the FreBAQ and FreKAQ) compared to other methodologies, while conflicting results were found for studies assessing the implicit SoP. However, in this last case, the small number of studies (Wand et al., 2013b; Adamczyk et al., 2018a,b) and of subjects recruited may have influenced the findings. Overall, it is not possible to draw conclusions about causation due to the lack of cohort studies. In fact only one study had a longitudinal research design (Hirakawa et al., 2014): authors found a decreased level of perceptual dysfunctions 6 weeks post-knee arthroplasty but standard deviation values were high, indicating large variation among patients. Thus, it is unclear whether SoP dysfunctions are a consequence of persistent painful states, potential contributing factors or an epiphenomenon of pain. One proposed hypothesis has been reported in pregnancy-related pelvic pain in which body changes precede the onset of pain: in this case, anatomical variations in body sizes may have caused pain-related thoughts and fear of movements, generating maladaptive behaviours and altered body perceptions (Beales et al., 2016). In other conditions different than pregnancy-related ones, this combination of factors may explain misperceptions occurring only in body parts potentially affected by increment of size (e.g., axial joints, interphalangeal and metacarpal joints) where SoP distortions could occur subsequently to swelling phases (McCabe et al., 2004), but they may be not able to explain misperceptions affecting the spine.

##### Sub-groups detection

In some studies (Mibu et al., 2015; Nishigami et al., 2015; Moreira et al., 2017) seem to emerge sub-groups of patients with different features for the explicit SoP (normal, augmented and shrunken), although the association of each group with higher disability levels, or pain duration and intensity remains unclear. Similarly, for implicit SoP, Adamczyk et al. (2018a) presented a two-case report in which one patients showed an overestimation of the painful low-back side compared to non-painful locations in 2-PET (range: 45–206%) and an opposite trend in the second subject (underestimation ranged between 12 and 22%). However, it cannot be established if two or more different sub-groups emerged also for the implicit domain of SoP because this is the only study that found this apparent trend. The same authors, in another study with larger sample of CLBP patients (Adamczyk et al., 2018b), were not able to find the same sub-groups split found in the first double-case study. They also identified an overall underestimation of both sides of the spine in 2PET, challenging the relationship between pain location and body perception distortion.

##### Body ownership

As a part of our body experience, we have to consider that mental representations of own body include the concept of shape and contours perception of body parts (James, 1890) and the boundaries between them and the external space. The plasticity of this kind of body *representation* has been extensively studied through the RHI paradigm (Botvinick and Cohen, 1998). However, we found only one study investigating the response to the RHI in MDRDs (Martínez et al., 2018). These authors found that fibromyalgic patients were more prone to experience the illusion than controls. The capacity to localize and confine body sensations within the corporeal boundaries requires an intact *somatorepresentation*: a misperception in which a rubber hand 'taking the place' of own's real hand (body representation instability), could indicate a dysfunction in multisensory integration underlying SoP function, but it is not clear the relationship with clinical relevant variables, and thus the potential role played in pathophysiology of chronic pain. To the best of our knowledge, only one study was published assessing the RHI in CRPS patients (Reinersmann et al., 2013). Authors found preserved multisensory integration despite the presence of neglect-like symptoms, indicating a possible dissociation between the mechanisms involved in BO and explicit SoP. Noteworthy, it seems to appear a potential analogy between fibromyalgia and eating disorders: both conditions seem to have a more instable BO respect to healthy controls (Mussap and Salton, 2006; Eshkevari et al., 2012; Keizer et al., 2014) and are joined by augmented vigilance to internal body signals. In addition, they seemed to show dissatisfaction regarding some body parts, those more painful in fibromyalgic subjects and emotional-sensitive ones in anorectic and bulimic patients. Body dissatisfaction is thought to be caused by the discrepancy between an ideal body model and the current self-perception (Strauman et al., 1991; Vartanian, 2012). Despite the causation relationship is still unclear, it was found a correlation between negative body affective perception and pain severity in fibromyalgic patients (Akkaya et al., 2012). In our opinion, in order to avoid ineffective and limited approaches, as already found in eating disorders (Eshkevari et al., 2014), it should not be neglected the presence of such negative body-cognition appraisal also in fibromyalgic patients. The variable contribution to body experience of cognitive, affective and perceptual mechanisms should be considered in further studies, as already proposed for eating disorders treatment (Riva, 2011; Keizer et al., 2014; Serino et al., 2016a,b).

##### Space perception

Evidence emerged across included studies seemed to highlight the absence of SpP dysfunctions, at least for the extra-personal space measured through the CRAF test in a sample of patients with CNP and WAD: errors in SSV and SVO were under the limit of normality or very modest, and were not correlated with disability. The lack of studies conducted in disorders different than CNP and WAD makes it difficult to extent these findings to others MDRDs, or to compare these results with those found in CRPS (Sumitani et al., 2007a,b; Uematsu et al., 2009; Reinersmann et al., 2012; Christophe et al., 2016).

#### Treatment Studies

The majority of published intervention studies were preliminary pilot-tests, case studies and case series, or were conducted in experimental settings. For these reasons, evidence emerged about the intervention strategies proposed are very limited. Moreover, it is difficult to estimate the relative effectiveness of each single therapeutic component for studies adopting concomitant multiple approaches (Wand et al., 2011; Morone et al., 2012; Paolucci et al., 2012; Vetrano et al., 2013; Ryan et al., 2014). Overall, intervention studies suffered the absence of preliminary assessment for dysfunctions of SoP and BO at the baseline: this issue may have limited the effectiveness of the treatments because they were not appropriately focused on specific sub-groups of patients. In fact, as shown by assessment studies, some patients with MDRDs seem to present explicit or implicit SoP disorders respect to others (Mibu et al., 2015; Nishigami et al., 2015; Moreira et al., 2017; Adamczyk et al., 2018a).

Although they must be considered within the limits of their low evidence value, case studies and preliminary investigations showed promising results of dedicated interventions (Stanton et al., 2018; Nishigami et al., 2019) addressing specific kind of SoP disorders at the baseline, as found in CRPS (Lewis et al., 2019).

Despite the presence of major methodological limitations, some therapeutic strategies could be of potential clinical value, especially in light of the brief duration and frequency of administration (Wand et al., 2013a; Louw et al., 2017).

### Clinical Implications

Currently, evidence is fragmented and insufficient to guide precise assessment and intervention actions in routinely clinical practice. Nevertheless, the majority of treatment methodologies and assessment tools described in this review represent simple, safe and inexpensive procedures, feasible for the use in daily clinical practice. Some of the therapeutic approaches proposed seem to improve movement and pain without performing any physical action (Louw et al., 2017; Nishigami et al., 2019). For this reason, they may be promising strategies to use in patients with elevated pain levels and movement restrictions, as in person having high level of fear-avoidance behaviours for movements and maladaptive beliefs, especially in early rehabilitation phases (Louw et al., 2017).

Patients affected by these kind of perception disturbances (as documented in CRPS) may be reluctant, if not directly questioned (Galer and Jensen, 1999; Lewis et al., 2010a), to talk with health care providers or within the family context (Galer and Jensen, 1999), due to the bizarre features that make them appear as having some form of psychological/psychiatric disturbance (Förderreuther et al., 2004), or fearful of not being believed (Lewis et al., 2007). The perception of body contours and ownership is usually taken for granted, but in circumstances in which derangements appear between what is perceived and what is real, both pain and stressful response may potentially increase as consequence to these conditions, together with fear-avoidant behaviour. For patients, not being able to rely on information coming from their bodies and experiencing such bodily illusions can be detrimental for quality of life, social interactions and, overall, for mental health (Lewis et al., 2007; Longo, 2015). For these reasons, despite the limited diagnostic capacity of the tools now available, we believe it is important that clinicians start to approach (Geri et al., 2019) and validate this kind of unpleasant experience. For instance, clinicians could tell patients that their clinical descriptions resemble the very common situation of receiving an injection from the dentist and thereby perceiving one's lips and cheeks as uncommonly swollen and distorted, despite one's awareness that they maintain their normal size. Moving from the preliminary findings of this review, clinicians should consider the role of distorted SoP, starting from directly asking patients about their body experience, or through the administration of easy and inexpensive qualitative and quantitative tools, as the BID and the 2-PET.

### Recommendation for Future Research

Despite the range of methodological issues that limit the validity of the evidence we have discussed, some of the proposed assessment methodologies and therapeutic strategies, could represent useful starting points for further research.

Considering the complexity of the body experience phenomenon, future studies should consider the concomitant assessment of different domains of bodily experience (explicit and implicit SoP, BO, and SpP), in parallel to the clinical variables commonly used for clinical studies, as some authors have started to do with CRPS patients (Lewis et al., 2019). Intervention studies should determine the response of particular sub-groups of patients, for e.g., those with enlarged or diminished body perception (Mibu et al., 2015; Moreira et al., 2017; Adamczyk et al., 2018a), to dedicated perceptual training interventions (Lewis et al., 2019). Moreover, the preliminary detection of perceptual impairments and the potential identification of particular sub-groups in clinical studies may help to identify individuals who could potentially benefit from dedicated treatments, or sub-groups that may be resistant to usual cares.

Future studies may implement new advanced technologies for clinical purposes. For e.g., diagnostic studies may implement more accurate new digital tools (Turton et al., 2013), aimed at overcoming the excessive subjectivity of the clinicians in the assessment of BID, but preserving at the same time the subjectivity of patients in expressing their own's SoP. Virtual and augmented reality represent probably the new frontier for the study of body representation finalized at therapeutic clinical use in body perception dysfunctions.

It may be interesting to explore also the neural correlates of body experience disorders through the adoption of neuroimaging methods, such as functional magnetic resonance imaging (fMRI), during perceptual tasks execution, without limiting the investigation to the functionality of the primary sensory area, as primarily performed in MDRDs field since now.

The available studies on assessment and treatment described here need to be replicated in larger and higher-methodological quality studies with appropriate control groups, in order to confirm preliminary results emerged, and to determine whether perceptual disorders represent clinical consistent findings. At the same time, we encourage the production of diagnostic case studies/case series and the publication of preliminary validation studies aimed to describe new assessment and treatment methodology in MDRDs as already made in CRPS (Sumitani et al., 2007a; Uematsu et al., 2009; Christophe et al., 2016; Solcà et al., 2018). We think that these two preliminary steps may be useful starting points before large scale data collection, as in the case of Adamczyk et al. (2018a,b).

The implicit and explicit body experience represents certainly a complex construct to define and investigate. Despite the absence of recognized gold-standard procedures to validate perceptual dysfunctions, it's noteworthy that other psychophysical tests have been proposed (Longo and Haggard, 2010; Fuentes et al., 2013) and may be implemented in MDRDs.

Finally, both the FreBAQ/FreKAQ and the NLSQ items were not directly derived from patients' self-experience dedicated studies, as those represented by the qualitative research. Moreover, these questionnaires were borrowed and adapted from studies on CRPS patients, rather than from studies investigating directly MDRDs patients. For these reasons, qualitative interviews-based studies may represent a useful and appropriate methodological approach to obtain relevant themes to adopt for the implementation of questionnaire items directly based on patients' “first-person” perspective (Lewis et al., 2007; Valenzuela-Moguillansky, 2013). We must consider that SoP, SpP and BO, are essentially subjective phenomena. Therefore, we cannot achieve comprehensive and deeper knowledge on this personal experience without a direct involvement of patients with dysfunctional body experiences.

### Strengths and Limitations

Our research team was multidisciplinary and multiprofessional in its composition aimed at limiting potential conflicting interpretations of terminology and concepts investigated (Anderson et al., 2008). Despite the comprehensive nature of this review and the amount of sources scanned, it is possible that the limitation of our search only to English language studies may have influenced the nature of the evidence found. In order to limit this potential publication bias, we have reported in additional materials (Supplementary Table 7) all potential eligible studies found in other languages, having at least the abstract in English. The presence of only one author for data extraction may have been a potential source of bias: to overcome this limitation we provide a secondary data check by a second reviewer. We limited our investigation to patients >16 years old, however potentially interesting findings and assessment strategies may be found in the research area of the idiopathic scoliosis (Picelli et al., 2016; Paolucci et al., 2017). The extensive heterogeneity of included studies prevents to draw robust conclusions for clinical practice. Nevertheless, the inclusion of quantitative, qualitative and mixed-method studies at this first literature mapping stage allowed to consider the different aspects involved in the complex phenomenon of the body experience. Finally, the theme of SoP is broad and this review cannot be considered exhaustive. We excluded studies investigating the perception of own's body under dynamic condition (Valenzuela-Moguillansky et al., 2017) and the related therapeutic strategies proposed (Horwitz et al., 2003, 2004; Wand et al., 2012), because they are not considered in the theoretical framework of Longo et al. (Longo et al., 2010; Longo, 2016) that we have adopted as reference. Moreover, we did not consider other kinds of perceptual information as those related to the perceived stiffness (Haigh et al., 2003; Stanton et al., 2017).

## Conclusions

Alterations of the implicit and explicit body experience have been preliminary found through this literature review. Despite the unclarity about the association or causation with chronic pain in MDRDs, perceptual dysfunctions could be reasonably considered as having a potential impact on clinical outcomes. If confirmed in future methodological robust studies, they may be potentially considered as one of the dimensions involved in clinical presentations of MDRDs, on a par with pain perception, functional limitations and restrictions in participation. Since an effective treatment depends on an effective diagnostic procedure, before conducting new treatment studies, future research should prioritize the objectification of perceptual dysfunctions subjectively referred by patients or reported through qualitative methods.

Some important questions remain open to be addressed: (a) explicit and implicit dysfunctions found in CLBP and CNP may constitute a cause, a consequence or an epiphenomenon respect to pain perception?; (b) the sub-groups highlighted in CLBP and CNP are consistently present even in other kind of MDRDs and also in the implicit domain of SoP; and (c) considering that in healthy subjects, a degree of distorted implicit somatoperception has been found in recent neuropsychological studies with respect to an intact explicit mechanism (Longo, 2017), we have to expect a further deterioration of the implicit somatoperception in those patients found to have yet an altered conscious perception of their own's body?

In order to answer to these questions, we conclude by suggesting three future research lines:

- Longitudinal studies providing pain and multiple body perception outcome measures, along an enough large period of observation, may constitute an appropriate answer to the first point;
- Implicit and explicit methodology of assessment should be administered in parallel to patients in order to test the potential divergences between conscious and unconscious mechanisms of body perception within and between MDRDs: this suggestion answer both to the second and the third critical points highlighted;
- Moreover, the adoption of case-cohort studies with control groups of healthy subjects, further answer to the third point.

Overall, in consideration of the amount of literature already published since now we sustain and propose that the theoretical concept to split between implicit and explicit mechanisms of SoP may constitute an important starting point for future research agenda.

As suggested by extensive literature in neuropsychological field, pain perception appears to interact with a range of factors, among which implicit and explicit SoP, SpP and BO. For this reason, for future research agenda we encourage researchers to combine experimental lines on body experience with those studying chronic pain in MDRDs.

In consideration of findings emerged and the quality of studies found, at the state of art, the conduction of a future systematic review is appropriate only to synthesize the psychometric properties of the FreBAQ.

## Author Contributions

AV contributed to conception of the study, managed the database, extracted data from studies, and wrote the first draft of the manuscript. AV and DR contributed to the design and performed the first search strategy. DR and AG performed peer-review of electronic search strategies. EC, DL, and MP performed calibration phases, screening, and selection steps. DP and AV performed pilot-trials of data extraction. DP performed crosschecking of data extraction. DP and GR performed data analysis. GR, ML, and MT performed data interpretation. AG, GR, ML, and MT wrote sections of the manuscript. All authors contributed to manuscript revisions, read and approved the submitted version.

### Conflict of Interest

The authors declare that the research was conducted in the absence of any commercial or financial relationships that could be construed as a potential conflict of interest.

## Footnotes

^1^https://www.cadth.ca/resources/finding-evidence/grey-matters

## Abbreviations

2-PET: 2-Point Estimation Task
BO: Body Ownership
BID: Body Image Drawing
CLBP: Chronic Low Back Pain
CNP: Chronic Neck Pain
CRAF: Computerized Rod And Frame test
CRPS: Complex Regional Pain Syndrome
FreBAQ: Fremantle Back Awareness Questionnaire
FreKAQ: Fremantle Knee Awareness Questionnaire
MDRDs: Musculoskeletal Disorders and Rheumatic Diseases
MNss: Motor-Neglect sub-scale
NLSQ: Neglect-Like Symptoms Questionnaire
PTP: Point-to-Point
RHI: Rubber Hand Illusion
SoP: Somatoperception
SpP: Space Perception
SVV: Subjective Visual Verticality
SVH: Subjective Visual Horizontality
WAD: Whiplash Associated Disorders.
