# Supplementary material for: Explicit and Implicit Own's Body and Space Perception in Painful Musculoskeletal Disorders and Rheumatic Diseases: A Systematic Scoping Review
Source: Front Hum Neurosci. 2020 Apr 9;14:83. doi: 10.3389/fnhum.2020.00083 (PMC7161420; doi:10.3389/fnhum.2020.00083)
Supplement: Supplementary file 1 [file Table_1.DOCX]

Appendix

[Additional File 1: Supplementary Table S1 2](#_Toc21240362)

[Preferred Reporting Items for Systematic reviews and Meta-Analyses extension for Scoping 2](#_Toc21240363)

[Reviews (PRISMA-ScR) Checklist: 2](#_Toc21240364)

[Additional File 2. Search Strategy 5](#_Toc21240365)

[Medline (PubMed) 5](#_Toc21240366)

[User Query for PubMed: 7](#_Toc21240367)

[Cochrane Library (Cochrane Database for Systematic Reviews – Database of Abstracts of Reviews of Effect – Cochrane Central Register of Controlled Trials) 9](#_Toc21240369)

[PEDro (Physiotherapy Evidence Database) 11](#_Toc21240371)

[The Campbell Collaboration 13](#_Toc21240373)

[SCOPUS 15](#_Toc21240375)

[PsychINFO (EBSCOhost) 17](#_Toc21240377)

[Web of Science 20](#_Toc21240379)

[TRIP Database 23](#_Toc21240381)

[ProQuest Central 24](#_Toc21240383)

[PQDT Open 25](#_Toc21240385)

[ClinicalTrials.gov 26](#_Toc21240387)

[Google Scholar 27](#_Toc21240389)

[Google 28](#_Toc21240391)

[Electronic Journal Hand Searching: https://www.scimagojr.com/ 29](#_Toc21240393)

[Additional File 3 – Supplementary Table S2 - Questions for the determination of clinical relevance of included studies 30](#_Toc21240394)

[Additional File 4 – Supplementary Table S3 - Evaluation of clinical relevance for assessment studies 31](#_Toc21240395)

[Additional File 5 – Supplementary Table S4 - Evaluation of clinical relevance for treatment studies 34](#_Toc21240396)

[Additional File 6 – Supplementary Table S5: Detailed features of included assessment studies 36](#_Toc21240397)

[Additional File 7 – Supplementary Table S6: Detailed features of included intervention studies 59](#_Toc21240398)

[Additional File 8 - Supplementary Table S7: Excluded studies with reasons 74](#_Toc21240399)

# Additional File 1: Supplementary Table S1

## **Preferred Reporting Items for Systematic reviews and Meta-Analyses extension for Scoping** **Reviews (PRISMA-ScR) Checklist:**

| **SECTION** | **ITEM** | **PRISMA-ScR CHECKLIST ITEM** | **REPORTED ON PAGE #** |
| --- | --- | --- | --- |
| **TITLE** | | | |
| Title | 1 | Identify the report as a scoping review. | 1 |
| **ABSTRACT** | | | |
| Structured summary | 2 | Provide a structured summary that includes (as applicable): background, objectives, eligibility criteria, sources of evidence, charting methods, results, and conclusions that relate to the review questions and objectives. | 1-2 |
| **INTRODUCTION** | | | |
| Rationale | 3 | Describe the rationale for the review in the context of what is already known. Explain why the review questions/objectives lend themselves to a scoping review approach. | 3-4 |
| Objectives | 4 | Provide an explicit statement of the questions and objectives being addressed with reference to their key elements (e.g., population or participants, concepts, and context) or other relevant key elements used to conceptualize the review questions and/or objectives. | 4 |
| **METHODS** | | | |
| Protocol and registration | 5 | Indicate whether a review protocol exists; state if and where it can be accessed (e.g., a Web address); and if available, provide registration information, including the registration number. | 4 |
| Eligibility criteria | 6 | Specify characteristics of the sources of evidence used as eligibility criteria (e.g., years considered, language, and publication status), and provide a rationale. | 4-5, 37 |
| Information sources* | 7 | Describe all information sources in the search (e.g., databases with dates of coverage and contact with authors to identify additional sources), as well as the date the most recent search was executed. | 5, Additional File 2 |
| Search | 8 | Present the full electronic search strategy for at least 1 database, including any limits used, such that it could be repeated. | Additional file 2 |
| Selection of sources of evidence† | 9 | State the process for selecting sources of evidence (i.e., screening and eligibility) included in the scoping review. | 5 |
| Data charting process‡ | 10 | Describe the methods of charting data from the included sources of evidence (e.g., calibrated forms or forms that have been tested by the team before their use, and whether data charting was done independently or in duplicate) and any processes for obtaining and confirming data from investigators. | 6 |
| Data items | 11 | List and define all variables for which data were sought and any assumptions and simplifications made. | 6 |
| Critical appraisal of individual sources of evidence§ | 12 | If done, provide a rationale for conducting a critical appraisal of included sources of evidence; describe the methods used and how this information was used in any data synthesis (if appropriate). | n.a. |
| Synthesis of results | 13 | Describe the methods of handling and summarizing the data that were charted. | 6-7 |
| **RESULTS** | | | |
| Selection of sources of evidence | 14 | Give numbers of sources of evidence screened, assessed for eligibility, and included in the review, with reasons for exclusions at each stage, ideally using a flow diagram. | 7 |
| Characteristics of sources of evidence | 15 | For each source of evidence, present characteristics for which data were charted and provide the citations. | 8-9 |
| Critical appraisal within sources of evidence | 16 | If done, present data on critical appraisal of included sources of evidence (see item 12). | n.a. |
| Results of individual sources of evidence | 17 | For each included source of evidence, present the relevant data that were charted that relate to the review questions and objectives. | 9-15 |
| Synthesis of results | 18 | Summarize and/or present the charting results as they relate to the review questions and objectives. | 7-8 |
| **DISCUSSION** | | | |
| Summary of evidence | 19 | Summarize the main results (including an overview of concepts, themes, and types of evidence available), link to the review questions and objectives, and consider the relevance to key groups. | 16-18 |
| Limitations | 20 | Discuss the limitations of the scoping review process. | 20 |
| Conclusions | 21 | Provide a general interpretation of the results with respect to the review questions and objectives, as well as potential implications and/or next steps. | 20-21 |
| **FUNDING** | | | |
| Funding | 22 | Describe sources of funding for the included sources of evidence, as well as sources of funding for the scoping review. Describe the role of the funders of the scoping review. | 22 |

JBI = Joanna Briggs Institute; PRISMA-ScR = Preferred Reporting Items for Systematic reviews and Meta-Analyses extension for Scoping Reviews.

* Where *sources of evidence* (see second footnote) are compiled from, such as bibliographic databases, social media platforms, and Web sites.

† A more inclusive/heterogeneous term used to account for the different types of evidence or data sources (e.g., quantitative and/or qualitative research, expert opinion, and policy documents) that may be eligible in a scoping review as opposed to only studies. This is not to be confused with *information sources* (see first footnote).

‡ The frameworks by Arksey and O’Malley (6) and Levac and colleagues (7) and the JBI guidance (4, 5) refer to the process of data extraction in a scoping review as data charting*.*

§ The process of systematically examining research evidence to assess its validity, results, and relevance before using it to inform a decision. This term is used for items 12 and 19 instead of "risk of bias" (which is more applicable to systematic reviews of interventions) to include and acknowledge the various sources of evidence that may be used in a scoping review (e.g., quantitative and/or qualitative research, expert opinion, and policy document).

*From:* Tricco AC, Lillie E, Zarin W, O'Brien KK, Colquhoun H, Levac D, et al. PRISMA Extension for Scoping Reviews (PRISMA-ScR): Checklist and Explanation. Ann Intern Med. ;169:467–473. doi: 10.7326/M18-0850

# Additional File 2. Search Strategy

## **Medline (PubMed)**

**2018, May 22**

Database: Medline (Pub Med) <since its inception to 2018, May 22> Search Strategy:

-------------------------------------------------------------------------------------------------------

| **#** | **Searches** |  | **Results** |
| --- | --- | --- | --- |
|  |  |  |  |
| 1 | "Musculoskeletal Diseases"[Mesh] OR “Musculoskeletal Pain"[Mesh] |  | 1000580 |
| 2 | “LBP”[ti] OR “lumbar pain”[ti] OR “lumbosacral pain”[ti] OR “sacroiliac pain”[ti] OR “thoracolumbar pain”[ti] OR “Back Pain”[ti] OR “Low Back Pain”[ti] OR “Lower Back Pain”[ti] OR “Low Back Ache”[ti] OR “Back Ache”[ti] OR “Low Backache”[ti] OR Backache[ti] OR dorsalgia[ti] OR coccydynia[ti] OR spondylosis[ti] OR sciatica[ti] OR lumbago[ti] OR discitis[ti] OR “spinal fusion”[ti] OR postlaminectomy[ti] OR “failed back surgery”[ti] OR “herniated disk”[ti] OR hernia[ti] |  | 53462 |
| 3 | 2 NOT myelopathy |  | 52556 |
| 5 | “cervicothoracic pain”[ti] OR “Neck Pain”[ti] OR “Neck Ache”[ti] OR Cervicalgia[ti] OR Cervicodynia[ti] OR Neckache[ti] OR “Cervical Pain”[ti] OR torticollis[ti] OR cervicodynia[ti] OR whiplash[ti] OR “whiplash injury”[ti] OR “whiplash injuries”[ti] OR “whiplash associated disorder”[ti] OR “whiplash associated disorders”[ti] OR “craniocervical pain”[ti] OR “cervical headache”[ti] OR “cervicogenic headache”[ti] OR “neck headache”[ti] |  | 6490 |
| 6 | 5 NOT (genital diseases, female/ OR Uterus) |  | 6485 |
| 7 | 6 NOT (myelopathy) |  | 6345 |
| 8 | “scapular pain”[ti] OR “shoulder pain”[ti] OR “shoulder Impingement Syndrome”[ti] OR “rotator Cuff”[ti] OR ((shoulder[ti] OR elbow[ti]) AND (tendonitis[ti] OR tendinitis[ti] OR tendinopathy[ti] OR Bursitis[ti])) OR “elbow pain”[ti] OR "adhesive capsulitis"[ti] OR “frozen shoulder”[ti] OR brachialgia[ti] |  | 6722 |
| 9 | arthropathy[ti] OR Osteoarthrosis[ti] OR Osteoarthritis[ti] OR “osteoarthritic pain”[ti] OR “Hip osteoarthritis”[ti] OR “knee osteoarthritis”[ti] OR “hand osteoarthritis”[ti] OR “foot osteoarthritis”[ti] OR “spine osteoarthritis”[ti] OR “patellofemoral”[ti] OR “patellofemoral pain”[ti] OR “patellofemoral pain syndrome”[ti] OR “patellofemoral syndrome”[ti] OR ((patellar[ti]) AND (pain[ti] OR tendonitis[ti] OR tendinitis[ti] OR tendinopathy[ti] OR Bursitis[ti])) OR “patellofemoral pain”[ti] OR “patellofemoral pain syndrome”[ti] |  | 30955 |
| 10 | Fibromyalgia[ti] OR “Fibromyalgia Syndrome”[ti] OR Rheumatism[ti] OR “Muscular Rheumatism”[ti] OR Fibrositis[ti] OR "Myofascial Pain Syndrome"[ti] OR "Polymyalgia Rheumatica"[ti] OR "Rheumatoid Arthritis"[ti] OR Osteoarthropathy[ti] OR Arthritis[ti] OR Arthralgia[ti] OR Ankylosis[ti] OR Scoliosis[ti] OR Spondylitis[ti] OR Spondylosis[ti] OR “spondylitis ankylosing”[ti] |  | 130143 |
| 11 | 1 OR 3 OR 7 OR 8 OR 9 OR 10 |  | 1075034 |
| 12 | “Body Image”[Mesh] OR “Space Perception”[Mesh] OR “Form Perception”[Mesh] OR “Interoception”[Mesh] OR “Perceptual Distortion”[Mesh] OR “Size Perception”[Mesh] OR “Touch Perception”[Mesh] OR “Weight Perception”[Mesh] OR “Perceptual Disorder”[Mesh] |  | 77182 |
| 13 | “personal space” OR “peri personal space” OR “peripersonal space” OR “extra personal space” OR “extrapersonal space” OR “surrounding space” OR “spatial representation” OR “spatial perception” OR “spatial attention” OR "spatial sensory organization" |  | 7167 |
| 14 | “body perception” OR “body representation” OR “body attention” OR “bodily attention” OR “bodily perception” OR “bodily representation” OR “bodily attention” OR “body schema” OR “bodily schema” OR “body schemata” OR “bodily schemata” OR “superficial schema” OR “superficial schemata” OR “body map” OR “bodily map” OR “body form” OR “bodily form” OR “body image” OR “bodily image” OR “body awareness” OR “bodily awareness” OR embodiment OR “body consciousness” OR “bodily consciousness” OR “body ownership” OR “bodily ownership” OR “postural schema” OR “postural schemata” OR “postural image” OR “postural perception” OR “postural representation” OR “postural localization” OR “postural localisation” OR “postural map” OR “laterality judgment” OR “left right discrimination” OR “motor imagery” OR “mental representation” |  | 27244 |
| 15 | topognosis OR atopognosia OR autotopagnosia OR "self perception" OR "self recognition" OR "self representation" OR "self localisation" OR "self localization" OR "body localisation" OR "body localization" OR "bodily localisation" OR "bodily localization" OR “self location” OR “self localization” OR “self localisation” OR “self image” OR “body self” OR “self perception” OR “self representation” OR “self attribution” |  | 13057 |
| 16 | neglect OR “spatial neglect” OR “attentional neglect” OR “hemi spatial neglect” OR “hemispatial neglect” OR “sensory neglect” OR “motor neglect” OR “representational neglect” OR “personal neglect” OR inattention OR "attention deficit" OR “spatial deficit” OR “spatial attention deficit” OR “pseudo-neglect” OR “pseudo neglect” OR “body disownership” OR “bodily disownership” |  | 54454 |
| 17 | "subjective straight ahead" OR "subjective visual vertical" OR "subjective visual horizontal" OR "subjective vertical" OR "subjective horizontal" OR "subjective midline" OR "subjective body midline" OR "subjective bodily midline" OR “visual vertical” OR “visual horizontal” OR egocentric coordinat* OR “egocentric space” OR “egocentric space representation” OR “egocentric space perception” OR “allocentric space” OR “straight ahead” |  | 10564 |
| 18 | Interoception |  | 722 |
| 19 | somesthetic OR somesthesis OR "tactile perception" OR “tactile representation” OR “tactile discrimination” OR stereognosis OR astereognosia OR "sensory disturbance" OR kinesthesia OR kinesthesis OR kinaesthesia OR kinesthetic OR “position sense” OR “position error” OR “proprioception error” OR "visual representation" OR “visual perception” OR "visuomotor adaptation" |  | 73070 |
| 20 | 12 OR /13-19 |  | 218317 |
| 21 | 11 AND 20 |  | 2953 |
| 22 | 21 NOT (anorexia OR bulimia OR eating disorder) |  | 2919 |
| 23 | 22 AND (humans NOT animals) |  | 2701 |

### User Query for PubMed:

"Musculoskeletal Diseases"[Mesh] OR "Musculoskeletal Pain"[Mesh] OR (("LBP"[ti] OR "lumbar pain"[ti] OR "Lumbosacral Pain"[ti] OR "sacroiliac pain"[ti] OR "thoracolumbar pain"[ti] OR "Back Pain"[ti] OR "Low Back Pain"[ti] OR "Lower Back Pain"[ti] OR "Low Back Ache"[ti] OR "Back Ache"[ti] OR "Low Backache"[ti] OR Backache[ti] OR dorsalgia[ti] OR coccydynia[ti] OR spondylosis[ti] OR sciatica[ti] OR lumbago[ti] OR discitis[ti] OR "spinal fusion"[ti] OR postlaminectomy[ti] OR "failed back surgery"[ti] OR "herniated disk"[ti] OR hernia[ti]) NOT (myelopathy)) OR (("cervicothoracic pain"[ti] OR "Neck Pain"[ti] OR "Neck Ache"[ti] OR Cervicalgia[ti] OR Cervicodynia[ti] OR Neckache[ti] OR "Cervical Pain"[ti] OR torticollis[ti] OR cervicodynia[ti] OR whiplash[ti] OR "whiplash injury"[ti] OR "whiplash injuries"[ti] OR "whiplash associated disorder"[ti] OR "whiplash associated disorders"[ti] OR "craniocervical pain"[ti] OR "cervical headache"[ti] OR "cervicogenic headache"[ti] OR "neck headache"[ti]) NOT (genital diseases, female/ OR Uterus) NOT (myelopathy)) OR ("scapular pain"[ti] OR "shoulder pain"[ti] OR "shoulder impingement syndrome"[ti] OR "rotator Cuff"[ti] OR ((shoulder[ti] OR elbow[ti]) AND (tendonitis[ti] OR tendinitis[ti] OR tendinopathy[ti] OR Bursitis[ti])) OR "elbow pain"[ti] OR "adhesive capsulitis"[ti] OR "frozen shoulder"[ti] OR brachialgia[ti]) OR (arthropathy[ti] OR osteoarthrosis[ti] OR Osteoarthritis[ti] OR "osteoarthritic pain"[ti] OR "Hip osteoarthritis"[ti] OR "knee osteoarthritis"[ti] OR "osteoarthritic pain"[ti] OR "hand osteoarthritis"[ti] OR "foot osteoarthritis"[ti] OR "spine osteoarthritis"[ti] OR "patellofemoral"[ti] OR "patellofemoral pain"[ti] OR "patellofemoral pain syndrome"[ti] OR "patellofemoral syndrome" OR ((patellar) AND (pain[ti] OR tendonitis[ti] OR tendinitis[ti] OR tendinopathy[ti] OR Bursitis[ti]))) OR (Fibromyalgia[ti] OR "Fibromyalgia Syndrome"[ti] OR Rheumatism[ti] OR "Muscular Rheumatism"[ti] OR Fibrositis[ti] OR "Myofascial Pain Syndrome"[ti] OR "Polymyalgia Rheumatica"[ti] OR "Rheumatoid Arthritis"[ti] OR Osteoarthropathy[ti] OR Arthritis[ti] OR Arthralgia[ti] OR Ankylosis[ti] OR Scoliosis[ti] OR spondylitis[ti] OR spondylosis[ti] OR "spondylitis ankylosing"[ti]) AND ("Body Image"[Mesh] OR "Space Perception"[Mesh] OR "Form Perception"[Mesh] OR "Perceptual Distortion"[Mesh] OR "Interoception"[Mesh] OR "Size Perception"[Mesh] OR "Touch Perception"[Mesh] OR "Weight Perception"[Mesh] OR "Perceptual Disorder"[Mesh] OR "personal space" OR "peri personal space" OR "peripersonal space" OR "extra personal space" OR "extrapersonal space" OR "surrounding space" OR "spatial representation" OR "spatial perception" OR "spatial attention" OR "spatial sensory organization" OR "body perception" OR "body representation" OR "body attention" OR "bodily attention" OR "bodily perception" OR "bodily representation" OR "body schema" OR "bodily schema" OR "body schemata" OR "bodily schemata" OR "superficial schema" OR "superficial schemata" OR "body map" OR "bodily map" OR "body form" OR "bodily form" OR "body image" OR "bodily image" OR "body awareness" OR "bodily awareness" OR embodiment OR "body consciousness" OR "bodily consciousness" OR "body ownership" OR "bodily ownership" OR topognosis OR anosognosia OR atopognosia OR autotopagnosia OR "self perception" OR "self recognition" OR "self representation" OR "self localisation" OR "self localization" OR "body localisation" OR "body localization" OR "bodily localisation" OR "bodily localization" OR "postural schema" OR "postural schemata" OR "postural image" OR "postural perception" OR "postural representation" OR "postural localization" OR "postural localisation" OR "postural map" OR "laterality judgment" OR "left right discrimination" OR "motor imagery" OR "mental representation" OR "self location" OR "self localization" OR "self localisation" OR "self image" OR "body self" OR "self perception" OR "self representation" OR "self attribution" OR "spatial neglect" OR neglect OR "attentional neglect" OR "hemi spatial neglect" OR "hemispatial neglect" OR "sensory neglect" OR "motor neglect" OR "representational neglect" OR "personal neglect" OR inattention OR "attention deficit" OR "spatial deficit" OR "spatial attention deficit" OR "pseudo-neglect" OR "pseudo neglect" OR "body disownership" OR "bodily disownership" OR "subjective straight ahead" OR "subjective visual vertical" OR "subjective visual horizontal" OR "subjective vertical" OR "subjective horizontal" OR "subjective midline" OR "subjective body midline" OR "subjective bodily midline" OR "visual vertical" OR "visual horizontal" OR egocentric coordinat* OR "egocentric space" OR "egocentric space representation" OR "egocentric space perception" OR "allocentric space" OR "straight ahead" OR interoception OR somesthetic OR somesthesis OR "tactile perception" OR "tactile representation" OR "tactile discrimination" OR stereognosis OR astereognosia OR "sensory disturbance" OR kinesthesia OR kinesthesis OR kinaesthesia OR kinesthetic OR "position sense" OR "position error" OR "proprioception error" OR "visual representation" OR "visual perception" OR "visuomotor adaptation") NOT (anorexia OR bulimia OR eating disorder) AND (humans NOT animals)

# Additional File 2. Search Strategy

## Cochrane Library (Cochrane Database for Systematic Reviews – Database of Abstracts of Reviews of Effect – Cochrane Central Register of Controlled Trials)

**2018, Jun 01**

Database: Cochrane Library <since its inception to 2018, Jun 01> Search Strategy:

----------------------------------------------------------------------------------------------------

| **#** | **Searches** |  | **Results** |
| --- | --- | --- | --- |
|  |  |  |  |
| 1 | MeSH descriptor: [Musculoskeletal Diseases] explode all trees |  | 37614 |
| 2 | MeSH descriptor: [Musculoskeletal Pain] explode all trees |  | 650 |
| 3 | "musculoskeletal pain" or "musculoskeletal disorder" or "lumbosacral spine" or "lumbar spine" or "LBP" or "LBPP" or "sacroiliac disease" or "thoraco lumbar" or "back pain" or "low back pain" or "lower back disorder" or "lower back" or "backache" or "dorsalgia" or "coccydynia" or "sciatica" or "lumbago" or "discitis" or "spinal fusion" or "postlaminectomy" or "postlaminectomy syndrome" or "failed back surgery syndrome" or "herniated disc syndrome" or "herniated disc syndromes" or "herniated-disc syndrome" or "herniated-disc syndromes" or "hernia":ti,ab,kw (Word variations have been searched) |  | 23749 |
| 4 | cervicothoracic or "neck pain" or "neck ache" or "neck aches" or "cervicalgia" or "cervicodynia" or "neckache" or "cervical pain syndrome" or "torticollis" or "cervicodorsal" or "whiplash injuries" or "whiplash injury" or whiplash or "craniocervical" or "cervicogenic headache":ti,ab,kw (Word variations have been searched) |  | 3230 |
| 5 | "shoulder pain" or "shoulder impingement syndrome" or "rotator cuff" or "adhesive capsulitis" or "frozen shoulder" or "brachialgia":ti,ab,kw (Word variations have been searched) |  | 3158 |
| 6 | "shoulder":ti,ab,kw and "tendonitis":ti,ab,kw and "tendinitis":ti,ab,kw and "tendinopathy":ti,ab,kw and "bursitis":ti,ab,kw (Word variations have been searched) |  | 14 |
| 7 | "elbow":ti,ab,kw and "tendinitis":ti,ab,kw and "tendinopathy":ti,ab,kw and "tendonitis":ti,ab,kw and "bursitis":ti,ab,kw (Word variations have been searched) |  | 5 |
| 8 | "osteoarthropathy" or "arthropathy" or "osteoarthritis" or "osteoarthroses" or osteoarth or "patellofemoral pain" or "patellofemoral pain syndrome" or "patellofemoral syndrome" or "patellofemoral" or "patellar chondropathies"":ti,ab,kw (Word variations have been searched) |  | 12676 |
| 9 | "patellar":ti,ab,kw and "tendonitis" or "tendinitis" or "tendinopathy" or "bursitis":ti,ab,kw (Word variations have been searched) |  | 110 |
| 10 | fibromyalgia or "fibromyalgia syndrome" or "fibromyalgic syndrome" or "rheumatism" or "muscular rheumatism" or "fibrositis" or "myofascial pain syndrome":ti,ab,kw or "polymyalgia rheumatica" or "rheumatoid arthritis" or "arthritis" or "arthralgia":ti,ab,kw or "ankylosis" or "scoliosis" or "spondylitis" or "spondylosis" or "spondylitis ankylopoietica" or "spondyl-arthropathy" or "spondyl-arthropathies" or "spondyl-arthroses" or "spondyl-arthrosis" or "spondylarthritis" or "spondylarthropathies" or "spondylarthropathy" or "spondylarthrosis":ti,ab,kw (Word variations have been searched) |  | 24293 |
| 11 | 1 or /2-10 |  | 77172 |
| 12 | MeSH descriptor: [Body Image] explode all trees |  | 630 |
| 13 | MeSH descriptor: [Form Perception] explode all trees |  | 937 |
| 14 | MeSH descriptor: [Perceptual Distortion] explode all trees |  | 87 |
| 15 | MeSH descriptor: [Size Perception] explode all trees |  | 108 |
| 16 | MeSH descriptor: [Space Perception] explode all trees |  | 1879 |
| 17 | MeSH descriptor: [Visual Perception] explode all trees |  | 9070 |
| 18 | MeSH descriptor: [Weight Perception] explode all trees |  | 30 |
| 19 | MeSH descriptor: [Touch Perception] explode all trees |  | 99 |
| 20 | MeSH descriptor: [Interoception] explode all trees |  | 7 |
| 21 | MeSH descriptor: [Perceptual Disorders] explode all trees |  | 869 |
| 22 | "body image" or "body image disturbance" or "body image disturbances" or "space perception" or "peripersonal space" or "extrapersonal space" or "awareness" or "embodiment" or "weight perception" or "topognosis" or "topognosia" or "topognoses" or "anosognosia" or "self locating" or "self image" or "self images" or "postural sense" or "self perception" or "self perceptions" or "self attribution" or "interoceptive" or "interoceptively" or "somesthesia" or "somesthesic" or "somesthetic" or "neglect" or "inattention" or "pseudoneglect" or "egocentric" or "allocentric" or "attentional disturbance" or "inattentiveness" or "attention deficit disorder" or "stereognostic perception" or "stereognoses" or "stereognosis" or "stereognostic" or "astereognosia" or "astereognosis" or "sensory disorder" or "kinestheses" or "kinesthesia" or "kinesthesis" or "kinaesthesia" or "kinaesthesis" or "kinaesthetic" or "kinaesthetics" or "kinesthetic sense” |  | 14984 |
| 23 | 12 or /13-22 |  | 24307 |
| 24 | 11 and 23 |  | 540 |

# Additional File 2. Search Strategy

## PEDro (Physiotherapy Evidence Database)

**2018, Jul 09**

Database: PEDro<since its inception to 2018, Jul 09> Search Strategy:

----------------------------------------------------------------------------------------------------

| **#** | **Searches** |  | **Results** |
| --- | --- | --- | --- |
|  |  |  |  |
| 1 | body perception |  | 76 |
| 2 | somatoperception |  | 0 |
| 3 | body representation |  | 6 |
| 4 | somatorepresentation |  | 0 |
| 5 | weight perception |  | 34 |
| 6 | visual perception |  | 112 |
| 7 | form perception |  | 57 |
| 8 | size perception |  | 24 |
| 9 | touch perception |  | 4 |
| 10 | body image |  | 60 |
| 11 | body awareness |  | 70 |
| 12 | embodiment |  | 3 |
| 13 | perceptual distortion* |  | 0 |
| 14 | percept* disord* |  | 35 |
| 15 | space perception |  | 4 |
| 16 | peripersonal space |  | 0 |
| 17 | extrapersonal space |  | 0 |
| 18 | topognosis |  | 0 |
| 19 | topognoses |  | 0 |
| 20 | anosognosia |  | 0 |
| 21 | stereognosis |  | 3 |
| 22 | stereognoses |  | 0 |
| 23 | astereognosis |  | 0 |
| 24 | astereognoses |  | 0 |
| 25 | interoception |  | 2 |
| 26 | self perception* |  | 238 |
| 27 | somesthe* |  | 0 |
| 28 | neglect |  | 61 |
| 29 | self image |  | 38 |
|  | Total Records |  | 827 |

# Additional File 2. Search Strategy

## The Campbell Collaboration

**2018, Jul 10**

Database: The Campbell Collaboration <since its inception to 2018, Jul 10> Search Strategy:

----------------------------------------------------------------------------------------------------

| **#** | **Searches** |  | **Results** |
| --- | --- | --- | --- |
|  |  |  |  |
| 1 | body image |  | 10 |
| 2 | body perception |  | 0 |
| 3 | body representation |  | 0 |
| 4 | somatoperception |  | 0 |
| 5 | somatorepresentation |  | 0 |
| 6 | weight perception |  | 1 |
| 7 | visual perception |  | 0 |
| 8 | form perception |  | 2 |
| 9 | size perception |  | 1 |
| 10 | touch perception |  | 0 |
| 11 | embodiment |  | 0 |
| 12 | body awareness |  | 3 |
| 13 | perceptual distortion |  | 0 |
| 14 | space perception |  | 1 |
| 15 | peripersonal space |  | 0 |
| 16 | extrapersonal space |  | 0 |
| 17 | extra personal space |  | 0 |
| 18 | topognosis |  | 0 |
| 19 | topognoses |  | 0 |
| 20 | anosognosia |  | 0 |
| 21 | stereognosis |  | 0 |
| 22 | stereognoses |  | 0 |
| 23 | astereognosis |  | 0 |
| 24 | astereognoses |  | 0 |
| 25 | interoception |  | 0 |
| 26 | self perception |  | 1 |
| 27 | somesthesis |  | 0 |
| 28 | neglect |  | 10 |
| 29 | self image |  | 26 |
|  | Total Records |  | 55 |

# Additional File 2. Search Strategy

## SCOPUS

**2018, Jul 16**

Database: SCOPUS <since its inception to 2018, Jul 16> Search Strategy:

----------------------------------------------------------------------------------------------------

| **#** | **Searches** |  | **Results** |
| --- | --- | --- | --- |
|  |  |  |  |
| 1 | (TITLE("musculoskeletal pain") OR TITLE("musculoskeletal disorder") OR TITLE("lumbosacral spine") OR TITLE("lumbar spine") OR TITLE(LBP) OR TITLE("lumbar pain") OR TITLE("lumbosacral pain") OR TITLE("sacroiliac pain") OR TITLE("sacroiliac disease") OR TITLE("thoracolumbar pain") OR TITLE("back pain") OR TITLE("low back pain") OR TITLE("lower back pain") OR TITLE("low back ache") OR TITLE("back ache") OR TITLE("backache") OR TITLE("low backache") OR TITLE(backache) OR TITLE(dorsalgia) OR TITLE(coccydynia) OR TITLE(spondylosis) OR TITLE(sciatica) OR TITLE(lumbago) OR TITLE(discitis) OR TITLE("spinal fusion") OR TITLE(postlaminectomy) OR TITLE("failed back surgery") OR TITLE("herniated disk") OR TITLE(hernia)) AND NOT INDEX(MEDLINE) |  | 27430 |
| 2 | #1 AND NOT myelopathy |  | 27173 |
| 3 | (TITLE("cervicothoracic pain") OR TITLE("neck pain") OR TITLE("Neck Ache") OR TITLE(Cervicalgia) OR TITLE(Cervicodynia) OR TITLE(Neckache) OR TITLE("Cervical Pain") OR TITLE(torticollis) OR TITLE(cervicodynia) OR TITLE(whiplash) OR TITLE("whiplash injury") OR TITLE("whiplash injuries") OR TITLE("whiplash associated disorder") OR TITLE("whiplash associated disorders") OR TITLE("craniocervical pain") OR TITLE("cervical headache") OR TITLE("cervicogenic headache") OR TITLE("neck headache") OR TITLE("cervical pain syndrome") OR TITLE("craniocervical") OR TITLE("cervicogenic headache")) AND NOT INDEX(MEDLINE) |  | 3053 |
| 4 | #3 AND NOT myelopathy |  | 2948 |
| 5 | (TITLE("scapular pain") OR TITLE("shoulder pain") OR TITLE("shoulder Impingement Syndrome") OR TITLE("rotator Cuff") OR ((TITLE(shoulder) OR TITLE(elbow)) AND (TITLE(tendonitis) OR TITLE(tendinitis) OR TITLE(tendinopathy) OR TITLE(Bursitis))) OR TITLE("elbow pain") OR TITLE("adhesive capsulitis") OR TITLE("frozen shoulder") OR TITLE (brachialgia)) AND NOT INDEX(MEDLINE) |  | 3621 |
| 6 | (TITLE(arthropathy) OR TITLE(Osteoarthrosis) OR TITLE(Osteoarthritis) OR TITLE(“osteoarthritic pain”) OR TITLE(“Hip osteoarthritis”) OR TITLE(“knee osteoarthritis”) OR TITLE(“hand osteoarthritis”) OR TITLE(“foot osteoarthritis”) OR TITLE(“spine osteoarthritis”) OR TITLE(“patellofemoral”) OR TITLE(“patellofemoral pain”) OR TITLE(“patellofemoral pain syndrome”) OR TITLE(“patellofemoral syndrome”) OR ((TITLE(patellar)) AND (TITLE(pain) OR TITLE(tendonitis) OR TITLE(tendinitis) OR TITLE(tendinopathy) OR TITLE(Bursitis))) OR TITLE(“patellofemoral pain”) OR TITLE(“patellofemoral pain syndrome”)) AND NOT INDEX(MEDLINE) |  | 12487 |
| 7 | (TITLE(Fibromyalgia) OR TITLE("Fibromyalgia Syndrome") OR TITLE(Rheumatism) OR TITLE("Muscular Rheumatism") OR TITLE(Fibrositis) OR TITLE("Myofascial Pain Syndrome") OR TITLE("Polymyalgia Rheumatica") OR TITLE("Rheumatoid Arthritis") OR TITLE(Osteoarthropathy) OR TITLE(Arthritis) OR TITLE(Arthralgia) OR TITLE(Ankylosis) OR TITLE(Scoliosis) OR TITLE(Spondylitis) OR TITLE(Spondylosis) OR TITLE("spondylitis ankylosing")) AND NOT INDEX(MEDLINE) |  | 42737 |
| 8 | 2 or /4-7 |  | 87701 |
| 9 | ("Body Image" OR "body image disturbance" OR "body image disturbances" OR awareness OR embodiment OR "Space Perception" OR "Form Perception" OR "visual perception" OR "Size Perception" OR "Touch Perception" OR "Weight Perception" OR "Perceptual Disorder" OR Interoception OR interoceptive OR interoceptively OR "Perceptual Distortion") AND NOT INDEX(MEDLINE) |  | 536641 |
| 10 | (“personal space”) OR “peri personal space” OR “peripersonal space” OR “extra personal space” OR “extrapersonal space” OR “surrounding space” OR “spatial representation” OR “spatial perception” OR “spatial attention” OR "spatial sensory organization") AND NOT INDEX(MEDLINE) |  | 33570 |
| 11 | (topognosis OR topognosia OR topognoses OR anosognosia OR "self locating" OR "self image" OR "self images" OR "postural sense" OR "self perception" OR "self perceptions" OR "self attribution" OR somesthesia OR somesthesic OR somesthetic OR neglect OR inattention OR pseudoneglect OR egocentric OR allocentric OR "attentional disturbance" OR inattentiveness OR "attention deficit disorder" OR "stereognostic perception" OR stereognoses OR "stereognosis" OR stereognostic OR astereognosia OR astereognosis OR "sensory disorder" OR kinestheses OR kinesthesia OR kinesthesis OR kinaesthesia OR kinaesthesis OR kinaesthetic OR kinaesthetics OR "kinesthetic sense”) NOT INDEX(MEDLINE) |  | 78208 |
| 12 | 9 OR /10-11 |  | 639401 |
| 13 | 8 AND 12 |  | 996 |

# Additional File 2. Search Strategy

## PsychINFO (EBSCOhost)

**2018, Jul 24**

Database: PsychINFO <since its inception to 2018, Jul 24> Search Strategy:

-------------------------------------------------------------------------------------------------------

| **#** | **Searches** |  | **Results** |
| --- | --- | --- | --- |
|  |  |  |  |
| 1 | MA Musculoskeletal Diseases" OR “Musculoskeletal Pain" |  | 1403 |
| 2 | TI “Musculoskeletal Diseases” OR “Musculoskeletal Pain” OR “LBP” OR “lumbar pain” OR “lumbosacral pain” OR “sacroiliac pain” OR “thoracolumbar pain” OR “Back Pain” OR “Low Back Pain” OR “Lower Back Pain” OR “Low Back Ache” OR “Back Ache” OR “Low Backache” OR Backache OR dorsalgia OR coccydynia OR spondylosis OR sciatica OR lumbago OR discitis OR “spinal fusion” OR postlaminectomy OR “failed back surgery” OR “herniated disk” OR hernia |  | 4257 |
| 3 | “cervicothoracic pain” OR “Neck Pain” OR “Neck Ache” OR Cervicalgia OR Cervicodynia OR Neckache OR “Cervical Pain” OR torticollis OR cervicodynia OR whiplash OR “whiplash injury” OR “whiplash injuries” OR “whiplash associated disorder” OR “whiplash associated disorders” OR “craniocervical pain” OR “cervical headache” OR “cervicogenic headache” OR “neck headache” |  | 853 |
| 4 | “scapular pain” OR “shoulder pain” OR “shoulder Impingement Syndrome” OR “rotator Cuff” OR “shoulder tendonitis” OR “shoulder tendinitis” OR “shoulder tendinopathy” OR “shoulder bursitis” OR “elbow tendonitis” OR “elbow tendinitis” OR “elbow tendinopathy” OR “elbow bursitis” OR “elbow pain” OR "adhesive capsulitis" OR “frozen shoulder” OR brachialgia |  | 151 |
| 5 | Arthropathy OR Osteoarthrosis OR Osteoarthritis OR “osteoarthritic pain” OR “Hip osteoarthritis” OR “knee osteoarthritis” OR “hand osteoarthritis” OR “foot osteoarthritis” OR “spine osteoarthritis” OR “patellofemoral” OR “patellofemoral pain” OR “patellofemoral pain syndrome” OR “patellofemoral syndrome” OR “patellar pain” OR “patellar tendonitis” OR “patellar tendinopathy” OR “patellar bursitis” OR “patellofemoral pain” OR “patellofemoral pain syndrome” |  | 737 |
| 6 | Fibromyalgia OR “Fibromyalgia Syndrome” OR Rheumatism OR “Muscular Rheumatism” OR Fibrositis OR "Myofascial Pain Syndrome" OR "Polymyalgia Rheumatica" OR "Rheumatoid Arthritis" OR Osteoarthropathy OR Arthritis OR Arthralgia OR Ankylosis OR Scoliosis OR Spondylitis OR Spondylosis OR “spondylitis ankylosing” |  | 3974 |
| 7 | 1 OR /2-6 |  | 11446 |
| 8 | MA “Body Image” OR “Space Perception” OR “Form Perception” OR “Interoception” OR “Perceptual Distortion” OR “Size Perception” OR “Touch Perception” OR “Weight Perception” OR “Perceptual Disorder” |  | 38089 |
| 9 | TI “Body Image” OR “Space Perception” OR “Form Perception” OR “Interoception” OR “Perceptual Distortion” OR “Size Perception” OR “Touch Perception” OR “Weight Perception” OR “Perceptual Disorder” |  | 5517 |
| 10 | TI “personal space” OR “peri personal space” OR “peripersonal space” OR “extra personal space” OR “extrapersonal space” OR “surrounding space” OR “spatial representation” OR “spatial perception” OR “spatial attention” OR "spatial sensory organization" |  | 1961 |
| 11 | TI “body perception” OR “body representation” OR “body attention” OR “bodily attention” OR “bodily perception” OR “bodily representation” OR “bodily attention” OR “body schema” OR “bodily schema” OR “body schemata” OR “bodily schemata” OR “superficial schema” OR “superficial schemata” OR “body map” OR “bodily map” OR “body form” OR “bodily form” OR “body image” OR “bodily image” OR “body awareness” OR “bodily awareness” OR embodiment OR “body consciousness” OR “bodily consciousness” OR “body ownership” OR “bodily ownership” OR “postural schema” OR “postural schemata” OR “postural image” OR “postural perception” OR “postural representation” OR “postural localization” OR “postural localisation” OR “postural map” OR “laterality judgment” OR “left right discrimination” OR “motor imagery” OR “mental representation” |  | 7348 |
| 12 | TI topognosis OR atopognosia OR autotopagnosia OR "self perception" OR "self recognition" OR "self representation" OR "self localisation" OR "self localization" OR "body localisation" OR "body localization" OR "bodily localisation" OR "bodily localization" OR “self location” OR “self localization” OR “self localisation” OR “self image” OR “body self” OR “self perception” OR “self representation” OR “self attribution” |  | 2764 |
| 13 | TI neglect OR “spatial neglect” OR “attentional neglect” OR “hemi spatial neglect” OR “hemispatial neglect” OR “sensory neglect” OR “motor neglect” OR “representational neglect” OR “personal neglect” OR inattention OR "attention deficit" OR “spatial deficit” OR “spatial attention deficit” OR “pseudo-neglect” OR “pseudo neglect” OR “body disownership” OR “bodily disownership” |  | 11753 |
| 14 | TI "subjective straight ahead" OR "subjective visual vertical" OR "subjective visual horizontal" OR "subjective vertical" OR "subjective horizontal" OR "subjective midline" OR "subjective body midline" OR "subjective bodily midline" OR “visual vertical” OR “visual horizontal” OR egocentric coordinat* OR “egocentric space” OR “egocentric space representation” OR “egocentric space perception” OR “allocentric space” OR “straight ahead” |  | 192 |
| 15 | TI interoception |  | 131 |
| 16 | TI somesthetic OR somesthesis OR "tactile perception" OR “tactile representation” OR “tactile discrimination” OR stereognosis OR astereognosia OR "sensory disturbance" OR kinesthesia OR kinesthesis OR kinaesthesia OR kinesthetic OR “position sense” OR “position error” OR “proprioception error” OR "visual representation" OR “visual perception” OR "visuomotor adaptation" |  | 3448 |
| 17 | 12 OR /8-16 |  | 244891 |
| 18 | 7 AND 17 |  | 310 |

# Additional File 2. Search Strategy

## Web of Science

**2018, Jul 25**

Database: Web of Science <since its inception to 2018, Jul 25> Search Strategy:

-------------------------------------------------------------------------------------------------------

| **#** | **Searches** |  | **Results** |
| --- | --- | --- | --- |
|  |  |  |  |
| 1 | TI=("Musculoskeletal Diseases" OR "Musculoskeletal Pain" OR "LBP" OR "lumbar pain" OR "lumbosacral pain" OR "sacroiliac pain" OR "thoracolumbar pain" OR "Back Pain" OR "Low Back Pain" OR "Lower Back Pain" OR "Low Back Ache" OR "Back Ache" OR "Low Backache" OR Backache OR dorsalgia OR coccydynia OR spondylosis OR sciatica OR lumbago OR discitis OR "spinal fusion" OR postlaminectomy OR "failed back surgery" OR "herniated disk" OR hernia) |  | 43416 |
| 2 | TI=(“cervicothoracic pain” OR “Neck Pain” OR “Neck Ache” OR Cervicalgia OR Cervicodynia OR Neckache OR “Cervical Pain” OR torticollis OR cervicodynia OR whiplash OR “whiplash injury” OR “whiplash injuries” OR “whiplash associated disorder” OR “whiplash associated disorders” OR “craniocervical pain” OR “cervical headache” OR “cervicogenic headache” OR “neck headache”) |  | 5372 |
| 3 | TI=(“scapular pain” OR “shoulder pain” OR “shoulder Impingement Syndrome” OR “rotator Cuff” OR “shoulder tendonitis” OR “shoulder tendinitis” OR “shoulder tendinopathy” OR “shoulder bursitis” OR “elbow tendonitis” OR “elbow tendinitis” OR “elbow tendinopathy” OR “elbow bursitis” OR “elbow pain” OR "adhesive capsulitis" OR “frozen shoulder” OR brachialgia) |  | 7316 |
| 4 | TI=(Arthropathy OR Osteoarthrosis OR Osteoarthritis OR “osteoarthritic pain” OR “Hip osteoarthritis” OR “knee osteoarthritis” OR “hand osteoarthritis” OR “foot osteoarthritis” OR “spine osteoarthritis” OR “patellofemoral” OR “patellofemoral pain” OR “patellofemoral pain syndrome” OR “patellofemoral syndrome” OR “patellar pain” OR “patellar tendonitis” OR “patellar tendinopathy” OR “patellar bursitis” OR “patellofemoral pain” OR “patellofemoral pain syndrome”) |  | 37191 |
| 5 | TI=(Fibromyalgia OR “Fibromyalgia Syndrome” OR Rheumatism OR “Muscular Rheumatism” OR Fibrositis OR "Myofascial Pain Syndrome" OR "Polymyalgia Rheumatica" OR "Rheumatoid Arthritis" OR Osteoarthropathy OR Arthritis OR Arthralgia OR Ankylosis OR Scoliosis OR Spondylitis OR Spondylosis OR “spondylitis ankylosing”) |  | 138018 |
| 6 | 1 or /2-5 |  | 228092 |
| 7 | TI=(“Body Image” OR “Space Perception” OR “Form Perception” OR “Interoception” OR “Perceptual Distortion” OR “Size Perception” OR “Touch Perception” OR “Weight Perception” OR “Perceptual Disorder”) |  | 5314 |
| 8 | TI=(“personal space” OR “peri personal space” OR “peripersonal space” OR “extra personal space” OR “extrapersonal space” OR “surrounding space” OR “spatial representation” OR “spatial perception” OR “spatial attention” OR "spatial sensory organization") |  | 2382 |
| 9 | TI=(“body perception” OR “body representation” OR “body attention” OR “bodily attention” OR “bodily perception” OR “bodily representation” OR “bodily attention” OR “body schema” OR “bodily schema” OR “body schemata” OR “bodily schemata” OR “superficial schema” OR “superficial schemata” OR “body map” OR “bodily map” OR “body form” OR “bodily form” OR “body image” OR “bodily image” OR “body awareness” OR “bodily awareness” OR embodiment OR “body consciousness” OR “bodily consciousness” OR “body ownership” OR “bodily ownership” OR “postural schema” OR “postural schemata” OR “postural image” OR “postural perception” OR “postural representation” OR “postural localization” OR “postural localisation” OR “postural map” OR “laterality judgment” OR “left right discrimination” OR “motor imagery” OR “mental representation”) |  | 9972 |
| 10 | TI=(topognosis OR atopognosia OR autotopagnosia OR "self perception" OR "self recognition" OR "self representation" OR "self localisation" OR "self localization" OR "body localisation" OR "body localization" OR "bodily localisation" OR "bodily localization" OR “self location” OR “self localization” OR “self localisation” OR “self image” OR “body self” OR “self perception” OR “self representation” OR “self attribution”) |  | 4130 |
| 11 | TI=(neglect OR “spatial neglect” OR “attentional neglect” OR “hemi spatial neglect” OR “hemispatial neglect” OR “sensory neglect” OR “motor neglect” OR “representational neglect” OR “personal neglect” OR inattention OR "attention deficit" OR “spatial deficit” OR “spatial attention deficit” OR “pseudo-neglect” OR “pseudo neglect” OR “body disownership” OR “bodily disownership”) |  | 28836 |
| 12 | TI=("subjective straight ahead" OR "subjective visual vertical" OR "subjective visual horizontal" OR "subjective vertical" OR "subjective horizontal" OR "subjective midline" OR "subjective body midline" OR "subjective bodily midline" OR “visual vertical” OR “visual horizontal” OR egocentric coordinat* OR “egocentric space” OR “egocentric space representation” OR “egocentric space perception” OR “allocentric space” OR “straight ahead”) |  | 415 |
| 13 | TI=(interoception) |  | 182 |
| 14 | TI=(somesthetic OR somesthesis OR "tactile perception" OR “tactile representation” OR “tactile discrimination” OR stereognosis OR astereognosia OR "sensory disturbance" OR kinesthesia OR kinesthesis OR kinaesthesia OR kinesthetic OR “position sense” OR “position error” OR “proprioception error” OR "visual representation" OR “visual perception” OR "visuomotor adaptation") |  | 4806 |
| 15 | 7 or /8-14 |  | 5162 |
| 16 | 6 AND 15 |  | 244 |

# Additional File 2. Search Strategy

## TRIP Database

**2018, Jul 25**

Database: TRIP Database <since its inception to 2018, Jul 25> Search Strategy:

-------------------------------------------------------------------------------------------------------

| **#** | **Searches** |  | **Results** |
| --- | --- | --- | --- |
|  |  |  |  |
| 1 | “Musculoskeletal*” OR “musculoskeletal pain” OR “rheumatic*” OR “rheumatic disease” |  | 80924 |
| 2 | “body image” OR body perception OR body representation |  | 15485 |
| 3 | 1 AND 2 |  | 1048 |

# Additional File 2. Search Strategy

## ProQuest Central

**2018, Jul 27**

Database: ProQuest Central <since its inception to 2018, Jul 27> Search Strategy:

-------------------------------------------------------------------------------------------------------

| **#** | **Searches** |  | **Results** |
| --- | --- | --- | --- |
| 1 | MESH.EXACT.EXPLODE("Musculoskeletal Pain:C.23.888.592.612.547") OR MESH.EXACT.EXPLODE("Musculoskeletal Pain:F.02.830.816.353") OR MESH.EXACT.EXPLODE("Musculoskeletal Pain:C.05.651.538") OR MESH.EXACT.EXPLODE("Musculoskeletal Diseases") OR MESH.EXACT.EXPLODE("Musculoskeletal Pain:G.11.561.790.353") |  | 68965 |
| 2 | MESH.EXACT.EXPLODE("Polymyalgia Rheumatica:C.05.651.742") OR MESH.EXACT.EXPLODE("Rheumatic Diseases:C.05.799") OR MESH.EXACT.EXPLODE("Rheumatic Diseases:C.17.300.775") OR MESH.EXACT.EXPLODE("Polymyalgia Rheumatica:C.05.799.720") OR MESH.EXACT.EXPLODE("Polymyalgia Rheumatica:C.17.300.775.720") OR MESH.EXACT.EXPLODE("Osteoarthritis:C.05.799.613") OR MESH.EXACT.EXPLODE("Osteoarthritis:C.05.550.114.606") OR MESH.EXACT.EXPLODE("Fibromyalgia:C.10.668.491.425") OR MESH.EXACT.EXPLODE("Fibromyalgia:C.05.651.324") OR MESH.EXACT.EXPLODE("Fibromyalgia:C.05.799.321") |  | 13945 |
| 3 | 1 or 2 |  | 68965 |
| 4 | ti("body perception" OR "body representation" OR "body image" OR somatosensation OR somatorepresentation) |  | 8162 |
| 5 | MESH.EXACT.EXPLODE("Body Image:F.01.752.747.792.110") OR MESH.EXACT.EXPLODE("Body Image:F.02.463.593.112") |  | 1353 |
| 6 | MESH.EXACT("Perceptual Distortion") OR MESH.EXACT.EXPLODE("Perception") OR MESH.EXACT("Perceptual Disorders") |  | 36041 |
| 7 | MESH.EXACT("Visual Perception") OR MESH.EXACT("Touch Perception") OR MESH.EXACT("Depth Perception") OR MESH.EXACT("Illusions") OR MESH.EXACT("Size Perception") OR MESH.EXACT("Weight Perception") OR MESH.EXACT("Interoception") OR MESH.EXACT("Space Perception") OR MESH.EXACT("Form Perception") |  | 8742 |
| 8 | 4 or /5-7 |  | 43940 |
| 9 | 3 AND 8 |  | 472 |

# Additional File 2. Search Strategy

## PQDT Open

**2018, Aug 19**

Database: PQDT Open <since its inception to 2018, Aug 19> Search Strategy:

-------------------------------------------------------------------------------------------------------

| **#** | **Searches** |  | **Results** |
| --- | --- | --- | --- |
|  |  |  |  |
| 1 | Musculoskeletal OR “musculoskeletal pain” OR rheumatic OR “rheumatic disease” |  | 1051 |
| 2 | "lumbar pain" OR "Low Back Pain" OR sciatica OR "failed back surgery" OR "neck pain" OR "cervical pain" OR cervicalgia OR osteoarthritis OR osteoarthrosis OR fibromyalgia OR rheumatism |  | 1075 |
| 3 | 1 or 2 |  | 1747 |
| 4 | topognosis OR "mental representation" OR "laterality judgment" OR "left right discrimination" OR "postural schema" OR "postural image" OR "postural perception" OR "body form" OR "body awareness" OR "body ownership" OR embodiment OR "body attention" OR "superficial schema" OR "body map" OR personal space" OR "peripersonal space" OR "extra personal space" OR "space representation" OR "spatial attention" OR "body perception" OR "body representation" OR "body image" OR interoception OR "space perception" OR "body illusion" OR "form perception" OR "perceptual distortion" OR "size perception" OR "touch perception" OR "weight perception" OR "perceptual disorder" OR "tactile perception" OR "tactile representation" OR "tactile discrimination" |  | 5848 |
| 5 | 3 AND 5 |  | 390 |

# Additional File 2. Search Strategy

## ClinicalTrials.gov

**2018, Sep 18**

Database: ClinicalTrials.gov <since its inception to 2018, Sep 18> Search Strategy:

-------------------------------------------------------------------------------------------------------

| **#** | **Searches** |  | **Results** |
| --- | --- | --- | --- |
|  |  |  |  |
| 1 | Musculoskeletal Pain |  |  |
| 2 | "body perception" OR "body representation" OR "body schema" OR "body image" OR "perceptual distortion" OR "body illusion" OR "perceptual disorder" OR "body awareness" |  |  |
| 3 | 1 AND 2 |  | 4 |
| 4 | Musculoskeletal Diseases or Conditions |  |  |
| 5 | "body perception" OR "body representation" OR "body schema" OR "body image" OR "perceptual distortion" OR "body illusion" OR "perceptual disorder" OR "body awareness" |  |  |
| 6 | 4 AND 5 |  | 24 |
| 7 | Rheumatic Diseases |  |  |
| 8 | "body perception" OR "body representation" OR "body schema" OR "body image" OR "perceptual distortion" OR "body illusion" OR "perceptual disorder" OR "body awareness" |  |  |
| 9 | 7 AND 8 |  | 7 |
| 10 | Total Records |  | 35 |

# Additional File 2. Search Strategy

## Google Scholar

**2018, Aug 19**

Database: Google Scholar <since its inception to 2018, Aug 19> Search Strategy:

-------------------------------------------------------------------------------------------------------

**Filter: first 100 record filtered for "most relevant**"

**Query Used:**

("body perception" OR "body representation" OR body schema" OR "body image" OR "perceptual distortion" OR "body illusion" OR "perceptual disorder" OR "body awareness") AND (musculoskeletal* OR rheumatic*)

# Additional File 2. Search Strategy

## Google

**2018, Aug 19**

Database: Google <since its inception to 2018, Aug 19> Search Strategy:

-------------------------------------------------------------------------------------------------------

**Filter: first 100 record.**

**Query Used:**

("body perception" OR "body representation" OR body schema" OR "body image" OR "perceptual distortion" OR "body illusion" OR "perceptual disorder" OR "body awareness") AND (musculoskeletal* OR rheumatic)

# Additional File 2. Search Strategy

## Electronic Journal Hand Searching: https://www.scimagojr.com/

**2019, February 27**

Lists of the four higher-impact factor electronic journal within the field of MDRDs searched on https://www.scimagojr.com/ and manually scanned <since its inception to 2019 Mar 15>:

-------------------------------------------------------------------------------------------------------

**Filter: Categories (Physical Therapy, Sports Therapy and Rehabilitation); first four higher-impact factor journal**

| **Ranking** | **Journal** |  | **Cites/Doc. (2 years) 2018** |
| --- | --- | --- | --- |
|  |  |  |  |
| 1 | Sports Medicine |  | 7.17 |
| 2 | British Journal of Sports Medicine |  | 6.99 |
| 3 | American Journal of Sports Medicine |  | 6.16 |
| 4 | Journal of Physiotherapy |  | 5.41 |

# Additional File 3 – Supplementary Table S2 - Questions for the determination of clinical relevance of included studies

| **Questions to Determine if Results Are Clinically Relevant** (adapted from Furlan et al. 1999 (1)) | | | |
| --- | --- | --- | --- |
| Based on the data provided, can you determine if the results will be clinically relevant? |  |  |  |
| Are the patients described in detail so that you can decide whether they are comparable to those that you see in your practice? | □ Yes | □ No | □ Unsure |
| Are the interventions and treatment settings described well enough so that you can provide the same for your patients? | □ Yes | □ No | □ Unsure |
| Were all clinically relevant outcomes measured and reported? | □ Yes | □ No | □ Unsure |
| Is the size of the effect clinically important?* | □ Yes | □ No | □ Unsure |
| Are the likely treatment benefits worth the potential harms? | □ Yes | □ No | □ Unsure |

*For low-back pain, consider 30% on VAS/NRS for pain as clinically significant (2,3) and 2 to 3 points (or 8 to 12%) on the Roland-Morris Disability Questionnaire for function (2,4).

*For neck pain, consider 3.5 to 5 U on the 50-U Neck Pain Disability Index or 7 to 10% change (5,6) for function and 2.5 on an 10-U NRS (25% change) for pain (5).

**REFERENCES:**

1. Furlan AD, Pennick V, Bombardier C, van Tulder M, Editorial Board, Cochrane Back Review Group. 2009 updated method guidelines for systematic reviews in the Cochrane Back Review Group. Spine. 2009 Aug 15;34(18):1929–41.

2. Ostelo RWJG, Deyo RA, Stratford P, Waddell G, Croft P, Von Korff M, et al. Interpreting change scores for pain and functional status in low back pain: towards international consensus regarding minimal important change. Spine. 2008 Jan 1;33(1):90–4.

3. Farrar JT, Young JP, LaMoreaux L, Werth JL, Poole RM. Clinical importance of changes in chronic pain intensity measured on an 11-point numerical pain rating scale. Pain. 2001 Nov;94(2):149–58.

4. Bombardier C, Hayden J, Beaton DE. Minimal clinically important difference. Low back pain: outcome measures. J Rheumatol. 2001 Feb;28(2):431–8.

5. Pool JJM, Ostelo RWJG, Hoving JL, Bouter LM, de Vet HCW. Minimal clinically important change of the Neck Disability Index and the Numerical Rating Scale for patients with neck pain. Spine. 2007 Dec 15;32(26):3047–51.

6. Stratford P, Riddle D, Binkley J, Spadoni G, Westaway M, Padfiled B. Using the Neck Disability Index to make decisions concerning individual patients. Physiother Can. 2009;Stratford PW, Riddle DL, Binkley JM, et al. Using the Neck Disability Index to make decisions concerning individual patients. Physiother Can 1999; Spring:107–19.:107–19.

# Additional File 4 – Supplementary Table S3 - Evaluation of clinical relevance for assessment studies

|  | \|  \| \| --- \| | |  | | |  | |  | |  | |  | |
| --- | --- | --- | --- | --- | --- | --- | --- | --- | --- | --- | --- | --- | --- | --- |
|  |  | |  | | |  | |  | |  | |  | |
|  |  | |  | | |  | |  | |  | |  | |
|  |  | |  | | |  | |  | |  | |  | |
|  |  | |  | | |  | |  | |  | |  | |
|  |  | |  | | |  | |  | |  | |  | |
|  |  | |  | | |  | |  | |  | |  | |
|  |  | |  | | |  | |  | |  | |  | |
|  |  | |  | | |  | |  | |  | |  | |
|  |  | |  | | |  | |  | |  | |  | |
|  |  | |  | | |  | |  | |  | |  | |
|  |  | |  | | |  | |  | |  | |  | |
| **Studies** |  | |  | | |  | |  | |  | |  | |
|  |  | |  | | |  | |  | |  | |  | |
| Wand et al. (2013) | Yes | Yes | | | Yes | | No | | Yes | | Patients not suitable to perform repeated movement assessment were excluded even if tests provided were on static posture: this may have biased the enrolment of patients with higher disability and distress levels | |  |
| Adamczyk et al. (2018a) | No | Yes | | | Yes | | Unc. | | Yes | |  | |  |
| Adamczyk et al. (2018b) | No | Yes | | | Yes | | Unc. | | Yes | | The study lacks of a healthy subjects control group and no comparisons can be made to assess the clinical relevance of findings | |  |
| Wand et al. (2010) | No | Yes | | | Yes | | Unc. | | Yes | |  | |  |
| Moseley (2008) | No | Yes | | | No | | Unc. | | Yes | |  | |  |
| Lauche et al. (2012) | No | Yes | | | No | | Unc. | | Yes | | No measure of pain and disability were administered | |  |
| Mibu et al. (2015) | Unc. | Unc. | | | Yes | | No | | Yes | | The study was in Japanese language, with only the abstract and the main tables' content in English: this issues have limited data extraction and clinical interpretation of findings | |  |
|  | | | |  |  |  |  |  |  |  |  |  |  |
|  | \|  \| \| --- \| | |  | | |  | |  | |  | |  | |
|  |  | |  | | |  | |  | |  | |  | |
|  |  | |  | | |  | |  | |  | |  | |
|  |  | |  | | |  | |  | |  | |  | |
|  |  | |  | | |  | |  | |  | |  | |
|  |  | |  | | |  | |  | |  | |  | |
|  |  | |  | | |  | |  | |  | |  | |
|  |  | |  | | |  | |  | |  | |  | |
|  |  | |  | | |  | |  | |  | |  | |
|  |  | |  | | |  | |  | |  | |  | |
|  |  | |  | | |  | |  | |  | |  | |
|  |  | |  | | |  | |  | |  | |  | |
| **Studies** |  | |  | | |  | |  | |  | |  | |
|  |  | |  | | |  | |  | |  | |  | |
| Nishigami et al. (2015) | No | Yes | | | Yes | | Unc. | | Yes | |  | |  |
| Moerira et al. (2018) | No | Yes | | | No | | Unc. | | Yes | |  | |  |
| Valenzuela-Moguillansky (2013) | No | Yes | | | No | | Unc. | | Yes | |  | |  |
| Wand et al. (2014) | Yes | Yes | | | Yes | | Yes | | Yes | | Patients not suitable to perform repeated movement assessment were excluded even if tests provided were on static posture: this may have biased the enrolment of patients with higher disability and distress levels | |  |
| Wand et al. (2016) | Yes | Yes | | | Yes | | Yes | | Yes | |  | |  |
| Beales et al. (2016) | No | Yes | | | Yes | | Yes | | Yes | |  | |  |
| Wand et a. (2017) | No | Yes | | | Yes | | Yes | | Yes | |  | |  |
| Nishigami et al. (2018) | No | Yes | | | Yes | | Yes | | Yes | |  | |  |
| Jassens et al. (2017) | No | Yes | | | Yes | | Yes | | Yes | | Minimal Detectable Change (MDC) of the scale was 30% of scale range, thus referring to a non-sufficient measurement error: a maximum MDC of 20% of the scale range is considered as acceptable | |  |
|  |  | |  | | |  | |  | |  | |  | |
|  |  | |  | | |  | |  | |  | |  | |
|  | \|  \| \| --- \| | |  | | |  | |  | |  | |  | |
|  |  | |  | | |  | |  | |  | |  | |
|  |  | |  | | |  | |  | |  | |  | |
|  |  | |  | | |  | |  | |  | |  | |
|  |  | |  | | |  | |  | |  | |  | |
|  |  | |  | | |  | |  | |  | |  | |
|  |  | |  | | |  | |  | |  | |  | |
|  |  | |  | | |  | |  | |  | |  | |
|  |  | |  | | |  | |  | |  | |  | |
|  |  | |  | | |  | |  | |  | |  | |
|  |  | |  | | |  | |  | |  | |  | |
|  |  | |  | | |  | |  | |  | |  | |
| **Studies** |  | |  | | |  | |  | |  | |  | |
|  |  | |  | | |  | |  | |  | |  | |
| Ehrenbrusthoff et al. 2018 | No | Yes | | | Yes | | Yes | | Yes | | Due to the higher level of anxiety and depression, findings may not be generalizable to the wider CLBP population | |  |
| Nishigami et al. (2017) | Yes | Yes | | | Yes | | Yes | | Yes | |  | |  |
| Magni et al. (2018) | Yes | Yes | | | Yes | | Unc. | | Yes | |  | |  |
| Hirakawa et al. (2014) | No | Yes | | | Yes | | Yes | | Yes | |  | |  |
| Gilpin et al. (2015) | No | Yes | | | No | | Unc. | | No | |  | |  |
| Martinez et al. (2018) | No | Yes | | | Yes | | Unc. | | Yes | |  | |  |
| Treleaven et al. 2015 | Yes | Yes | | | Yes | | Unc. | | Unc. | |  | |  |
| Docherty et al. (2012) | No | Yes | | | Yes | | No | | Unc. | |  | |  |
| Grod. Et al (2002) | No | Yes | | | No | | Unc. | | Unc. | |  | |  |

*If control groups are absent: is there a correlation of the dysfunction detected with at least one out the two clinical variables of pain and disability

# Additional File 5 – Supplementary Table S4 - Evaluation of clinical relevance for treatment studies

|  | \|  \| \| --- \| |  |  |  |  |  |
| --- | --- | --- | --- | --- | --- | --- | --- |
|  |  |  |  |  |  |  |
|  |  |  |  |  |  |  |
|  |  |  |  |  |  |  |
|  |  |  |  |  |  |  |
|  |  |  |  |  |  |  |
|  |  |  |  |  |  |  |
|  |  |  |  |  |  |  |
|  |  |  |  |  |  |  |
|  |  |  |  |  |  |  |
|  |  |  |  |  |  |  |
|  |  |  |  |  |  |  |
|  |  |  |  |  |  |  |
| **Studies** |  |  |  |  |  |  |
|  |  |  |  |  |  |  |
| Wand et al. (2013) | Yes | Yes | Yes | No | Unc. | Disability was not considered as outcome measure and side effects were not reported |
| Louw et al. (2015) | No | Yes | No | Yes | Yes |  |
| Barker et al. (2008b) | No | no | Yes | no | Unc. | Significant faults were reported in 40% of the prototypes used at some point during the experimental procedure. It is not mentioned whether the point is stimulated again if the answer is incorrect |
| Wand et al. (2011) | No | Yes | Yes | yes | Yes | This study adopted a mixed type of intervention, with pain education, graded perceptual training program and motor training progression, thus it is not possible do discern the single component of the effect |
| Ryan et al. (2014) | No | Yes | yes | no | Yes | The need to involve a third person informal career, may constitute a potential barriers the practical execution of the treatment at home ad for the compliance |

*If control groups are absent: is there a correlation of the dysfunction detected with at least one out the two clinical variables of pain and disability?

|  | \|  \| \| --- \| | |  | |  | |  | |  | |  | |
| --- | --- | --- | --- | --- | --- | --- | --- | --- | --- | --- | --- | --- | --- |
|  |  | |  | |  | |  | |  | |  | |
|  |  | |  | |  | |  | |  | |  | |
|  |  | |  | |  | |  | |  | |  | |
|  |  | |  | |  | |  | |  | |  | |
|  |  | |  | |  | |  | |  | |  | |
|  |  | |  | |  | |  | |  | |  | |
|  |  | |  | |  | |  | |  | |  | |
|  |  | |  | |  | |  | |  | |  | |
|  |  | |  | |  | |  | |  | |  | |
|  |  | |  | |  | |  | |  | |  | |
|  |  | |  | |  | |  | |  | |  | |
| **Studies** |  | |  | |  | |  | |  | |  | |
|  |  | |  | |  | |  | |  | |  | |
| Morone et al. (2012) | no | yes | | Yes | | Yes | | Yes | | Costs for materials utilized for the treatment provided were not reported | |  |
| Paolucci et al. (2012) | No | Yes | | Yes | | Unc. | | Yes | | Costs for materials utilized for the treatment provided were not reported | |  |
| Vetrano et al. (2013) | No | Yes | | yes | | yes | | Yes | | Absence of a control placebo- or usual care group. Costs for materials utilized for the treatment provided were not reported | |  |
| Preston and Newport (2011) | no | Yes | | no | | Yes | | Unc. | | The experiment involved high-technological equipment | |  |
| Diers et al. (2013) | no | Yes | | no | | no | | Unc. | | The experiment involved high-technological equipment | |  |
| Stanton et al. (2018) | yes | Yes | | Yes | | Unc. | | Unc. | | The experiment involved high-technological equipment | |  |
| Nishigami et al. (2019) | No | Yes | | Yes | | Unc. | | Unc. | | The experiment involved high-technological equipment. Disability was not considered as outcome measure | |  |
| Lauche et al. (2012) | No | Yes | | No | | Unc. | | Unc. | | Authors not reported adverse events: cupping therapy can't be considered a non-invasive treatment, and it is not free of minor and major side effects | |  |

*If control groups are absent: is there a correlation of the dysfunction detected with at least one out the two clinical variables of pain and disability?

# Additional File 6 – Supplementary Table S5: Detailed features of included assessment studies

| **Study** | **Methods** | | **Condition** | **Participants** | | | | | | **Outcomes** | **Results** |
| --- | --- | --- | --- | --- | --- | --- | --- | --- | --- | --- | --- |
| Author (year) | a) Design  b) Country  c) Setting  d) Method of  Sampling  e) Control Group | | a) Clinical Condition  b) Major Eligibility Criteria (Patient)  c) Major Eligibility Criteria (Controls) | Patients  a) Mean Age (SD)  b) Female  c) Disease duration: Mean (SD)  d) Pain: mean (SD)  e) Disability: mean (SD) | | | Controls  a) Mean Age (SD)  b) Female  c) Disease duration: Mean (SD)  d) Pain: mean (SD)  e) Disability: mean (SD) | | | a) Pain  b) Disability  c) Body Perception  d) Other relevant outcomes  e) Follow-up | *Data presented as mean (SD) or MD (95% CI) unless otherwise indicated* |
| **Clinical Setting Studies** | | | | | | | | | | | |
| ***Implicit Somatoperception-Superficial Schema (Tactile Localisation Task)*** | | | | | | | | | | | |
| Wand et al. (2013) | a) Case-control Study  b) Australia  c) Clinical  d) Calculated a priori on the base of another experiment study (Wand et al. 2012) (1) (*n*=24)  e) Healthy controls (*n*=24) | | a) CLBP  b) Age:18-60 y, LBP as the main symptoms ≥ 6 m, modified item-7 of the SF-36 rated at least as ‘moderate’, no nerve root pain, no pregnancy status or <6 m post-partum, no any lumbar surgery or invasive procedure within the previous 12 m  c) no LBP in the past 6 m, no past episode of LBP sufficient to restrict work or leisure within the previous 2 y | a) 42 y (14.7)  b) 41.7 %  c) median= 5.5 y (IQR= 17.5)  d) 11-NRS: 45 (19)  e) RMDQ: 9.9 (5.6)  (+) for a) and b) between groups | | | a) 41.8 y (15)  b) 41.7 %  c) N.A.  d) N.R.  e) N.R. | | | a) 11-NRS (0-10)  b) RMDQ (0-24)  c) Localization task for tactile and painful stimuli (n° of mislocalizations). Subjects were asked to mark where they perceived tactile and painful stimuli on body chart with 12 pre-marked body areas of the trunk and thighs  d) 2-PDT (mm), TPT(g), PCS (0-52), TSK (17-68), PCS (0-52)  e) N.A. | 67% of subjects with CLBP reported at least 1 mislocalization with respect to 25% of controls (***p*=0.034**). Patients with CLBP reported maximum 5 mislocalizations on a possible total of 28  Correlation Analysis: no significant SD were found between mislocalizations errors and other variables |
| ***Model of Body Size and Shape, and Postural Schema (Body Size Perception and Tactile Localisation Task)- Implicit Somatoperception*** | | | | | | | | | | | |
| Adamczyk et al. (2018a) | a) Two-Case Report Study  b) N.R.  c) N.R.  d) Convenience sample (*n*=2)  e) N.A. | | a) CLBP  b) N.R.  c) N.R. | Subject A:  a) 26 y  b) Male  c) 6 m  d) 11-NRS: 4  e) ODI= 4% | | | Subject B:  a) 22 y  b) Female  c) 3 y  d) 11-NRS: 3  e) ODI= 24% | | | a) 11-NRS (0-10)  b) ODI (0-100%)  c) 2-PET (mm) for subject A and B; PTP test (mm) for subject A; qualitative version of the PTP test for subject B;  d) TPT (g)  e) N.A. | In patient A) 2-PET score was overestimated in the painful site compared to all non-painful locations: 64% (34.74 mm), 206% (59.74 mm) and 45% (27.42 mm) larger compared to the location above, below and the contralateral side respectively  Subjects B in contrast, underestimated the distance at the 2-PET: 22% (30.64 mm), 20% (27.59 mm) and 12% (14.9 mm) smaller than the location below, above, and the contralateral side respectively. PTP scores in P1 were overestimated at the painful site: 66% (18.67 mm), 84% (21.37 mm) and 24% (9.07 mm) compared to the location below, above and the contralateral side respectively. No difference in TPT between sides in both patients |
| ***Model of Body Size and Shape (Body Size Estimation) - Implicit Somatoperception*** | | | | | | | | | | | |
| Adamczyk et al. (2018b) | a) Preliminary Validation Study  b) Poland  c) Clinical  d) Calculated a priori (*n*=20)  e) N.A. | | a) CLBP  b) Age: >18 y, LBP > 6 m, LBP described an ongoing problem for more than 50% of the days in the previous 6m, dominantly unilateral- and not leg- related, no nerve root pain, no inability in detecting light touch  c) N.A. | a) 54.7 (14.3)  b) 67%  c) 224.4 (522)  d) 11-NRS: 3.6 (2.6)  e) ODI: 42(16) | | | N.A. | | | a) 11-NRS (0-10)  b) ODI (0-100%)  c) Two-Point Estimation (TPE) Task:  - manual: subjects have to estimate with a calliper a fixed-distance of 120 mm applied by the evaluator on the most painful area with a second calliper (%)  - verbal: the same procedure of the manual version but patients verbally estimate the two-point distance (%), FreBAQ (0-36)  d) Neuropathic Index Score (0-5); BDI (0-63), FPQ (0-150)  e) 10 minutes and 2 days | CLBP patients underestimated the calliper distance by 56.2% (manual version) and 45.9% (verbal version), irrespective of the examiner and location  Reliability: the manual version was more reliable than the verbal one  Inter-rater agreement: manual TPE (ICC= 0.75-0.91); verbal TPE (ICC: 0.53-0.88)  Stability of measure: ICC in the manual version of TPE reach stable values after two repetition: ICC=0.91 (0.77 to 0.97) and ICC=0.86 (0.65-0.94), respectively for pain-free and painful sides  SEM: manual version=4-8mm; verbal version= 8-14mm)  MDC: manual version= 9-16mm; verbal version= 19-33mm  Intra-examiner agreement:  - 10-minute interval (*n=21)*: ICC= 0.66-0.96 and ICC= 0.76-0.95, respectively for manual and verbal version  - Two-day interval (*n=21)*: ICC= 0.75-0.91 and ICC= 0.67-0.84, respectively for manual and verbal version  Regression Analysis: pain duration (β= -0.53, *p*<0.05) and pain intensity (β= -0.38, *p*<0.05) accounted for 42% of the total variance |
| ***Model of Body Size and Shape (Letter Recognition Task) - Implicit Somatoperception*** | | | | | | | | | | | |
| Wand et al. (2010) | a) Case-control Study  b) Australia  c) Experimental  d) Convenience sample (*n*=19)  e) Healthy controls (*n*=19) | | a) CLBP  b) Age: 20-55 y, symptoms >6 m, no nerve root pain, no pregnancy status or <6 m post-partum, no previous lumbar surgery  c) not having experienced any episode of LBP sufficient to restrict work or leisure within the previous 5 y, no pregnancy status or <6 m post-partum | a) 41 y (12.5)  b) 58%  c) 9.3 y (9.8)  d) 11-NRS usual= 3.9 (2.1); 11-NRS current= 3.2 (3); 11-NRS worst= 4.6 (2.9)  e) SF-36: 21.8 (5)  no significant SD for a) and b) between groups | | | a) 34 y (12.1)  b) 74%  c) N.A.  d) N.R.  e) N.R. | | | a) 11-NRS (0-10) as the usual, current and worst pain intensity  b) SF-36: item 3 - physical function (10-30)  c) Letter recognition error rate (n°)  d) TPT (g), 2-PDT (mm), HADS (0-42)  e) N.A. | No within-subjects side-to-side difference for TPT: 0* (95%CI= -0.04 to 0.04), (+)  2-PDT and Letter error rate were significantly larger in CLBP group: 2-PDT: 17.9 (5.9 to 29.8), (***p*=0.006**); letter recognition error rate: 6.1 (1.3 to 11), (***p*=0.016**)  Correlation Analysis:  no significant correlations were found between Letter error rate and 2-PDT in LBP group (raw data N.R.), nor between Letter error rate and any clinical data (pain duration, pain intensity, physical function and depression); *p*>0.094 |
| ***Depictive Methods (Body Image Drawings) - Explicit Somatoperception*** | | | | | | | | | | | |
| Moseley (2008) | | a) Exploratory case-control study  b) Australia  c) Clinical  d) Convenience sample (*n*=6)  e) Subjects with upper limb pain (*n*=10) | a) CLBP  b) LBP>12 m, ability to voluntarily tilt pelvis in the sagittal plane, in standing  c) upper limb pain, no history of LBP in the last 2 y | | a) 43.8 (11.1) median: 43 y (IQR: 20.8)  b) 50%  c) 52.2 m (36.1) (median: 40 (IQR: 43.3)  d) 101-VAS: 47.2 (12.5), median: 46.5 (IQR: 24)  e) N.R.  (+) for a) and b) between groups | | | a) N.R.  b) 50%  c) N.A.  d) N.R.  e) N.R. | | a) 101-VAS (0-100 mm) as the average pain of the previous week  b) N.R.  c) Clinical interview and Body Image Drawing of the trunk. Patients were asked to draw the outline of their own back they track it in their mind, without touching it  d) 2-PDT (mm), TPT(g)  e) N.A. | Qualitative analysis:  Body image drawings on control’s trunk were unremarkable. Five out of the six patients reported difficulties in delineating the full extent of their trunk: they verbatim refer that they “can’t find it”. Two subjects reported that ‘‘It feels as though it has shrunk”. No patients drew all vertebrae and missing vertebrae coincided with the level of the lost trunk delineation and of the usual pain. There was a tendency of vertebrae displacement from the midline in body drawings  Quantitative Analysis:  TPD was greater at the side and level of the missing outlines (>3SD) in 5 patients (single subjects’ raw data N.R.), even if overall 2-PDT values (mean=48.8, poled SD=3.5) were similar to the control (mean=47, SD=8)  TPT values were consistent across levels and sides in both groups |
| Lauche et al. (2012) | | a) Qualitative study embedded in a RCT (Lauche et al. 2012) (2)  b) Germany  c) N.R.  d) Convenience sample (*n=*6)  e) N.A. | a) CNP  b) N.R.  c) N.R. | | EG:  a) Pooled data of the whole sample considered in the RCT (Lauche et al. 2012) (2): 56 (9.5); range: 52-62 y  b) 83.3%  c) range: 4m-45 y  d) N.R.  e) N.R. | | | N.A. | | a) N.R.  b) N.R.  c) Themes and sub-themes emerged from interviews in which patients were asked to talk about their body image drawings (completed on day 0 and repeated 3 d later after the treatment).  c) Visual interpretation of the Body Image Drawing for neck and shoulders (modified version of that described by Moseley, 2008)  d) N.R.  e) N.A. | Interviews: patients refer changes in body perception of the neck as a feeling of swollen or distorted in proportion. These overestimations persist even when patients were aware of their actual appearance. Subjects in EG refer a reduction in neck size (smaller) as a relief from pain  Body Image Drawing: at the baseline the drawn body showed noticeable discrepancies respect to a “normal” body (missing lines and augmented dimension of shoulders and neck) in 4 out 6 subjects more symmetric and complete) |
| Mibu et al. (2015) | | a) Case-control study  b) Japan  c) N.R.  d) Method of sampling N.R. (*n=*20)  e) Healthy controls (*n*=20) | a) CNP  b) N.R.  c) N.R. | | *Normal Body Image sub-group (n=10)*  a) median=55.7 y (14);  b) 70%  c) 70.1 m (109.1)  d) 101-VAS: 51.1 (20.2)  e) N.R.  *Distorted Body Image sub-group (n=10)*  a) median=56 y (13)  b) 90%  c) 38.2 m (27)  d) 101-VAS: 48.6 (24.3)  e) N.R.  no significant SD for a), b), c) and d) within sub-groups and against healthy controls | | | a) 57.7 y (11.8)  b) 75%  c) N.A.  d) N.R.  e) N.R. | | a) 101-VAS (0-100 mm)  b) N.R.  c) Visual interpretation of the Body Image Drawing for neck and shoulders (modified version of that described by Moseley, 2008).  d) 2-PDT (mm), TSK (17-68), PCS (0-52)  e) N.A. | Body image is significantly (***p*= 0.0017)** distorted in neck pain patients (50%) than in healthy controls (5%)  2-PDT of the painful and non-painful side was significantly higher both in normal and distorted body image sub-groups (respectively 43.0 ± 17.4 and 33.5 ± 13.1; 42.5 ± 15.5; 40.5 ± 14.2) with respect to the healthy subjects (29.4 ± 9.7); ***p*= 0.034**  No significant difference for PCS and TSK between groups (respectively *p*= 0.587 and *p*= 0.941) |
| Nishigami et al. (2015) | | a) Case-control study  b) Japan  c) Clinical  d) Convenience sample (*n=*42)  e) Healthy-matched controls (*n*=17) | a) CLBP  b) Age:30-80 y, symptoms >6 m, without signs or symptoms of nerve root involvement, no previous lumbar surgery, BMI<30  c) no history of CLBP in the past 6 m, no diagnosed diseases | | *Normal" sub-group (n=18):*  a) 65.1 y (11.2)  b) 56.6%  c) 143 m (176.3)  d) 101-VAS: 48.3 (21.8)  e) RMDQ: 7 (4.2)  *"Expanded" sub-group (n=12):*  a) 56.7 y (16.7)  b) 66.7%  c) 63.5 m (64.6)  d) 101-VAS 42.5 (24.5)  e) RMDQ: 6.2 (3.4)  *"Shrink" sub-group (n=12):*  a) 62 y (12.4)  b) 66.7%  c) 64.7m (40.8)  d) 101-VAS: 42 (23.5)  e) RMDQ: 6.8 (4.4)  No significant SD for a) and b) within sub-groups and against healthy controls. No significant SD for c), d) and e) within sub-groups | | | a) 63.4 y (12.2)  b) 47%  c) N.A.  d) N.R.  e) N.R. | | a) 101-VAS (0-100 mm)  b) RMDQ (0-24)  c) Body Image Drawing of the trunk as described by Moseley (2008). Moreover, subjects were asked to judge the perceived image of their trunk as “normal”, “expanded” or “shrunken”  d) 2-PDT (mm), TPT(g), PCS (0-52)  e) N.A. | 42.8% of subjects with CLBP had a normal perceive image of the lower back, 28.5% had an expanded image, and 28.5% had a shrunken image. The 2-PDTwas larger for the expanded subgroup: 7.8 (1.83 to 13.66); ***p*< 0.05**, and normal subgroups: 8.8 (2.90 to 14.59); ***p*< 0.05**  There was no significant differences for VAS scores, pain duration, RMDQ, and PCS scores between three perceived image subgroups; *p*>0.127 |
| Moreira et al. (2017) | | a) Exploratory case-control Study  b) Portugal  c) N.R.  d) Convenience sample (*n*=7)  e) Healthy controls (*n*=7) | a) CNP  b) Age:>18 y, symptoms for at least 1/ w for the last 12 m or more, no history or neck/face trauma or surgery  b) no history of CNP, no history or neck/face trauma or surgery | | a) Pooled Mean= 37.6 y (N.R.)  b) 71%  c) 3.4 y (N.R.)  d) 2.1 (N.R.)  no significant SD for a) and b) between groups | | | a) Pooled Mean= 38.1 y (N.R.)  b) 71% | | a) 11-VAS (0-10 mm)  b) N.R.  c) Modified version of the Body Image Drawing of the trunk as described by Moseley (2008)  d) 2-PDT (mm), TPT(g)  e) N.A. | No difference between groups in TPT: 0.06 (-0.09 to 0.21), (*p*>0.05); as well as 2-PDT: -4.86 (-23.01 to 13.29); *p*>0.05 at any spine level  Qualitative analysis of the body image drawing: in both groups two subjects were not able to draw one side of the neck: comparing the drawings it seems that patients delineate neck and shoulders outline less symmetric and uniform than controls, and necks appear shorter. Moreover, two participants drew neck and shoulders more enlarged than they really were, and these perceptions coincided with pain location. Participants that not draw part of the neck, or drawing a clearly distorted neck tend to report pain of higher level and/or duration |
| ***Qualitative Studies - Explicit Somatoperception*** | | | | | | | | | | | |
| Valenzuela-Moguillansky (2013) | | a) Qualitative Study  b) N.R.  c) N.R.  d) Convenience sample (*n*=12)  e) N.A. | a) FM  b) NR  c) NR | | a) 55.4 y (NR)  b) 92%  c) median= 16.5; (IQR: 20)  d) N.R.  e) N.R. | | | N.A. | | a) N.R.  b) N.R.  c) Themes and sub-themes  d) N.R.  e) N.A. | Interviewees refer modifications in different aspects of body perception: body size, weight, localization and ownership. They talk about enlarged, thicker and heavy body parts. They also refer that near space is perceived as smaller, as if it was shrinking while their body become larger. At the peak of the pain stage some patients described the perception that the painful body parts did not belong to them (loss of the sense of body ownership), expressing the paradoxical experience of being in extreme pain while not feeling it. Moreover, they refer the inability to localize their painful body parts and pain |
| ***FreBAQ - Explicit Somatoperception*** | | | | | | | | | | | |
| Wand et al. (2014) | | a) Psychometric Validation Study  b) Australia  c) Clinical  d) Convenience sample (*n*=51)  e) Healthy controls (*n*=51) | a) CLBP  b) disease duration ≥6 m, modified item-7 of the SF-36 rated at least as ‘moderate’, no pregnancy status or <6 m post-partum, no any lumbar surgery or invasive procedure within the previous 12 m, no nerve root pain (clinicians' assessment)  c) no LBP in the last six-months, no LBP episode sufficient to restrict work or leisure within the last 2 y, current pregnancy status or <6 m post-partum | | | a) 41.7 y (14.0)  b) 41%  c) 8.2 y (10.4)  d) 101-VAS: 48.2 (17.8)  e) RMDQ: 10.1 (5.9)  (+) for a) and b) between groups | | | a) 38.7 y (13.4)  b) 39%  c) N.A.  d) N.R.  e) N.R. | a) 101-VAS (0-100 mm)  b) RMDQ (0-24)  c) FreBAQ (0-36)  d) PCS (0-52), TSK (17-68), HADS (0-42), DASS (0-84)  e) N.A. | Fifty of 51 (98%) CLBP patients endorsed some level of distortion in self-perception, with only one subject recording zero for all items  - Validity: FreBAQ mean total mean score in CLBP patients was 10.8 (range= 0–26), median score= 11; in healthy subjects was 0.5, median score=0 8 (range= 0–6); Median Difference: 11 (‡);  ***p*<0.001**  - Internal Consistency: Cronbach's alpha= 0.777 (internal consistent)  - 1w- Reliability (*n*=19): ICC_2,1_ for agreement= 0.65 (0.31to 0.85)  - Clinical Correlations with: pain duration [ρ = 0.357), pain intensity *[r*= 0.400], disability [*[r*= 0.365], and PCS [*r*= 0.408]: overall ***p*< 0.05**; TSK [*r*= 0.271]: *p*=0.054 |
| Wand et al. (2016) | | a) Cross-sectional Study  b) Australia  c) Clinical  d) Convenience sample (*n*=251)  e) N.A. | a) CLBP  b) Age: 18-70 y, disease duration >3 m, average pain intensity in the past week ≥ 2 on 11-NRS, ≥5 points on RMDQ, ≥60% score on specific question about the dominance of LBP versus leg pain, no previous extensive spinal surgery (greater than single-level fusion or discectomy) or any type of spinal surgery within the past 6 months, no acute vertebral fracture), no bilateral pain at the dorsum of the wrist/hand, no currently pregnant status  c) N.A. | | | a) 48.8 y (13.4)  b) 59%  c) median=120 m (IQR= 42-240)  d) 11-NRS: 5.8 (1.9)  e) RMDQ: median=9 (IQR= 6-13) | | | N.A. | a) 11-NRS (0-10)  b) RMDQ (0-24)  c) FreBAQ (0-36)  d) PCS (0-52), TSK (17-68), DASS (0-84), nociceptive sensitivity of the spine (pressure PT, heat PT, cold PT)  e) N.A. | FreBAQ mean total score= 9.8 (SD= 6.6); median score= 9.0 (IQR= 4.0–14.0  Correlation Analysis: bivariate association with disability (0.319; ***p*<0.001**), pain intensity (0.265; ***p*<0.001**), pain catastrophization (0.358; ***p*<0.001**), psychological distress (0.35; ***p*<0.376**), and lumbar pressure PT (-0.139; ***p*<0.001**) |
| Beales et al. (2016) | | a) Case-control questionnaire based study  b) Australia  c) Clinical  d) Convenience sample (*n*=24)  e) Women with no post-pregnancy pain (*n*=26) | a) Post-Partum LPP  b) LPP ≥3 m post-partum, no current pregnancy status  c) N.A. | | | *Low Disability sub-group** (n=12)*  a) median=35 y (IQR=32-36);  b) 100%  c) median=14 m (IQR=5.8-20.5)  d) Short-form MPGQ: 12.5 (range: 8.8-20.5)  e) ODI: median=8 (IQR=6-14)  *Moderate Disability sub-group** (n=12)*  a) median=35 y (IQR=32-37);  b) 100%  c) median=8.2 m (IQR=5.6-16.2)  d) Short-form MPGQ: 18.5 (range: 15.5 -27.8)  e) ODI: median=26 (IQR=22-35.5)  No significant SD for a) and b) within sub-groups and against healthy controls. Significant SD for c) between sub-groups and controls. No significant SD for d) between subgroups. Significant SD for e) between sub-groups | | | a) median= 32 y (IQR= 28-36);  b) 100%  c) N.A.  d) N.R.  e) N.R. | a) Short-form MGPQ (0-45)  b) ODI (0-100%)  c) FreKAQ (0-36)  d) PCS (0-52), TSK (17-68), DASS (0-126), Back Beliefs Questionnaire (9-63)  e) N.A. | FreBAQ median difference:  - 4.5 (‡) between Low Disability sub-group (6.5; IQR= 3.0 to 8.5) and pain free controls (2; IQR= 0 to 6); (‡), *p*=0.095 *(personal communication)*  - 1.5 (‡) between Low (6.5; IQR= 3.0 to 8.5) and Moderate Disability (8.0; IQR= 6.5 to 11) sub-groups; (‡); *p*=0.282 *(personal communication)*  - 6 (‡) between Moderate Disability sub-group (8.0; IQR= 6.5 to 11) and pain free controls (2; IQR= 0 to 6); (‡), ***p*=0.02**  - Clinical Correlations between TSK and FreBAQ=0.43; ***p*=0.04** *(personal communication)* |
| Wand et al. (2017) | | a) Exploratory cross-sectional questionnaire based study  b) Australia  c) General Population  d) Convenience sample (*n*=42)  e) N.A. | a) Pregnancy-related LPP  b) Age: 18-45 y, LPP within 3^rd^ trimester of pregnancy, no over 38^th^ week of pregnancy, not affected by psychiatric conditions, not taking any centrally acting medication, not having LPP sufficient to restrict work within 6^th^ month prior to the current pregnancy  c) N.A. | | | a) 29.0 y (4.9)  b) 100%  c) NR  d) 11-NRS: 3.9 (1.8)  e) PGQ: 37 (21.7) | | | N.A. | a) 11-NRS (0-10) as the average value of worst pain over the last w, average pain over the last week, and present pain  b) PGQ (0-100)  c) FreBAQ (0-36)  d) PCS (0-52), TSK (17-68), Back Beliefs Questionnaire (9-63), 2-PDT (mm), Laterality Recognition (ms, n° errors), Sensory profile: 11-NRS for pin prick pain and temporal summation  e) N.A. | FreBAQ median difference between pain (*n=8;* median total score=1; IQR: 0-1.5) and pain-free groups (*n=34*; median total score*= 3.5;* IQR: 2–8): 2.5; (‡); ***p*=0.005**  Correlation Analysis: association between FreBAQ and pain intensity (*r=*0.378; ***p*=0.027**) and catastrophization (0.403; ***p*=0.018**), but not with disability (*r=*0.256; *p*=0.143) |
| Nishigami et al. (2018) | | a) Psychometric Validation Study  b) Japan  c) Clinical  d) Calculated a priori (*n*=100)  e) N.A. | a) CLBP  b) inclusion: age: 20-80 y, LBP pain >3 m, no neurological signs  c) N.A. | | | a) 56 y (16.4)  b) 64%  c) 7.4 y (8.9)  d) 101-VAS on rest= 23.3 (23.6); 101-VAS on motion= 49.1 (27.1)  e) RMDQ: 6.3 (4.4) | | | N.A. | a) 101-VAS (0-100 mm) on rest and motion  b) RMDQ (0-24)  c) FreBAQ (0-36)  d) PCS (0-52), TSK (17-68), HADS (0-42)  e) N.A. | FreBAQ mean total score=11.7 (6.4)  - Internal consistency: person reliability= 0.76; Cronbach's alpha= 0.80 (good internal consistency)  - 2w-Reliability (*n*=40): ICC_3,1_ for agreement= 0.81 ( 0.67 to 0.89)  Correlation Analysis: the FreBAQ-J was significantly correlated with pain in motion (ρ= 0.25), RMDQ (ρ= 0.36), PCS (ρ= 0.38), TSK (ρ= 0.23), and anxiety (ρ= 0.19) (**overall** **ρ<0.05**) |
| Janssens et al. (2017) | | a) Psychometric Validation Study  b) Belgium (Dutch language)  c) Clinical  d) Convenience sample (*n*=73)  e) Healthy controls (*n*=73) | a) CLBP  b) age: 18-80 y, no acute LBP (<6 m)  c) ODI=0 | | | a) median=47 y (IQR= ±24)  b) 67%  c) NR  d) 11-NRS: median=4 (IQR= ±4)  e) ODI: median=22% (IQR= ±21) | | | a) median=47 (IQR= ±25)  b) 67%  c) N.A.  d) N.R.  e) N.R. | a) 11-NRS (0-10) on rest and motion  b) ODI (0-100%)  c) FreBAQ (0-36)  d) TSK (17-68)  e) N.A. | - Validity: FreBAQ mean total score was 11 (7) in LBP group (*n=*73*),* in control group (*n=*73*)* the median score was *3* (IQR= ±9); ***p*=0.001**  LBP subjects with ODI ≥20% (*n=*43) scored significantly higher on FreBAQ (13‡8) with respect to those with ODI <20% (*n=*30; 8‡6): MD= 5.0 (2.7 to 7.3); ***p*=0.005**. No significant SD between these two sub-groups for demographic variables  - 1w-Reliability in 48 LBP patients: ICC_2,1_ for agreement= 0.69 (0.51 to 0.82). SEM in LBP group= 3.9; MDC (95%)= 10.8 (30% of scale range), referring to a non-sufficient measurement error  Correlation Analysis: the FreBAQ was significantly correlated with ODI (rho=0.30; ***p*=0.010**) |
| Ehrenbrusthoff et al.  (2018) | | a) Psychometric Validation Study  b) Germany  c) Clinical  d) Convenience sample (*n*=35)  e) Healthy controls (*n*=48) | a) CLBP  b) age: ≥18 y, with or without leg pain, dominant back pain for patients with leg pain, symptoms duration ≥6 m, no pregnancy status or >6 m post-partum  c) no episode in the last 2 y restricting them from work or leisure activities, no significant spinal deformities | | | a) 52 y (15.2)  b) 65.8%  c) 11 y (11)  d) BPI-Pain Severity: 3.6 (2); BPI-Pain Interference: 2.6 (2.0)  e) RMDQ: 31.4 (9.8)  significant SD for a) between groups | | | a) 36 y (17.5)  b) 62.5%  c) N.A.  d) N.R.  e) N.R. | a) Short Form BPI: Pain Severity (0-10), Pain Interference (0-7)  b) RMDQ (0-24)  c) FreBAQ (0-36)  d) HADS (0-42), EurQuol 5D-3L: 101-VAS (0-100) and Index Value (0-1); 2-PDT (mm)  e) N.A. | FreBAQ mean total score= 8.8 (6.1) for assessor 1 and 7.8 (7.0) for assessor 2 in LBP group (*n=*35*)*  Validity: Global FreBAQ mean total score: 8.8 (6.1) in LBP group (*n=*35*)* and 4.0 (3.3) in control group (*n=*48*)*; ***p*=0.001.** Unadjusted MD= 4.8 (2.6 to 7.2); ***p*<0.01**. MD adjusted for Age, Gender and BMI= 5.4 (3.0 to 7.8); ***p*<0.01**  - 1w-Reliability: ICC_2,1_ for absolute agreement= 0.88 (95% CI: 0.77-0.94). SEM= 1.80-2.92  - Inter-observer reliability: ICC3.1 for absolute agreement 0.88 (95%CI: 0.75 to 0.94). SEM= 1.74-2.82  - Convergent Validity: FreBAQ was not associated with 2-PDT in patient group (*rho*= -0.05; p =0.79)  Correlation Analysis: the FreBAQ was significantly correlated with HADS-Anxiety (*r=*0.37; ***p*=0.03**); with HADS-Depression (*rs=*0.52; ***p*<0.001**), BPI-Pain Interference (*rs=*0.47; ***p*<0.001**) and RMDQ (*rs=*0.46; ***p*<0.001**) |
| ***FreKAQ - Explicit Somatoperception*** | | | | | | | | | | | |
| Nishigami et al. (2017) | | a) Psychometric Validation Study  b) Japan  c) Clinical  d) Convenience sample (*n*=65)  e) Healthy subjects (*n*=65) | a) Knee OA  b) ACR Criteria (1986), knee pain for *>*3 m, Kellgren-Lawrence (KL) score ≥ 2 and age between 40 and 85 y, no total knee arthroplasty, no neurological findings  c) N.R. | | | a) 68.5 y (9.1)  b) 76.9%  c) 57.7 m (88.4)  d) 101-VAS on Rest: 19.6 (21.7). 101-VAS on Motion 43.5 (24.1)  e) OKSQ: 31.4 (9.8)  no significant SD for a), b), c) d) and e) between groups | | | a) 66.7 y (7.2)  b) 76.9%  c) N.A.  d) N.R.  e) N.R. | a) 101-VAS (0-100 mm) during rest and motion  b) OKSQ (0-48)  c) FreKAQ (0-36)  d) PCS (0-52), TSK (17-68), HADS (0-42)  e) N.A. | FreKAQ mean total score= 12.4 (SD= 7.6) in knee patients vs. 3.4 (4.4) in healthy controls  - Validity: median difference between groups= 9.0, (6.7 to 11.1; (***p*= 0.001**)  - Internal Consistency: person reliability 0.81; Chronbach’s alpha 0.88 (internal consistent)  - 2w- Reliability (*n*=23): ICC_3,1_ for agreement= 0.76 (0.52 to 0.89)  Correlations with: pain during motion (rho= 0.37; ***p*=0.002**), OKSQ (rho= -0.41; ***p=*0.001**), PCS (rho= 0.70; ***p*<0.001**), TSK (rho=0.49*;* ***p*<0.001**) and HADS (rho=0.46; ***p*<0.001**), but not with pain duration (rho= -0.06; *p*= 0.76) |
| ***Neglect-like Symptoms Questionnaire - Explicit Somatoperception*** | | | | | | | | | | | |
| Magni et al. (2018) | | a) Case-Control Study  b) New Zeeland  c) Clinical  d) Convenience sample (*n*=20)  e) Healthy subjects (*n*=19) | a) Hand OA  b) ACR Criteria (1990), radiographic evidence of Kellgren-Lawrence (KL) score >1, no symptoms of upper limb radiculopathy  c) no upper limb pain or symptoms of upper limb radiculopathy, no Cervical/thoracic pain | | | a) 71.7 y (6.9)  b) 75%  c) 14.7 y (13)  d) 11-NRS: 4.6 (2)  e) DASH: N.R.  (+) for a), b) c), and d.  Authors declared significant SD for e) between groups | | | a) 70.5 y (7.7)  b) 74%  c) N.A.  d) N.R.  e) N.R. | a) 11-NRS (0-10) as a mean pain during the previous week  b) DASH (0-100)  c) NLSQ (5-30)  d) 2-PDT (mm), Laterality Recognition (ms, n° errors), TEMPA (s), Purdue Pegboard Test (n°/30s; n°/1m)  e) N.A. | The hand OA group reported neglect-like symptoms (median score : 5.5; IQR: 3) significantly more often than the control group (median score: 5; IQR: 0): 0.5 (‡); χ^2^(1)=12.8, ***p*<0.001**,Cramer’s V =0.6 |
| Hirakawa et al. (2014) | | a) Longitudinal Study  b) Japan  c) Clinical  d) convenience sample (*n*=90)  e) N.A | a) Total Knee Arthroplasty for Knee OA  b) Total knee arthroplasty performed for Knee OA, no complications such as nerve injury and deep vein thrombosis, no previous Total Knee Arthroplasty (at the opposite limb and revision), no Arthroplasty performed for causes other than degenerative diseases (rheumatoid arthritis and bone necrosis)  c) N.A. | | | a) 76 y (6.3);  b) 78%  c) N.R.  d) 101-VAS: 37.5 (19.9)  e) N.R. | | | N.A. | a) 101-VAS (0-100 mm) as the most severe pain during post-operative active maximum flexion and extension of the knee  b) N.R.  c) NLSQ (0-500): Motor Neglect (MN) and Cognitive Neglect (CN) sub-scales  d) PCS (0-52), STAI (20-80), Muscle strength (maximum isometric force of the knee extensor at 60° of flexion [kg]), Active ROM (°), JPS (°), 2-PDT (mm)  e) 3 w and 6 w postoperatively | The percentage of patients with a total NLSQ ≥100 was 36% (MN, 40%; CN, 18%) at 3 w and 19% (MN, 19%; CN, 5%) at 6w  The total score of NLSQ (for both MN, and CN sub-scales) decreased at 6 w: total score from 77.7 (87) to 41.2 (62.1); Cronbach’s α ≥0.92 for the total score, however, the SD was high, indicating a large variation among patients  Correlation Analysis:  - NLS-MN was associated with Pain at 3 w (β=0.50; ***p*<0.01**) and 6 w β=0.53; ***p*<0.01**)  - In regression analysis the NLSQ-MN score was associated with JPS at 3w (β=0.23; ***p*<0.05**), and with ROM (β= -0.28; ***p*<0.01**) and JPS (β= 0.39; ***p*<0.01**) at 6 w |
| **Experimental Setting Studies** | | | | | | | | | | | |
| ***Visual Estimation Task - Explicit Somatoperception*** | | | | | | | | | | | |
| Gilpin et al. (2015) | | a) Case-control Study  b) Great Britain  c) Experimental  d) Convenience sample (*n*=12)  e) Healthy controls (*n*=12) | a) Hand OA  b) Diagnosis of Hand OA ≥6 m, major surgical procedures that may have damaged peripheral nerve pathways, no previous exposure to illusions in author‘s laboratory  c) Pain free in the hands and upper limb | | | a) 60.1 y (range= 53-75)  b) 67%  c) N.R.  d) N.R.  e) N.R.  (+) for a) and b) between groups | | | a) 59 y (range= 52-67)  b) 50%  c) N.A.  d) N.R.  e) N.R. | a) N.R.  b) N.R.  c) Visual size estimation task (% of the real hand size). Participants judge a snapshots image of their hand that were manipulated in length (from 67% to 150% of the real dimension) using the MIRAGE system, until subjects identified that it looked the right size to be their real hand  d) N.A.  e) N.A. | Hand size estimations were significantly smaller for the OA group: -8.01 (3.07 to 12.94); t(22) = 2.39, ***p*= 0.026,** indicating an underestimation of hand dimensions |
| ***RHIP -* *Body Ownership*** | | | | | | | | | | | |
| Martinez et al. (2018) | | a) Case-control Study  b) Spain  c) Experimental  d) Convenience sample (*n*=14)  e) Healthy controls (*n*=13) | a) FM  b) N.R.  c) N.R. | | | a) 54.4 y SEM=1.9  b) 100%  c) N.R.  d) 11-VAS= 8.9 (SEM=0.3); BPI= 7.9 (SEM=0.4)  e) FIQ: 88(2.7)  No significant SD for a) between groups. Significant SD for d) between groups. (+) for b) | | | a) 53.9 y; SEM=3.3  b) 41.7 %  c) N.A.  d) VAS= 0.2 (SEM=0.1); BPI= 0.2 (SEM=0.1)  e) N.R. | a) 11-VAS (0-10 mm), Short-form BPI (0-20)  b) FIQ (0-100)  c) 5-point Likert Scale measuring proprioceptive drift (0.35), ownership (0-35) and agency (0-30)  d) SF-12: physical and mental component, BPI (1-20)  e) N.A. | FM patients were more prone to experiment the misperceptions produced by the RHIP. They scored significantly (***p*<0.05***)* higher in all 5-items of the proprioceptive drift scale and in 4 out 5 items of the agency scale (Effect Size varying between 0.88 to 3.10): differences were largest in the proprioceptive drift domain, where large effect sizes were found across all items |
| ***Perception of subjective visual vertical/ horizontal - Extra-personal Space Perception*** | | | | | | | | | | | |
| Treleaven et al. 2015 | | a) Case-control Study  b) Australia  c) Experimental  d) Convenience sample (*n*=36 with CNP; *n*=42 with WAD)  e) Healthy controls (*n*=48) | CNP  a) Age: 18-60 y, symptoms duration ≥3 m, NDI ≥10%, no history of head/neck/upper limb pain or trauma during the previous 3 m  WAD  a) classification of WAD II according to the Quebec Task Force Classification, no neck trauma associated with post-traumatic amnesia or concurrent head injury, no cervical fracture/dislocation, no known vestibular pathology  b) no limited daily activities neck or upper thoracic pain or for which subjects had sought care in the previous month.  For all groups: no neck trauma associated with post-traumatic amnesia or concurrent head injury, no cervical fracture/dislocation, no known vestibular pathology | | | CNP group:  a) 32.7 y (13.8)  b) 55%  c) N.R.  d) N.R.  e) NDI: 21.5% (8.4), Short Form DHI: 9.9 (2.5)  WAD group:  a) 34.3 y (10.8)  b) 64%  c) N.R.  d) N.R.  e) NDI: 39.9% (15.2), Short Form DHI: 7.5 (3.4)  Significant SD in a) between Healthy Controls and WAD, in e) Short Form DHI between all groups and in e) NDI between CNP and WAD groups | | | Healthy Controls group:  a) 29.4 y (10.8)  b) 72%  c) N.A.  d) N.A.  e) NDI: N/A; Short Form DHI: 12.6 (1.1) | a) N.R.  b) NDI (0-100%), Short Form DHI (0-13)  c) SVV: Computerized Rod And Frame (CRAF) test as described by Takasaki et al. (2012)(3): subjects were asked to rotate (0.01° increments) two dots (representing each end of the rod) shown on a shown on video glasses, using the computer's mouse until reaching the perceived position that were judged as vertical. The same task was required also with a tilted (18° clockwise or anticlockwise) surrounding frame.  Error calculation: mean AE (°), mean VE (°), mean CE (°), mean RMSE (°)  d) N.R.  e) N.A. | CNP group had significantly larger variability of SVV errors between the three groups: 1) VE= 0.5 (0.23 to 0.77) vs. healthy controls (***p*=0.001**); 0.37 (0.07 to 0.67) vs. WAD (***p*=0.02**); 2) RMSE= 0.51 (0.09 to 0.93) vs. healthy controls (***p*=0.01**); 0.58 (0.20 to 0.96) vs. WAD (***p*=0.01**)  No significant SD for AE and DE between groups (*p*-values respectively of 0.06 and 0.99)  Despite the higher level of disability of the WAD group, there were no significant differences in SVV error between this group and healthy subjects (*p*=0.91)  SVV errors and Disability (DHI) seemed to be unrelated. |
| Docherty et al. (2012) | | a) Case-control Study  b) Swiss  c) Experimental  d) Convenience sample (*n*=50)  e) Age and gender healthy paired controls (*n*=50) | CNP  a) Age: 30-65 y, CNP with or without arm pain for at least 4 w, no recent history of cervical spine surgery  b) no neck or upper thoracic pain that had limited daily activities or for which subjects had sought care in the previous month | | | a) 48.1 y (8.7)  b) 80%  c) 80.3% had pain >1 y  d) NRS: 4.5 (N.R.)  e) NDI: 33 (16)  no significant SD for a) and b) between groups | | | a) 47.9 y (8.7)  b) 80%  c) N.A.  d) N.A.  e) N.A. | a) 11-NRS (0-10)  b) NDI (0-50)  c) SVV and SVH: Computerized Rod And Frame (CRAF) test (°): subjects were asked to rotate (0.5° increments) the dots shown on video glasses using right and left buttons of the mouse until reaching the perceived position that were judged as vertical or horizontal. The same task were required also with a tilted surrounding frame  d) N.R.  e) N.A. | In absence of surrounding frame, significant difference were found in mean errors *(****p*<0.05**) both for SVV and SVO test between groups, however they fell within a range considered normal (<0.5°)  Untilted surrounding reference of frame does not produce significant error with respect to no surrounding frame conditions (*p*>0.05), while tilting the frame clockwise or counter clockwise by 18° resulted in significant between-groups difference both for the SVV and SVO (***p*< 0.001** in all cases), although the difference between the medians for these tests were still small (less than 2°)  Of the 50 CNP patients, a subgroup of 8 subjects (16%) exhibited higher than normal errors in both the SVV and SVO: these patients scored higher on the NDI than patients whose errors fell within the reference range (*U*=74.0, *p*<0.016)  Correlation Analysis:  There was a significant positive correlation between the results for the horizontal and vertical tests in both CNP patients (*rho*=0.733, ***p*<0.001**) and controls (*rho*=0.656, ***p*<0.001**) |
| Grod et al. (2002) | | a) Cohort study  b) Canada  c) Experimental  d) Convenience sample (*n*=19)  e) Age and gender paired sample without neck pain (*n*=17) | Acute or recurrent NP  a) no recent history of cervical spine surgery, blindness, or serious visual impairment  b) LBP or peripheral joint complaints but no current neck pain | | | a) 38.5 y (N.R.)  b) 58%  c) N.R.  d) N.R.  e) N.R. | | | a) 38.6 y (N.R.)  b) 41%  c) N.R.  d) N.R.  e) N.R. | a) N.R.  b) N.R.  c) Computerized Rod And Frame (CRAF) test as used by Docherty et al. (2012). SVV (°) and SVH (°)  d) N.R.  e) N.A. | Statistically significant differences in SVV and SVH were found between symptomatic and asymptomatic subjects (F=13.37, p= **0.001**); pooled Mean Difference= 1.99° (pooled SD=1.61) |

**Abbreviations:** ACR: American College of Rheumatology; CLBP: Chronic Low Back Pain; LPP: Lumbo-Pelvic Pain; FM: Fibromyalgia; OA: Osteoarthritis; NP: Neck Pain; CNP: Chronic Neck Pain; VAS: Visual Analog Scale; NRS: Numeric Rating Scale; Chronic Pain Grade: CPG; BPI: Brief Pain Inventory; MGPQ: McGill Pain Questionnaire; ODI: Oswestry Disability Index; RMDQ: Roland Morris Disability Questionnaire; DASH: Disabilities of the Arm, Shoulder and Hand Questionnaire; PGQ: Pelvic Girdle Questionnaire; TSK: Tampa Scale of Kinesiophobia; PCS: Pain Catastrophizing Scale; FPQ: Fear of Pain Questionnaire; HADS: Hospital Anxiety and Depression Scale; BDI: Beck Depression Inventory; DASS: Depression Anxiety Stress Scale; STAI: State-Trait Anxiety Inventory; FIQ: Fibromyalgia Impact Questionnaire; SIQ: Symptoms Impact Questionnaire; OKSQ: Oxford Knee Score Questionnaire; MAIA: The Multidimensional Assessment of Interceptive Awareness; DSM-IV: Diagnostic and Statistical Manual of Mental Disorders IV; PU: Pain Unpleasantness; PI: Pain Intensity; PeT: Perception Threshold; PT: Pain Threshold; PTo: Pain Tolerance; FreKAQ: Fremantle Knee Awareness Questionnaire; NLSQ: Neglect-like symptoms questionnaire, TPT: Tactile Perception Threshold; 2PDT: 2-Point Discrimination Threshold; PTP: Poin-to-Poin Test; 2-PET: two Point Estimation Test; ROM: Range Of Motion; JPS: Joint Position Sense; TEMPA: Test d'Evaluation de la Performance des Membres Supérieurs des Personnes Agées; BMI: Body Mass Index; RHIP: Rubber Hand Illusion Paradigm; SVH: Subjective Visual Horizontal; SVV: Subjective Visual Vertical; AE: Absolute Error; DE: Direction of Error: RMQE: Root Mean Square Error; MDC: Minimal Detectable Change; ICC: Interclass Correlation Coefficients; MD: Mean Difference; NR: not reported; NA: Not Applicable; SD: Standard Deviation; IQR: Interquartile Range; SEM: Standard Error of Measurement; CI95%: Confidence Interval; +: *p-value* not reported; SD: statistical difference; y: year/s; m: month/s; h: hour/s; min.: minute; s: second/s; ms: millisecond. ***p-values*** are reported in bold if statistically significant; ** sub-groups were obtained through the median split with established ODI categorisation.

**Refererences:**

1. Wand BM, Tulloch VM, George PJ, Smith AJ, Goucke R, O’Connell NE, et al. Seeing it helps: movement-related back pain is reduced by visualization of the back during movement. *Clin J Pain*. 2012 Sep;28(7):602–8.

2. Lauche R, Cramer H, Hohmann C, Choi K-E, Rampp T, Saha FJ, et al. The effect of traditional cupping on pain and mechanical thresholds in patients with chronic nonspecific neck pain: a randomised controlled pilot study. *Evid Based Complement Alternat Med*. 2012;2012:429718.

3. Takasaki H, Treleaven J, Johnston V, Jull G. Minimum repetitions for stable measures of visual dependency using the dot version of the computer-based Rod-Frame test. *Man Ther* 2012b;17(5):466e9.

# Additional File 7 – Supplementary Table S6: Detailed features of included intervention studies

| **Study** | **Methods** | **Condition** | **Participants** | | **Core Outcomes** | **Interventions** | **Results** | |
| --- | --- | --- | --- | --- | --- | --- | --- | --- |
| **Author (year)** | **a) Design**  **b) Country**  **c) Setting**  **d) Method of Sampling**  **e) Sample size**  **f) Control group**  **g) Drop-out** | **a) Clinical condition**  **b) Inclusion criteria**  **c) Exclusion criteria** | **Demographic Data**  **a) Mean Age (SD)**  **b) Female (%)**  **c) Disease duration: Mean (SD)** | **Baseline values**  **a) Pain Severity: Mean (SD)**  **b) Disability: Mean (SD)**  **c) Other relevant values: Mean (SD)** | **a) Pain**  **b) Disability**  **c) Body Perception**  **d) Other relevant outcomes**  **e) Follow-up** | **a) Experimental group**  **b) Control group/s** | **a) Group Pre/post change: MD (95% CI)**  **b) Between-groups**  **comparison: MD (95% CI)** | |
| ***Tactile Localization Training*** | | | | | | | | |
| Wand et al. (2013) | a) Randomized COT  b) Australia  c) Clinical  d) Calculated a priori (*n*= 25)  e) *n*=25  f) N.A.  g) *n*=0 | a) CLBP  b) age: 18-60 y , disease duration ≥ 6 m, item-7 SF-36 rated as "moderate"  c) >6m post-partum, spinal surgery previous 24 m, nerve root pain | a) 41.5 y (13.8)  b) 36%  c) 6.7 y (11.5) | a) NRS: 4.9 (1.5)  b) RMDQ: 9.6 (5.9) | a) 11-NRS (0-10) after performing 10 repeated spine movement in the most provocative direction reported in the initial physical examination  b) N.R.  c) N.R.  d) N.R.  e) after treatment | a) Acupuncture involving sensory discrimination training: single session  b) Acupuncture: single session | a) 11-NRS: -0.9 (-0.3 to –1.5); ***p*=0.008,** regardless of treatment order  b) 11-NRS: -0.8 (-1.4 to -0.3) in favour to EG; *p*=**0.011**  n.b. no significant period/treatment interaction (*p*=0.182) | |
| Louw et al. (2015) | a) Case-series  b) N.R.  c) Clinical  d) Convenience sample  e) *n*=16  f) N.A.  g) *n*=0 | a) CLBP  b) age: > 18y  c) previous spinal surgery, leg pain only, neurological deficit only in the lower extremities | a) 48.2 y; range: 20.7-71.7  b) 75%  c) 11.9; range: 6 m-30 y | a) NRS: 5.6 (NR)  b) ODI: 34.4 (NR) | a) 11-NRS (0-10)  b) N.R,  c) N.R.  d) Functionality: active lumbar flexion (cm), FABQ (0-92)  e) after treatment | a) Localization task: 5 min., single session  b) N.A. | a)  - 11-NRS: **-1.9**; (‡) range: 0 to 6; +  - Functionality **4.8**; (‡) range: -1 to 21; + | |
| Barker et al. (2008) | a) Single-blinded, RCNIT  b) United Kingdom  c) Clinical  d) Calculated a priori (*n*= 25)  e) *n*=60 (EG: *n*=32)  f) *n*=28  g) *n*=6 (1 in EG and 5 in CG) | a) CLBP  b) age: >18 y; disease duration >3 m  c) leg pain, current TENS users, poorly enervated areas | EG:  a) 52.7 y (10.7)  b) 50%  c) N.R.  CG:  a) 54.1 y (12.5)  b) 50%  c) N.R.  (+) for difference in a), b) and c) between groups | EG:  a) VAS: 6.3 (1.9)  b) ODI: 40.8 (15.9);  c) Functioning:  - 5’-WD: 235.6 metres/5 min. (103.2)  - 1’-SC: 6.5 n°/min. (2.7)  - 1’-STS: 13.7 n°/min (7.6)  CG:  a) VAS: 6.6 (1.4)  b) ODI: 42.8 (14.8);  c) Functioning:  - 5’-WD: 219.6 (111.7)  - 1’-SC: 6.7 (3.0)  - 1’-STS: 12.7 (6.7)  (+) for difference in a), b) and c) between groups | a) 0-11 VAS (0-10 mm) as a mean of patients’ present pain intensity level, their average and worst pain intensity levels recorded over a week  b) ODI (0-100%)  c) Physical Functioning: 5 minute walking distance (5’-WD), 1 minute stair climb (1’-SC) and 1 minute sit-to—stand (1’-STS);  e) After 3 weeks | a) FairMed (device for sensory discrimination training): 30 minutes, twice a day, for 3 weeks  b) TENS: 30 minutes, twice a day, for 3 weeks | a)  EG:  - 0-11 VAS: -0.8 (-1.5 to –0.1); *p*=0,83  - ODI: -0.6 (-3.8 to 2.7) *p*=0.85  - Functioning:  5’-WD: 3.1 (-12.6 to 18.9) *p*=0.58  1’-SC: 0.4 (-0.4 to 1.1) *p*=0.81  1’-STS: 1.0 (0.1 to 1.8) *p*=0.90  CG: -0-11 VAS: **-7.3** (-8.1 to -6.6); *p*=0.83  - ODI: -0.9 (-3 to -1,1) *p*=0.85  Functioning:  - 5’-WD: -9.1 (-6.4 to 24.6) *p*=0.58 - 1’-SC: 0.5 (0.1 to 0.9) *p*=0.81 - 1’-STS: 1.0 (0.1 to 1.9) *p*=0.90  b)  - 0-11 VAS: -0.1 (-0.7 to 0.3); *p*=0.82  - ODI: 0.4 (-0.7 to 0.4); *p*=0.85  - 5’-WD: -6 (-0.3 to 0.7); *p*=0.58  - 1’-SC: -0.1 (-0.6 to 0.4); *p*=0.81  - 1’-STS: 0 (-0.4 to 0.6); *p*=0.90 | |
| ***Mixed perceptual training (graded perceptual training + graded motor retraining)*** | | | | | | | | |
| Wand et al. (2011) | a) Three single-case study  b) Australia  c) Clinical  d) Convenience sample  e) *n*=3  f) N.A.  g) *n*=0 | a) CLBP  b) age: 18-60 y, disease duration >12 m, RMDQ score >4, item-7 SF-36 rated as "moderate"  c) >6m post-partum, spinal surgery <24 m, nerve root pain | a) 39 y (14); median: 33 y; IQR: 31-44  b) 66%  c) 60.7 y (54.1); median: 48; IQR: 31-84 | a) VAS: 5 (1.6)  b) RMDQ: 10.9 (4.4) | a) 11-NRS (0 – 10)  b) RMDQ (0-24)  e) weekly for 10 weeks during treatment, weekly for 1 month after the end of treatment  c) N.R.  d) N.R, | a) Education plus graded perceptual retraining program (localization and graphaestesia training) combined with graded motor retraining: minimum 10 w of home exercise program and encouragement to increase physical activity level | | a)  - 11-NRS: **-2.9**; (1.2 to 4.6) at T1; ***p*<0.001**; **-3.9**; (1.6 to 6.2) at T2; ***p*<0.001**  - RMDQ: **-5.2** (2.4 to 8) at T1; ***p*<0.001**; **-9.6** (4.2 to 15) at T2; ***p*<0.001** |
| Ryan (2014) | a) Mixed-methods pilot RCT  b) UK  c) Clinical  d) Convenience sample  e) EG: *n*=12  f) *n*=12)  g) *n*=9 (EG: *n=*3; CG: *n=6)* | a) CLBP  b) ≥18 y, non-specific CLBP with or without leg pain, pain duration $\geq$6 m, no peripheral neuropathy  c) Not having an informal carer | EG:  a) 45 y (17)  b) N.R.  c) 10.4 y (13.5)  CG:  a) 46 y (14)  b) N.R.  c) 8.7 y (11.4)  no statistical SD for a) and c) between groups | EG:  a) 101-VAS: 49 (19)  b) RMDQ: 9.3 (6.6)  c) 61 mm (16)  CG:  a) 101-VAS: 48 (31)  b) RMDQ: 7.3 (3.1)  c) 57 mm (16)  no statistical SD for a), b) and c) between groups | a) 101-VAS (0-100 mm)  b) RMDQ (0-24)  c) N.R.  d) 2-PDT (mm), Themes and sub-themes on N.R.  attitudes toward the treatment  N.R.  e) after treatment | a) tactile acuity training and graphaestesia acuity training as used by Wand et al., 2011 (2) plus usual physiotherapy, 3 sessions + 21 at home  b) tactile stimulation alone (placebo) plus usual, 3 formal sessions + 21 at home | | a) EG: -101-VAS: -8 (-28 to12); *p*>0.05  - RMDQ: **-1.6** (-4.4 to1); *p*>0.05  CG : -101-VAS: **-33.2** (-58.3 to -8); ***p*<0.05**  - RMDQ: **-4** (-6.7 to -1.3); ***p*<0.05**  b)  - 101-VAS: 25.6 (-0.7 to 51.9), (*p*=0.056);  - RMDQ (0-24): 2.2 (-1.6 to 6.0), (*p*=0.237) |
| ***Mixed perceptual training SuPerR Treatment*** | | | | | | | | |
| Morone et al. (2011) | a) Single-blinded, RCT  b) Italy  c) Clinical  d) Convenience sample (*n*=75)  e) EG: *n*=25  f) CG 1: *n*=25; CG 2: *n*=25  g) *n*=5 (5 in EG) | a) CLBP  b) age 18-75 y, disease duration ≥3 m  c) acute LBP, scheduled back surgery, pregnancy, leg pain only | EG:  a) 52.7 y (17.6)  b) 84%  c) NR  CG 1:  a) 55.4 y (13.7)  b) 64%  c) NR  CG 1:  a) 57.9 (12.8)  b) 68%  c) NR  not statistical SD for a) in all three groups  (+) for difference in b) between groups | EG:  a) VAS: 6 (1)  b) ODI: 34 (20)  CG 1:  a) VAS: 7 (2)  b) ODI: 26 (24)  CG 2:  a) VAS: 7 (2)  b) ODI: 24 (20)  (+) for difference in a) and b) between groups | a) 11-VAS (0-10 mm)  b) ODI (0-100)  c) N.R.  d) N.R.  e) post-treatment (T1), at 12 w (T2) and 24 w (T3). | a) SuPeR: perceptive rehabilitation protocol with perceptive surface plus active exercises (45’, 3 x week for 1 month) and usual care (medical and pharmacological assistance)  b)  - Back school program with: 1 initial theory session and 9 practical sessions of 45’ in 1 month, plus usual care (medical and pharmacological assistance)  - Medical and pharmacological assistance only (as the other two groups) | | a)  EG:  - 11-VAS: **-2***(‡) at T1, -1*(‡) at T2 and -1*(‡) at T3 (***p*<0.001**)  - ODI: **-18*** (‡) at T1, **-18*** (‡) at T2 and **-14*** (‡) at T3 (***p*<0.001**) (‡)  CG1:  - 11-VAS: **-**1*(‡) at T1, -2*(‡) at T2 and **-3***(‡) at T3 (***p*<0.001**)  - ODI: -10***** (‡) at T1, **-14*** (‡) at T2 and **-16*** (‡) at T3 (***p*<0.001**) (‡)  CG2:  - 11-VAS: 1*(‡) at T1, 1*(‡) at T2 and 0*(‡) at T3 (***p*=0.028**)  - ODI: -2* (‡) at T1, **-**2* (‡) at T2 and 2***** (‡) at T3 (*p*=0.734) (‡)  b)  - 11-VAS: -1* (‡) vs. CG 1 and **-3*** (‡) vs. CG 2 at T1 (***p<*0.001**); 1* (‡) vs. CG 1 and -2* (‡) CG 2 at T2 (***p<*0.001**); 2* (‡) vs. CG 1 and -1* (‡) CG 2 at T3 (***p*=0.009**)  - ODI: -8* (‡) vs. CG 1 and **-16*** (‡) vs. CG 2 at T1 (p=0.403); -**14*** (‡) vs. CG 1 (p=0.065) and **-20*** (‡) vs. CG 2 at T2 (**p<0.009**); 2* (‡) vs. CG 1 (p=0.169); and **-16*** (‡) vs. CG 2 at T3 (**p=0.023**) |
| Paolucci et al. (2012) | a) Single-blinded, RCT  b) Italy  c) Clinical  d) Convenience sample (*n*=30)  e) EG: *n*=15  f) *n*=15)  g) N.R. | a) CLBP  b) age 18-75 y, duration >3 m  c) acute LBP, scheduled back surgery | EG:  a) 55.4 y (16.4)  b) NR  c) NR  CG:  a) 62.1 y (10.6)  b) NR  c) NR  (+) for difference in a) between groups | EG:  a) MGPQ: 40 (15)  CG:  a) MGPQ: 51 (31)  (+) for difference in a) between groups | a) MGPQ (0-78)  b) N.R.  c) N.R.  d) Stabilometric assessment (not considered in this review)  e) post-treatment | a) SuPeR: the same protocol of Morone et al. (2012)  b) Back school program with: the same protocol of Morone et al. (2011) | | a)  EG: MGPQ: -44%‡24% †; +  CG: MGPQ: -39%‡15% †; +  b)  - MGPQ: 5%*; (‡); *p*=0.436  † authors reported the % improvement with respect to the maximum achievable improvement |
| Vetrano et al. (2013) | a) Single-blind, RCT  b) Italy  c) Clinical  d) Convenience sample (*n*=40).  e) EG: *n*=20  f) *n*=20  g) N.R.  e) *n*=0 | a) CLBP  b) age 25-70 y, disease duration >3 m, minimum 12 w of 'wash-out' period after any conservative therapy  c) radicular syndrome, spinal or abdominal surgery, pregnancy | EG:  a) 52.6 y (10.5)  b) 80%  c) NR  CG :  a) 52.2 y (16.2)  b) 70%  c) NR  (+) for difference in a) and b) between groups | CG:  a) VAS: median=7.5; IQR= 5.7-8  b) ODI: median=28: IQR= 16-30.5  CG:  a) VAS: median=5; IQR= 4-7  b) ODI: median=24: IQR= 14-30  no statistical SD for a) and b) in all two groups | a) 11-VAS (0-10 mm)  b) ODI (0-100)  c) N.R.  d) N.R.  e) post-treatment, at 4 and 12 weeks | a) SuPeR but with more deformable cones at midline level, decreasing tactile-pressure inputs at midline level and  without taking consciousness of the body midline  b) Standard SuPeR: the same protocol of Morone et al. (2011) but with 5’ less of treatment duration and with an increase of tactile-pressure inputs at midline level | | a)  EG:  - 11-VAS: -2* (‡) at T1, -2* **(**‡) at T2, **-3*** (‡) at T3 (***p<*0.001**)  - ODI: **-14*** (‡) at T1, **-16* (**‡) at T2, **-20*** (‡) at T3 (***p<*0.001**)  CG:  - 11-VAS: **-2.5*** (‡) at T1, **-3.5*** **(**‡) at T2, **-5.5*** (‡) at T3 (***p<*0.001**)  - ODI: **-16*** (‡) at T1, **-16*** (‡) at T2 and **-21*** (‡) at T3 (***p*<0.001**)  *All median differences at follow-up periods calculated respect to the T0 anchored values*  b)  - 11-VAS: 0.5* (‡) at T1 (*p*=0.179), 1.5* (‡) at T2 (*p*=0.398), 2.5* (‡) at T3 (*p*=0.868)  - ODI: 2* (‡) at T1 (*p*=0.299), 0* (‡) at T2 (*p*=0.169), 1 (‡) at T3 (*p*=0.922) |
| ***Qualitative Study*** | | | | | | | | |
| Lauche et al. (2012) | a) Qualitative study embedded in a RCT (1)  b) Germany  c) N.R.  d) convenience sample  e) *n=*6  f) N.A.  g) N.R. | a) CNP  b) N.R.  c) N.R. | EG:  b) 33%  CG:  b) 100%  c) range: 4m-45 y (pooled data of the whole sample) | EG:  a) N.R.  b) N.R.  c) N.R.  CG:  a) N.R.  b) N.R.  c) N.R. | a) Themes and sub-themes emerged from interviews in which patients were asked to talk about their body image drawings  b) Visual interpretation of the Body Image Drawing (modified version for trunk as described by Moseley, 2008)  e) 3 d after the treatment | a) a single traditional cupping treatment for half of the patients  b) waiting list | | Interviews: patients refer changes in body perception of the neck as a feeling of swollen or distorted in proportion. These overestimations persist even when patients were aware of their actual appearance. Subjects in EG refer a reduction in neck size (smaller) as a relief from pain.  Body Image Drawing: at the baseline the drawn body showed noticeable discrepancies respect to a “normal” body (missing lines and augmented dimension of shoulders and neck) in 4 out 6 subjects. Body image appears to be changed in EG after treatment (smaller dimension of body parts, and lines more symmetric and complete). Also the CG subjects improve in drawings themselves but they were no more complete, nor matched a “normal” silhouette. |
| **Experimental Study** | | | | | | | | |
| ***Manipulation of visual body appearance*** | | | | | | | | |
| Preston and Newport (2011) | a) Exploratory experimental study  b) United Kingdom  c) Experimental  d) convenience sample  e) *n*=20  f) N.A.  g) N.R. | a) Hand OA  b) pain in the hands and/or fingers for >12 months  c) N.R. | a) 70.5 y (6.5)  b) 80%  c) NR | a) NR  b) NR  c) NR | a) 21-NRS (0–20)  b) N.R.  c) N.R.  d) N.R.  e) post-intervention | a) MIRAGE system: visuo-tactile illusion involving manipulations (stretching or shrinking) of patient’s hand (affected and unaffected) while experimenter gently pulling or pushing on part of the hand | | a) 85% of patients reported reduction in pain for both experimental conditions, only manipulating visual appearance of the affected hand: MD= N.R. (‡); (***p*<0.001** in a sub-set of 10 patients having multiple painful sites  b)  - Stretching condition (*n*= N.R.): 21-NRS= **-3.09** (‡); (+)  - Shrinking condition (*n*= N.R.): 21-NRS= **-2.68** (‡); (+) |
| Diers et al. (2013) | a) Experimental study  b) Germany  c) Experimental  d) Convenience sample  e) EG: *n*=18  f) Healthy controls (*n*=18)  g) N.R. | a) Bilateral CUBP  b) N.R.  c) current major depression or any other axis I or II mental disorder (DSM-IV Structured Interview) | EG:  a) 53.9 y (9.2)  b) NR  c) NR  CG:  a) 54.2 y (9.2)  b) NR  c) NR  NSSD for a) between groups | EG:  a) 11-NRS: 3.1 (1.5)  - Pressure PI (11-NRS): 7 (0.8)  - Electric PI (11-NRS): 7.3 (1)  b) N.R.  c) N.R.  CG:  a) 11-NRS: 3.6 (1.2)  - Pressure PI (11-NRS): 7.1 (0.6)  - Electric PI (11-NRS): 6.7 (0.6)  (0.6)  b) N.R.  c) N.R.  Significant SD Electric PeT and PTo: higher in E. Group (*p*=0.043; *p*=0.026) | a)  - PU for pressure stimulation applied to the TrP1 of the Trapezius muscle: 11-NRS (0–10)  - PU and PI for electrical stimulation applied to the TrP1 of the Trapezius muscle: 11-NRS (0–10)  b) N.R.  c) N.R.  d) post-intervention | On-line video feedback of neutral (hand dorsum) and affected (neck) body parts in enlarged, downscaled and unaltered fashion. | | - no significant influence on Electric and Pressure PU (*p=*0.986) and PI (*p*=0.825) for back hand condition  - significant influence of visual feedback conditions on Electric and Pressure PU and PI: ***p*<0.001**)  a)  EG:  - Downscaled Back Condition: PI for Pressure stimulation: -0.4 (-1 to 0.2); *p*=0.161; PI for electric stimulation: -1.1 (-1.7 to -0.5); ***p*=0.001**  - Size Control Back Condition: PI for Pressure stimulation: -0.1 (-0.6 to 0.5); *p*=0.834; PI for electric stimulation: -1.1 (-1.7 to -0.5); ***p*=0.001**  - Enlarged Back Condition: PI for Pressure stimulation: 0 (-0.7 to 0.7); *p*=0.954; PI for electric stimulation: -1.1 (-1.8. to -0.5); ***p*<0.001**  CG:  - Downscaled Back Condition: PI for Pressure stimulation: - 0.9 (1.7 to -0.2); ***p=*0.02;** PI for electric stimulation: - 2.1 (-2.8 to -1.4); ***p*<0.001**  - Size Control Back Condition: PI for Pressure stimulation: -1 (-1.8 to -0.2); ***p*=0.016**; PI for electric stimulation: -2.1 (-2.9 to -1.2); ***p*<0.001**  - Enlarged Back Condition: PI for Pressure stimulation: -0.8 (-1.5 to -0.1); ***p*=0.036**; PI for electric stimulation: -2 (-3 to -1); ***p*<0.001** |
| Stanton et al. (2018) | a) Pilot-experimental study  b) Australia  c) Experimental  d) Calculated a priori  e) *n*= 12  f) N.A.  g) N.R. | Knee OA  a) radiographic evidence of osteoarthritic changes, no palpable warmth, one of the following criteria: 1) age: >50 y, 2) morning stiffness <30’, 3) crepitus of the knee and (in absence of radiographys4) bony tenderness of the tibiofemoral joint line, 5) bony enlargement of the knee  b) rheumatoid or inflammatory arthritis | a) 67.3 y (9.9)  b) 75%  c) 16.5 (14.3) | a) 101-NRS:  -maximum pain past 48h: 66.3 (28.6)  -minimum pain past 48h: 6.3 (10.9)  b) OKSQ: 24.1 (8.1)  c) Perceived knee size (% of the true size): 104 (0.05) | a) 101-NRS (0-100) immediately and 48h after Session 2, prior Session 3  b) OKSQ (0-48)  c)  - FreKAQ (0-36)  - Perceived Knee Size (% of the true size) as described by Gilpin et al. (2015)  d) N.R.  e) After each condition in experiment 1; (every 30 s in sustained condition); pre- and post- illusion in experiment 2 and 3 | Visual Illusion: MIRAGE system as described in Preston and Newport (2011), applied to the knee in 8 conditions: congruent (CO) and incongruent (IN) X vision only (VO), tactile only (TO) and visuotactile (VT) X stretch (ST) and shrink (SR): 30s with 2 min. rest (Session 1). Total duration: 1 h.  Session 2: the CO condition producing the greatest pain reduction was applied for 3 minute (sustained condition-SU) and repeated for 10 trials (repeated condition-RE), minimum 2 w apart.  Session 3: the RE condition of the Session 2 was repeated maximum 3 w apart. | | a)  VT illusion decreased pain by an average of 7.8 points (2.0 to 13.5), corresponding to a 25% reduction in pain both in CO (t_1,11_ =2.96, ***p*=0.013**) and in IN conditions. TO and VO conditions did not reduce pain (t_1,11_ =1.45, *p*=0.17); (t_1,11_ =-0:71, *p*=0.95)  - SU condition prolonged analgesia, but did not increase it: (Session 1: t_1,10_ =0.52, *p*=0.61; Session 3: t_1,7=-_0.697, *p*=0.51)  - RE condition (with congruent VT illusion) increased the analgesic effect with an average pain decrease of **20 points** (6.9 to 33.1), corresponding to a 40% pain reduction. In session 3 SU condition produce pain reduction: pre-illusion: post-illusion: t_1,6_ =3.9, *p*=0.008)  b)  - The CO-VT condition did not differ from the IN-VT condition, controlled for vision: no effect of Condition (F_1,11_ =0.032, *p*=0.86), Condition x Time interaction (F_1,11_ = 0.34, *p*=0.57), suggesting that analgesia was provided by both conditions when identical visual manipulations occurred; in contrast CO-VT reduce more pain than IN-VT when controlled for tactile input: Time effect (F_1,11_ =5.23, ***p*=0.043**), Condition x Time interaction (F_1,11_ = 5.29, ***p*=0.042**)  - CO-VT reduce pain more than TO and VO illusions: condition x Time interaction (F_2,22_ =4.2, ***p*=0.028**) |
| ***Manipulation of visual body appearance + Cognitive Manipulation*** | | | | | | | | |
| Nishigami et al. (2019) | a) Pilot-experimental study  b) Japan  c) Experimental  d) Convenience sample  e) *n*=2  f) N.A.  g) *n*=0 | CLBP  a) symptoms duration >3, back soreness during lifting | Subject A  a) 72 y  b) NR  c) 20 y  Subject B  a) 34 y  b) NR  c) 1 y | Subject A:  a) at rest: 80/100 §; during motion : 80/100 §  b) RMDQ: 16  c) Body Perception:  - FreBAQ: 29/36  Subject B:  a) at rest: 10/100 §; during motion : 20/100 §  b) RMDQ: 5  c) Body Perception:  - FreBAQ: 0/36  *§ converted on a 0-100 scale from the original 0-10 scale reported by the authors, multiplying each score for 10* | a) Pain: 101-NRS  (0-100) during a lifting task  b) N.R.  c) Embodiment: modified embodiment questionnaire (0-18)  d) Fear : 101-NRS (0–100); Perceived Strength (0–100); Perceived Confidence (0–100)  e) post-intervention | Visual Illusion: MIRAGE system as described in Preston and Newport (2011), applied to low back during a lifting task  a) participants watched a modified version of their back (muscled, fit-looking strong, back) during a lifting task  b)  - reshaped image of their back  - normal shaped condition | | Subject A:  - Embodiment (0-18): no difference for all three conditions: MD: 0  - Pain: 101-NRS= **-30** (Strong condition); 3 (Reshaped condition) vs. Normal condition  - Fear: 101-NRS= -12 (Strong condition); -3 (Reshaped Condition) vs. Normal Condition  Subject B:  - Embodiment (0-18): -13 (Strong condition) vs. Reshaped and Normal Condition (16)  - Pain: 101-NRS= 0 (Strong condition); +6 (Reshaped condition) vs. Normal condition  - Fear: 101-NRS= 2 (Strong condition); +2 (Reshaped Condition) vs. Normal Condition |

***Abbreviations*:** ACR: American College of Rheumatology; CLBP: Chronic Low Back Pain; LBP: Low Back Pain; TP: Thoracic Pain; CNP: Chronic Neck Pain; FM: Fibromyalgia; OA: Osteoarthritis; MPQ: VAS: Visual Analog Scale; 11-NRS: 11-point Numeric Rating Scale; MGPQ: McGill Pain Questionnaire; ODI: Oswestry Disability Index; RMDQ: Roland Morris Disability Questionnaire; TSK: Tampa Scale of Kinesiophobia; PSEQ: Pain Self-Efficacy Questionnaire; FIQ: Fibromyalgia Impact Questionnaire; RCT: randomized controlled trial; SuPeR: Surface for Perceptive Rehabilitation; TENS: Transcutaneous Electrical Nerve Stimulation; NSAIDs: Nonsteroidal Anti-Inflammatory Drugs; WDI: Waddlle Disability Index; SF-36: Short-Form 36; Chronic Pain Grade= CPG; OKSQ: Oxford Knee Score Questionnaire; FreKAQ= Fremantle Knee Awareness Questionnaire; DSM-IV: Diagnostic and Statistical Manual of Mental Disorders IV; TrP= Trigger Point; PU= Pain Unpleasantness; PI= Pain Intensity; PeT= Perception Threshold; PT= Pain Threshold; PTo= Pain Tolerance; RCT: Randomized Controlled Trial; COT: Cross-Over Trial; RCNIT: Randomized Controlled Non-Inferiority Trial; N.R.: not reported; SD: Standard Deviation; MD: Mean Difference; CI95%: Confidence Interval; *: median difference; +: *p-value* not reported; ‡: 95% CI not reported; SD: statistical difference; y: year/s; m: month/s; h: hour/s; EG: Experimental Group; CG: Control Group**, *p-value*** are reported in bold if statistically significant, other values in **bold** if clinically significant, through the Minimal Clinically Important Difference: for low-back pain, was considered as clinically significant 13 points on the ODI (3,4), 30% on VAS/NRS for pain (5,6), 2 to 3 points (or 8 to 12%) on the RMDQ for function (5,7) and 4.5 cm for the Active Lumbar Flexion (8). For neck pain, was considered 3.5 to 5 U on the 50-U Neck Pain Disability Index or 7 to 10% change (9,10) for function and 2.5 on an 10-U NRS (25% change) for pain (9).

**Refererences:**

1. Lauche R, Cramer H, Hohmann C, Choi K-E, Rampp T, Saha FJ, et al. The effect of traditional cupping on pain and mechanical thresholds in patients with chronic nonspecific neck pain: a randomised controlled pilot study. Evid Based Complement Alternat Med. 2012;2012:429718.
2. Wand BM, O’Connell NE, Di Pietro F, Bulsara M. Managing chronic nonspecific low back pain with a sensorimotor retraining approach: exploratory multiple-baseline study of 3 participants. Phys Ther. 2011 Apr;91(4):535–46.
3. Copay AG, Glassman SD, Subach BR, Berven S, Schuler TC, Carreon LY. Minimum clinically important difference in lumbar spine surgery patients: a choice of methods using the Oswestry Disability Index, Medical Outcomes Study questionnaire Short Form 36, and pain scales. Spine J. 2008 Dec;8(6):968–74.
4. Johnsen LG, Hellum C, Nygaard ØP, Storheim K, Brox JI, Rossvoll I, et al. Comparison of the SF6D, the EQ5D, and the oswestry disability index in patients with chronic low back pain and degenerative disc disease. BMC Musculoskeletal Disorders. 2013 Apr 26;14(1):148.
5. Ostelo RWJG, Deyo RA, Stratford P, Waddell G, Croft P, Von Korff M, et al. Interpreting change scores for pain and functional status in low back pain: towards international consensus regarding minimal important change. Spine. 2008 Jan 1;33(1):90–4.
6. Farrar JT, Young JP, LaMoreaux L, Werth JL, Poole RM. Clinical importance of changes in chronic pain intensity measured on an 11-point numerical pain rating scale. Pain. 2001 Nov;94(2):149–58.
7. Bombardier C, Hayden J, Beaton DE. Minimal clinically important difference. Low back pain: outcome measures. J Rheumatol. 2001 Feb;28(2):431–8.
8. Ekedahl H, Jonsson B, Frobell RB (2012) Fingertip-to-floor test and straight leg raising test: validity, responsiveness, and predictive value in patients with acute/sub acute low back pain. A*rchives of Physical Medicine and Rehabilitation* 93(12): 2210-2215. DOI: 10.1016/j.apmr.2012.04.020
9. Pool JJM, Ostelo RWJG, Hoving JL, Bouter LM, de Vet HCW. Minimal clinically important change of the Neck Disability Index and the Numerical Rating Scale for patients with neck pain. Spine. 2007 Dec 15;32(26):3047–51.
10. Stratford P, Riddle D, Binkley J, Spadoni G, Westaway M, Padfiled B. Using the Neck Disability Index to make decisions concerning individual patients. Physiother Can. 2009;Stratford PW, Riddle DL, Binkley JM, et al. Using the Neck Disability Index to make decisions concerning individual patients. Physiother Can 1999; Spring:107–19.:107–19.

# Additional File 8 - Supplementary Table S7: Excluded studies with reasons

| **Full-Text not found** | **Reasons for exclusion** |
| --- | --- |
| Skoyles JR. (1990). Is there a genetic component to body schema? Trends Neurosci. Oct; 13, (10), 409. doi: [10.1016/0166-2236(90)90120-y](https://doi.org/10.1016/0166-2236(90)90120-y) | Full text not found |
| Korin H, Tobis JS. (1971) Kinesthetic perceptual disturbance in musculoskeletal disease. J Chronic Dis. Jul; 24, (2), 209-213. doi: [10.1016/0021-9681(71)90111-1](https://doi.org/10.1016/0021-9681(71)90111-1) | Full text not found |
| Lisanti PA. (1989). Perceived body space and self-esteem in adult males with and without chronic low back pain. Orthop Nurs.  May-Jun; 8, (3), 49-56. | Full text not found |
| Lisanti P, Verdisco LA. (1994). Perceived body space and self-esteem in adult females with chronic low back pain. Orthop Nurs. Mar-Apr;13, (2), 55-63 | Full text not found |
| Patterson P. (1980). Awareness is key to rheumatoid arthritis. AORN Journal. 32, (4), 614-662 | Full text not found |
|  |  |
| **Studies Excluded for Language** | **Reasons for exclusion** |
| Bekrater-Bodmann R. (2015). Mirror therapy for inflammatory rheumatic pain: Potentials and limitations. Z Rheumatol. Nov,74, (9), 793-800. doi:10.1007/s00393-015-1629-7 | German language |
| Brucke H. (1963). On an unusual body image disorder after surgical correction of the position of the hip joint. Wien Klin Wochenschr. Jul, 5, 75, 540-541 | German language |
| Deparis N. (2009). Rheumatoid arthritis has badly affected my life. Soins. Sep, (738), 52. | French Language |
| Graindorge C. (2006). Back’s pain, lack of words: about body’s image. Archives de pédiatrie. 13, 761–771. | French language |
| Jabłońska-Brzozowska J. (2016). Between mind and body. Psychologicall methods for patients with musculoskeletal pain. Wiad Lek. 69, (4), 650-654. | Polish language |
| Levenig CG, [Hasenbring MI](https://www.ncbi.nlm.nih.gov/pubmed/?term=Hasenbring%20MI%5BAuthor%5D&cauthor=true&cauthor_uid=27333766), [Kleinert J](https://www.ncbi.nlm.nih.gov/pubmed/?term=Kleinert%20J%5BAuthor%5D&cauthor=true&cauthor_uid=27333766), [Kellmann M](https://www.ncbi.nlm.nih.gov/pubmed/?term=Kellmann%20M%5BAuthor%5D&cauthor=true&cauthor_uid=27333766). (2016). Body Image and Low Back Pain. Schmerz. 30, 437–443. doi:[10.1007/s00482-016-0122-9](https://doi.org/10.1007/s00482-016-0122-9) | German language |
| Weitbrecht WU, [Rice C](https://www.ncbi.nlm.nih.gov/pubmed/?term=Rice%20C%5BAuthor%5D&cauthor=true&cauthor_uid=14770349), [Schäfer W](https://www.ncbi.nlm.nih.gov/pubmed/?term=Sch%C3%A4fer%20W%5BAuthor%5D&cauthor=true&cauthor_uid=14770349). (2004). Phantom sensations and peripheral induced neglect following implantation of total hip prosthesis. Fortschr Neurol Psychiatr.  Feb, 72, (2), 93-97 | German language |

| **Studies Excluded for Insufficient Data** | **Reasons for exclusion** |
| --- | --- |
| Calsius J, et al. Body Awareness in fibromyalgic patients with alexithymia: a qualitative study. Abstract retrieved from the Psychotherapy and Psychosomatics Conference: 22nd World Congress on Psychosomatic Medicine, Volume: 82. | Conference poster: insufficient data provided. |
| Calsius J, Courtois I, Stiers J, De Bie J. (2015). How do fibromyalgia patients with alexithymia experience their body? A qualitative approach. SAGE Open. January-March, 1–10. | The study investigated the concept of embodiment and body awareness with a connotation relative to the salience of body stimuli illness status (theme one) and social interaction (theme three). In the emerged theme one (alienation of the lived body) partially emerges interesting findings about the embodiment (body experienced as "not mine") only in sub-themes b) - the alienated body. However, we think that this information alone is insufficient to be analysed and discussed for the purposes of this review. |
| Stevens-Lapsley J, Kittelson A, Systemic Pain Modulation Is Related to Body Perception in People with Knee Osteoarthritis. Abstract retrieved from Abstracts of ACR/ARHP Annual Meeting (2014). ABSTRACT NUMBER: 1282. | Data about overestimation in visual size estimation task were not reported for patients group: insufficient data. Authors contacted by e-mail without success. |

| **Studies Excluded for Outcome Measures Included** | **Reasons for exclusion** |
| --- | --- |
| Paolucci T, [Baldari C](https://www.ncbi.nlm.nih.gov/pubmed/?term=Baldari%20C%5BAuthor%5D&cauthor=true&cauthor_uid=26884794), [Di Franco M](https://www.ncbi.nlm.nih.gov/pubmed/?term=Di%20Franco%20M%5BAuthor%5D&cauthor=true&cauthor_uid=26884794), [Didona D](https://www.ncbi.nlm.nih.gov/pubmed/?term=Didona%20D%5BAuthor%5D&cauthor=true&cauthor_uid=26884794), [Reis V](https://www.ncbi.nlm.nih.gov/pubmed/?term=Reis%20V%5BAuthor%5D&cauthor=true&cauthor_uid=26884794), [Vetrano M](https://www.ncbi.nlm.nih.gov/pubmed/?term=Vetrano%20M%5BAuthor%5D&cauthor=true&cauthor_uid=26884794), [Iosa M](https://www.ncbi.nlm.nih.gov/pubmed/?term=Iosa%20M%5BAuthor%5D&cauthor=true&cauthor_uid=26884794), [Trifoglio D](https://www.ncbi.nlm.nih.gov/pubmed/?term=Trifoglio%20D%5BAuthor%5D&cauthor=true&cauthor_uid=26884794), [Zangrando F](https://www.ncbi.nlm.nih.gov/pubmed/?term=Zangrando%20F%5BAuthor%5D&cauthor=true&cauthor_uid=26884794), [Spadini E](https://www.ncbi.nlm.nih.gov/pubmed/?term=Spadini%20E%5BAuthor%5D&cauthor=true&cauthor_uid=26884794), [Saraceni VM](https://www.ncbi.nlm.nih.gov/pubmed/?term=Saraceni%20VM%5BAuthor%5D&cauthor=true&cauthor_uid=26884794), [Guidetti L](https://www.ncbi.nlm.nih.gov/pubmed/?term=Guidetti%20L%5BAuthor%5D&cauthor=true&cauthor_uid=26884794). (2016). A New Rehabilitation Tool in Fibromyalgia: The Effects of Perceptive Rehabilitation on Pain and Function in a Clinical Randomized Controlled Trial. Evid Based Complement Alternat Med. 2016:7574589. doi: 10.1155/2016/7574589 | The study included no outcome measures for pain |

| **Studies Excluded for Methodology Adopted** | **Reasons for exclusion** |
| --- | --- |
| Nishigami T, Okuno H, Nakano H, Omura Y, Osumi M, **Eisemann SM, Tsujishita M, Mibu A, Ushida** T. (2012.) Effects of a Hardness Discrimination Task in Failed Back Surgery Syndrome with Severe Low Back Pain and Disturbed Body Image: Case study. J Nov Physiother S1:008. | In this case study the patient was asked to perform an hardness discrimination task pushing on the left lower back of another person, and simultaneously imagining as if it was his own’s lower back. It constitutes thus more a task involving the mental imagination of a perceptual task rather than properly a perceptual task. |

| **Studies Excluded for Participants** | **Reasons for exclusion** |
| --- | --- |
| Paolucci et al. (2017). The importance of trunk perception during brace treatment in moderate juvenile idiopathic scoliosis: What is the impact on self-image? Journal of Back and Musculoskeletal Rehabilitation 30, 203–210. | The mean age of participants was below 16 years old: out of inclusion criteria. |
| Picelli A, Negrini S, Zenorini A, Iosa M, Paolucci S, Smania N. (2016). Do adolescents with idiopathic scoliosis have body schema disorders? A cross-sectional study. J. Back and Musculoskeletal Rehabil 29, 89–96. doi: 10.3233/BMR-150602 | The mean age of participants was below 16 years old: out of inclusion criteria. |

| **Studies Excluded for Design** | **Reasons for exclusion** |
| --- | --- |
| Bagust J, [Docherty S](https://www.ncbi.nlm.nih.gov/pubmed/?term=Docherty%20S%5BAuthor%5D&cauthor=true&cauthor_uid=26453504), [Abdul Razzak R](https://www.ncbi.nlm.nih.gov/pubmed/?term=Abdul%20Razzak%20R%5BAuthor%5D&cauthor=true&cauthor_uid=26453504). (2015). Re: “High variability of the subjective visual vertical test of vertical perception, in some people with neck pain e Should this be a standard measure of cervical proprioception?” Man Ther. Dec, 20, (6), e18. doi: 10.1016/j.math.2015.09.011 | Letter to editor: did not provide original or unpublished data. Reference list was manually scanned for potential additional sources |
| Boesch [E](https://www.ncbi.nlm.nih.gov/pubmed/?term=Boesch%20E%5BAuthor%5D&cauthor=true&cauthor_uid=26588692), [Bellan V](https://www.ncbi.nlm.nih.gov/pubmed/?term=Bellan%20V%5BAuthor%5D&cauthor=true&cauthor_uid=26588692), [Moseley GL](https://www.ncbi.nlm.nih.gov/pubmed/?term=Moseley%20GL%5BAuthor%5D&cauthor=true&cauthor_uid=26588692), [Stanton TR](https://www.ncbi.nlm.nih.gov/pubmed/?term=Stanton%20TR%5BAuthor%5D&cauthor=true&cauthor_uid=26588692). (2016). The effect of bodily illusions on clinical pain: a systematic review and meta-analysis. PAIN. 157, 516–529. doi: 10.1097/j.pain.0000000000000423 | Systematic review and meta-analysis: Reference list was manually scanned for potential additional sources. |
| Haggard P, Wolpert D.M. (2005). Disorders of Body Scheme. Higher-Order Motor Disorders, Ed. Freund, Jeannerod, Hallett & Leiguarda (Oxford University Press). | Book chapter. Reference list was manually scanned for potential additional sources |
| McCabe et al. Phantoms in rheumatology. Osteoarthritic Joint Pain: Novartis Foundation Symposium 260. Volume 260 | Book chapter. Reference list was manually scanned for potential additional sources |
| Moseley GL, Flor H. (2012). Targeting Cortical Representations in the Treatment of Chronic Pain: A Review. Neurorehabilitation and Neural Repair. 26, (6), 646–652. doi: 10.1177/1545968311433209 | This narrative review was focused on cortical representation plasticity: out of the scope of this review. Reference list was manually scanned for potential additional sources |
| Moseley GL, [Gallace A](https://www.ncbi.nlm.nih.gov/pubmed/?term=Gallace%20A%5BAuthor%5D&cauthor=true&cauthor_uid=21477616), [Spence C](https://www.ncbi.nlm.nih.gov/pubmed/?term=Spence%20C%5BAuthor%5D&cauthor=true&cauthor_uid=21477616). (2012). Bodily illusions in health and disease: Physiological and clinical perspectives and the concept of a cortical 'body matrix. Neuroscience and Biobehavioral Reviews. 36, 34–46. doi: 10.1016/j.neubiorev.2011.03.013 | Narrative review: reference list was manually scanned for potential additional sources |
| Pelletier [R](https://www.ncbi.nlm.nih.gov/pubmed/?term=Pelletier%20R%5BAuthor%5D&cauthor=true&cauthor_uid=25953594), [Higgins J](https://www.ncbi.nlm.nih.gov/pubmed/?term=Higgins%20J%5BAuthor%5D&cauthor=true&cauthor_uid=25953594), [Bourbonnais D](https://www.ncbi.nlm.nih.gov/pubmed/?term=Bourbonnais%20D%5BAuthor%5D&cauthor=true&cauthor_uid=25953594). (2015). Addressing Neuroplastic Changes in Distributed Areas of the Nervous System Associated With Chronic Musculoskeletal Disorders. Phys Ther. Nov, 95, (11), 1582-91. doi: 10.2522/ptj.20140575 | Perspective article: reference list was manually scanned for potential additional sources. |
| Puentedura EL, Flynn TW. (2016). Combining manual therapy with pain neuroscience education in the treatment of chronic low back pain: A narrative review of the literature. Physiotherapy Theory and Practice, June, 32, 5, 408-414. doi: 10.1080/09593985.2016.1194663 | Narrative review: reference list was manually scanned for potential additional sources |
| Schneider [M](https://www.ncbi.nlm.nih.gov/pubmed/?term=Schneider%20M%5BAuthor%5D&cauthor=true&cauthor_uid=19121462), [Vernon H](https://www.ncbi.nlm.nih.gov/pubmed/?term=Vernon%20H%5BAuthor%5D&cauthor=true&cauthor_uid=19121462), [Ko G](https://www.ncbi.nlm.nih.gov/pubmed/?term=Ko%20G%5BAuthor%5D&cauthor=true&cauthor_uid=19121462), [Lawson G](https://www.ncbi.nlm.nih.gov/pubmed/?term=Lawson%20G%5BAuthor%5D&cauthor=true&cauthor_uid=19121462), [Perera J](https://www.ncbi.nlm.nih.gov/pubmed/?term=Perera%20J%5BAuthor%5D&cauthor=true&cauthor_uid=19121462). (2009). Chiropractic management of fibromyalgia syndrome: a systematic review of the literature. J Manipulative Physiol Ther. Jan, 32, (1), 25-40. doi: 10.1016/j.jmpt.2008.08.012 | Systematic review: reference list was manually scanned for potential additional sources |
| Tsay [A](https://www.ncbi.nlm.nih.gov/pubmed/?term=Tsay%20A%5BAuthor%5D&cauthor=true&cauthor_uid=25783221), [Allen TJ](https://www.ncbi.nlm.nih.gov/pubmed/?term=Allen%20TJ%5BAuthor%5D&cauthor=true&cauthor_uid=25783221), [Proske U](https://www.ncbi.nlm.nih.gov/pubmed/?term=Proske%20U%5BAuthor%5D&cauthor=true&cauthor_uid=25783221), [Giummarra MJ](https://www.ncbi.nlm.nih.gov/pubmed/?term=Giummarra%20MJ%5BAuthor%5D&cauthor=true&cauthor_uid=25783221). (2015). Sensing the body in chronic pain: A review of psychophysical studies implicating altered body representation. Neuroscience and Biobehavioral Reviews*,* 52, 221–232. doi: 10.1016/j.neubiorev.2015.03.004 | Narrative review: reference list was manually scanned for potential additional sources |
| Valenzuela CM. (2012). Chronic pain and disturbances in body awareness. Revista Chilena de Neuropsicología, vol. 7, núm. 1, pp. 26-37. | Narrative review: reference list was manually scanned for potential additional sources |

| **Studies Excluded for Aim of the study** | **Reasons for exclusion** |
| --- | --- |
| Grilo A, Ribeiro M, Nogueira AM. (2012). Claustrophobia and adherence in magnetic resonance imaging procedure. Int.J. Behav. Med. 19, (Suppl 1): S1–S341*.* Abstracts retrieved from Abstracts of the ICBM 2012 Meeting. doi: 10.1007/s12529-012-9247-0 | In these abstracts of congress the topics investigated are out of the scope of this review. |
| Stern MJ, Guiles RA, Sigafus PB, Gevirtz R. (2013). Comparing Increases in HRV Between EMG Biofeedback and HRV Biofeedback. Appl Psychophysiol Biofeedback. 38, 213–238. Abstract retrieved from abstracts of Papers Presented at the 44th Annual Meeting of the Association for Applied Psychophysiology and Biofeedback. | In these abstracts of congress only Sobie et al. (“Body Schema and Anatomical Re-framing in the Feldenkrais Method for Low Back Pain”) has been further assessed for eligibility but was not possible to establish if intervention used fit with the concept on somatoperception used for the scope of this review: insufficient data. |
| Abstracts of IASP 16TH WORLD CONGRESS ON PAIN. | In these abstracts of congress, the topics investigated are out of the scope of this review. |
| Afrell M, [Biguet G](https://www.ncbi.nlm.nih.gov/pubmed/?term=Biguet%20G%5BAuthor%5D&cauthor=true&cauthor_uid=17727540), [Rudebeck CE](https://www.ncbi.nlm.nih.gov/pubmed/?term=Rudebeck%20CE%5BAuthor%5D&cauthor=true&cauthor_uid=17727540). (2007). Living with a body in pain – between acceptance and denial. Scand J Caring Sci. 21; 291–296. doi: [10.1111/j.1471-6712.2007.00475.x](https://doi.org/10.1111/j.1471-6712.2007.00475.x) | The study investigate the psychological topic of the body acceptance in an existential perspective as attitudes towards own’s life situation: in this sense it is addressed the theme of painful body integration into the self. |
| Anderson B, Strand LI, [Råheim](https://www.tandfonline.com/author/R%C3%A5heim%2C+M%C3%A5lfrid) M. (2007). The Effect of Long-Term Body Awareness Training Succeeding a Multimodal Cognitive Behavior Program for Patients with Widespread Pain. Journal of Musculoskeletal Pain. 15, (3), 19-29. doi: 10.1300/J094v15n03_04 | In this study, the Body Awareness Treatment was based on relaxation, coordination, flexibility, breathing, functional movement and strength: out of the scope of this review. |
| Azcarate E. (1969). Body boundary and psychological control in an arthritic population. J Proj Tech Pers Assess. Dec, 33, (6), 493-500. doi: [10.1080/0091651X.1969.10380178](https://doi.org/10.1080/0091651X.1969.10380178) | The study analysed the inner and outer control style of psychological states. The term 'body awareness' emerge as a general construct relating to self-knowledge, thus is not conceptually intended within the domain of implicit/explicit somatoperception considered for this review (reference framework of Longo et al. 2010; 2016). |
| Baranowsky J, [Klose P](https://www.ncbi.nlm.nih.gov/pubmed/?term=Klose%20P%5BAuthor%5D&cauthor=true&cauthor_uid=19672601), [Musial F](https://www.ncbi.nlm.nih.gov/pubmed/?term=Musial%20F%5BAuthor%5D&cauthor=true&cauthor_uid=19672601), [Häuser W](https://www.ncbi.nlm.nih.gov/pubmed/?term=H%C3%A4user%20W%5BAuthor%5D&cauthor=true&cauthor_uid=19672601), [Dobos G](https://www.ncbi.nlm.nih.gov/pubmed/?term=Dobos%20G%5BAuthor%5D&cauthor=true&cauthor_uid=19672601), [Langhorst J](https://www.ncbi.nlm.nih.gov/pubmed/?term=Langhorst%20J%5BAuthor%5D&cauthor=true&cauthor_uid=19672601). (2009). Qualitative systemic review of randomized controlled trials on complementary and alternative medicine treatments in fibromyalgia. Rheumatol Int. Nov, 30, (1), 23. doi: 10.1007/s00296-009-0977-5 | Systematic Review: RCT's included in this review evaluated Body Awareness treatment not conceptually intended within the domain of implicit/explicit somatoperception considered for this review (reference framework of Longo et al. 2010; 2016). Reference list was manually scanned for potential additional sources. |
| Bergström M, Ejelöv M, Stålnacke BM. (2014). One-year follow-up of body awareness and perceived health after participating in a multimodal pain rehabilitation programme – A pilot study. European Journal of Physiotherapy. Early Online: 1–9. Doi: 10.3109/21679169.2014.935802 | The study investigated Body Awarenss Treatment. The term 'body awareness' was intended as a general construct relating to self-knowledge, and awareness with respect to the illness, thus is not conceptually intended within the domain of implicit/explicit somatoperception considered for this review (reference framework of Longo et al. 2010; 2016). |
| Borg C, [Chouchou F](https://www.ncbi.nlm.nih.gov/pubmed/?term=Chouchou%20F%5BAuthor%5D&cauthor=true&cauthor_uid=29719416), [Dayot-Gorlero J](https://www.ncbi.nlm.nih.gov/pubmed/?term=Dayot-Gorlero%20J%5BAuthor%5D&cauthor=true&cauthor_uid=29719416), [Zimmerman P](https://www.ncbi.nlm.nih.gov/pubmed/?term=Zimmerman%20P%5BAuthor%5D&cauthor=true&cauthor_uid=29719416), [Maudoux D](https://www.ncbi.nlm.nih.gov/pubmed/?term=Maudoux%20D%5BAuthor%5D&cauthor=true&cauthor_uid=29719416), [Laurent B](https://www.ncbi.nlm.nih.gov/pubmed/?term=Laurent%20B%5BAuthor%5D&cauthor=true&cauthor_uid=29719416), [Michael GA](https://www.ncbi.nlm.nih.gov/pubmed/?term=Michael%20GA%5BAuthor%5D&cauthor=true&cauthor_uid=29719416). (2018). Pain and emotion as predictive factors of interoception in fibromyalgia. Journal of Pain Research. 11, 823–835. doi: 10.2147/JPR.S152012 | Study investigating interoception: out of the scope of this review. |
| Borg C, Emond FC, Colson D, Laurent B, Michael GA. (2015). Attentional focus on subjective interoceptive experience in patients with fibromyalgia. Brain and Cognition. 101, 35–43. doi: 10.1016/j.bandc.2015.10.002 | Study investigating the generalized hypervigilance hypothesis of fibromyalgia by assessing non-aversive perceptions like those arising spontaneously on the hands (i.e., Spontaneous sensations): out of the scope of this review. |
| Bowering KJ, [Butler DS](https://www.ncbi.nlm.nih.gov/pubmed/?term=Butler%20DS%5BAuthor%5D&cauthor=true&cauthor_uid=24535054), [Fulton IJ](https://www.ncbi.nlm.nih.gov/pubmed/?term=Fulton%20IJ%5BAuthor%5D&cauthor=true&cauthor_uid=24535054), [Moseley GL](https://www.ncbi.nlm.nih.gov/pubmed/?term=Moseley%20GL%5BAuthor%5D&cauthor=true&cauthor_uid=24535054). (2014). Motor Imagery in People With a History of Back Pain, Current Back Pain, Both, or Neither. Clin J Pain. 30, 1070–1075. doi: 10.1097/AJP.0000000000000066 | Study on laterality recognition: out of the scope of this review. |
| Bravo C. (2016). Effectiveness of Basic Body Awareness Therapy (BBAT) in Patients Suffering From Fibromyalgia (BBAT). ClinicalTrials.Gov. Identifier: NCT02830295. | Study protocol, investigating the Body Awareness Therapy involved generic control of movement, awareness of posture and quality of movement, and breathing: out of the scope of this review. |
| Bravo C, [Skjaerven LH](https://www.ncbi.nlm.nih.gov/pubmed/?term=Skjaerven%20LH%5BAuthor%5D&cauthor=true&cauthor_uid=29723080), [Espart A](https://www.ncbi.nlm.nih.gov/pubmed/?term=Espart%20A%5BAuthor%5D&cauthor=true&cauthor_uid=29723080), [Guitard Sein-Echaluce L](https://www.ncbi.nlm.nih.gov/pubmed/?term=Guitard%20Sein-Echaluce%20L%5BAuthor%5D&cauthor=true&cauthor_uid=29723080), [Catalan-Matamoros D](https://www.ncbi.nlm.nih.gov/pubmed/?term=Catalan-Matamoros%20D%5BAuthor%5D&cauthor=true&cauthor_uid=29723080). (2018). Basic Body Awareness Therapy in patients suffering from fibromyalgia: A randomized clinical trial. Physiother Theory Pract. May, 3, 1-11. doi: 10.1080/09593985.2018.1467520 | Study investigating the Body Awareness Therapy involve d generic control of movement, awareness of posture, quality of movement, and breathing: out of the scope of this review. |
| Bray H, Moseley JL. (2011). Disrupted working body schema of the trunk in people with back pain. Br J Sports Med. 45, 168–173. doi: 10.1136/bjsm.2009.061978 | Study on laterality recognition: out of the scope of this review. |
| Brumagne S. (2013). What is the relation between proprioception and low back pain? Spinal Control: The Rehabilitation of Back Pain: State of the art and science. July, Pages 219-230. | Study on proprioception: out of the scope of this review. |
| Brummet [CM](https://www.ncbi.nlm.nih.gov/pubmed/?term=Brummett%20CM%5BAuthor%5D&cauthor=true&cauthor_uid=26835782), [Bakshi RR](https://www.ncbi.nlm.nih.gov/pubmed/?term=Bakshi%20RR%5BAuthor%5D&cauthor=true&cauthor_uid=26835782), [Goesling J](https://www.ncbi.nlm.nih.gov/pubmed/?term=Goesling%20J%5BAuthor%5D&cauthor=true&cauthor_uid=26835782), [Leung D](https://www.ncbi.nlm.nih.gov/pubmed/?term=Leung%20D%5BAuthor%5D&cauthor=true&cauthor_uid=26835782), [Moser SE](https://www.ncbi.nlm.nih.gov/pubmed/?term=Moser%20SE%5BAuthor%5D&cauthor=true&cauthor_uid=26835782), [Zollars JW](https://www.ncbi.nlm.nih.gov/pubmed/?term=Zollars%20JW%5BAuthor%5D&cauthor=true&cauthor_uid=26835782), [Williams DA](https://www.ncbi.nlm.nih.gov/pubmed/?term=Williams%20DA%5BAuthor%5D&cauthor=true&cauthor_uid=26835782), [Clauw DJ](https://www.ncbi.nlm.nih.gov/pubmed/?term=Clauw%20DJ%5BAuthor%5D&cauthor=true&cauthor_uid=26835782), [Hassett AL](https://www.ncbi.nlm.nih.gov/pubmed/?term=Hassett%20AL%5BAuthor%5D&cauthor=true&cauthor_uid=26835782). (2016). Preliminary Validation of the Michigan Body Map (MBM). Pain. June; 157, (6), 1205–1212. doi: 10.1097/j.pain.0000000000000506 | Validation study of a body chart: out of the scope of this review. |
| Case LK, Ceko M, Gracely JL, Richards EA, Olausson H, Catherine Bushnell M. (2016). Touch Perception Altered by Chronic Pain and by Opioid Blockade. eNeuro. Mar 10, 3, (1). pii: ENEURO.0138-15.2016. doi: 10.1523/ENEURO.0138-15.2016. | Study investigating the pleasantness of touch: out of the scope of this review. |
| Cornwell CJ, Schmitt MH. (1990). Perceived Health Status, Self-Esteem and Body Image in Women with Rheumatoid Arthritis or Systemic Lupus Erythematosus. Res Nurs Health. Apr, 13, (2), 99-107. | Studies investigating the body image under the construct of the satisfaction about own bodily appearance. |
| Cramer H, [Lauche R](https://www.ncbi.nlm.nih.gov/pubmed/?term=Lauche%20R%5BAuthor%5D&cauthor=true&cauthor_uid=23336342), [Haller H](https://www.ncbi.nlm.nih.gov/pubmed/?term=Haller%20H%5BAuthor%5D&cauthor=true&cauthor_uid=23336342), [Langhorst J](https://www.ncbi.nlm.nih.gov/pubmed/?term=Langhorst%20J%5BAuthor%5D&cauthor=true&cauthor_uid=23336342), [Dobos G](https://www.ncbi.nlm.nih.gov/pubmed/?term=Dobos%20G%5BAuthor%5D&cauthor=true&cauthor_uid=23336342), [Berger B](https://www.ncbi.nlm.nih.gov/pubmed/?term=Berger%20B%5BAuthor%5D&cauthor=true&cauthor_uid=23336342). (2013). I'm more in balance: a qualitative study of yoga for patients with chronic neck pain. Journal of Complementary and Alternative Medicine. 19, (6), 536-542. doi: 10.1089/acm.2011.0885 | Insufficient data: body image drawing and interview's record provided only by one subject despite the research design is not a case study. Moreover, within the topic of the 'Body Awareness" predominated the analysis of social, behavioral and emotional aspects. |
| Crowe [M](https://www.ncbi.nlm.nih.gov/pubmed/?term=Crowe%20M%5BAuthor%5D&cauthor=true&cauthor_uid=19854442), [Whitehead L](https://www.ncbi.nlm.nih.gov/pubmed/?term=Whitehead%20L%5BAuthor%5D&cauthor=true&cauthor_uid=19854442), [Gagan MJ](https://www.ncbi.nlm.nih.gov/pubmed/?term=Gagan%20MJ%5BAuthor%5D&cauthor=true&cauthor_uid=19854442), [Baxter GD](https://www.ncbi.nlm.nih.gov/pubmed/?term=Baxter%20GD%5BAuthor%5D&cauthor=true&cauthor_uid=19854442), [Pankhurst A](https://www.ncbi.nlm.nih.gov/pubmed/?term=Pankhurst%20A%5BAuthor%5D&cauthor=true&cauthor_uid=19854442), [Valledor V](https://www.ncbi.nlm.nih.gov/pubmed/?term=Valledor%20V%5BAuthor%5D&cauthor=true&cauthor_uid=19854442). (2010). Listening to the body and talking to myself – the impact of chronic lower back pain: A qualitative study. International Journal of Nursing Studies. 47, 586–592. doi: 10.1016/j.ijnurstu.2009.09.012. | The study investigated the impact of the disease (chronic low back pain) on peoples’ self-identity and self-image: out of the scope of this review. |
| Curtois I,  [Cools F](https://www.ncbi.nlm.nih.gov/pubmed/?term=Cools%20F%5BAuthor%5D&cauthor=true&cauthor_uid=25603742), [Calsius J](https://www.ncbi.nlm.nih.gov/pubmed/?term=Calsius%20J%5BAuthor%5D&cauthor=true&cauthor_uid=25603742). (2015). Effectiveness of body awareness interventions in fibromyalgia and chronic fatigue syndrome: A systematic review and meta-analysis. Journal of Bodywork & Movement Therapies. 19, 35-56. doi: 10.1016/j.jbmt.2014.04.003 | Systematic review assessing Body Awareness Therapy: out of the scope of this review. Reference list was manually scanned for potential additional sources. |
| Daenen [L](https://www.ncbi.nlm.nih.gov/pubmed/?term=Daenen%20L%5BAuthor%5D&cauthor=true&cauthor_uid=24118839), [Nijs J](https://www.ncbi.nlm.nih.gov/pubmed/?term=Nijs%20J%5BAuthor%5D&cauthor=true&cauthor_uid=24118839), [Cras P](https://www.ncbi.nlm.nih.gov/pubmed/?term=Cras%20P%5BAuthor%5D&cauthor=true&cauthor_uid=24118839), [Wouters K](https://www.ncbi.nlm.nih.gov/pubmed/?term=Wouters%20K%5BAuthor%5D&cauthor=true&cauthor_uid=24118839), [Roussel N](https://www.ncbi.nlm.nih.gov/pubmed/?term=Roussel%20N%5BAuthor%5D&cauthor=true&cauthor_uid=24118839). (2014). Changes in Pain Modulation Occur Soon After Whiplash Trauma but are not Related to Altered Perception of Distorted Visual Feedback. Pain Practice. Volume 14, Issue 7, 588–598.  doi: 10.1111/papr.12113 | Study on sensorimotor incongruence: out of the scope of this review. |
| Daenen [L](https://www.ncbi.nlm.nih.gov/pubmed/?term=Daenen%20L%5BAuthor%5D&cauthor=true&cauthor_uid=22525161), [Nijs J](https://www.ncbi.nlm.nih.gov/pubmed/?term=Nijs%20J%5BAuthor%5D&cauthor=true&cauthor_uid=22525161), [Roussel N](https://www.ncbi.nlm.nih.gov/pubmed/?term=Roussel%20N%5BAuthor%5D&cauthor=true&cauthor_uid=22525161), [Wouters K](https://www.ncbi.nlm.nih.gov/pubmed/?term=Wouters%20K%5BAuthor%5D&cauthor=true&cauthor_uid=22525161), [Van Loo M](https://www.ncbi.nlm.nih.gov/pubmed/?term=Van%20Loo%20M%5BAuthor%5D&cauthor=true&cauthor_uid=22525161), [Cras P](https://www.ncbi.nlm.nih.gov/pubmed/?term=Cras%20P%5BAuthor%5D&cauthor=true&cauthor_uid=22525161). (2012). Sensorimotor incongruence exacerbates symptoms in patients with chronic whiplash associated disorders: an experimental study. Rheumatology. 51, 1492 1499. doi: 10.1093/rheumatology/kes050 | Study on sensorimotor incongruence: out of the scope of this review. |
| - de Lussanet MHE, Behrendt F, Puta C, Schulte TL, Lappe M, Weiss T, Wagner H. (2013). Impaired visual perception of hurtful actions in patients with chronic low back pain. Human Movement Science. 32, 938–953. doi: 10.1016/j.humov.2013.05.002 | In the study it has been evaluated the perception of weight lifted by an actor: outcome measures does not involve perception abilities described in the conceptual model of Longo et al. (2010; 2016) |
| Duschek [S](https://www.ncbi.nlm.nih.gov/pubmed/?term=Duschek%20S%5BAuthor%5D&cauthor=true&cauthor_uid=26431269), [Montoro CI](https://www.ncbi.nlm.nih.gov/pubmed/?term=Montoro%20CI%5BAuthor%5D&cauthor=true&cauthor_uid=26431269), [Reyes Del Paso GA](https://www.ncbi.nlm.nih.gov/pubmed/?term=Reyes%20Del%20Paso%20GA%5BAuthor%5D&cauthor=true&cauthor_uid=26431269). (2017). Diminished Interoceptive Awareness in Fibromyalgia Syndrome. Behav Med. Apr-Jun, 43, (2), 100-107. doi: 10.1080/08964289.2015.1094442 | The study investigate the interoceptive awareness intended as the sensitivity to signal arising within the body. |
| Fogel A. (2013). Better or Worse a Study of Day-to-Day Changes over Five Months of Rosen Method Bodywork Treatment. Int J Ther Massage Bodywork. 6, (3), 14–24. | The study investigated the embodied self-awareness , intend as an increased awareness of internal emotional states: out of the scope of this review. |
| Gard G. (2005). Body awareness therapy for patients with fibromyalgia and chronic pain. Disabil Rehabil. Jun, 17, 27, (12), 725-8. doi:[10.1080/09638280400009071](https://doi.org/10.1080/09638280400009071) | The study investigating the Body Awareness Therapy involve d movements, breathing, massage and the unity of body and mind with a focus on emotions: out of the scope of this review. |
| Haller H, [Lauche R](https://www.ncbi.nlm.nih.gov/pubmed/?term=Lauche%20R%5BAuthor%5D&cauthor=true&cauthor_uid=26340656), [Cramer H](https://www.ncbi.nlm.nih.gov/pubmed/?term=Cramer%20H%5BAuthor%5D&cauthor=true&cauthor_uid=26340656), [Rampp T](https://www.ncbi.nlm.nih.gov/pubmed/?term=Rampp%20T%5BAuthor%5D&cauthor=true&cauthor_uid=26340656), [Saha FJ](https://www.ncbi.nlm.nih.gov/pubmed/?term=Saha%20FJ%5BAuthor%5D&cauthor=true&cauthor_uid=26340656), [Ostermann T](https://www.ncbi.nlm.nih.gov/pubmed/?term=Ostermann%20T%5BAuthor%5D&cauthor=true&cauthor_uid=26340656), [Dobos G](https://www.ncbi.nlm.nih.gov/pubmed/?term=Dobos%20G%5BAuthor%5D&cauthor=true&cauthor_uid=26340656). (2014). Craniosacral Therapy for the Treatment of Chronic Neck Pain: A Follow-up Study. The Journal of Alternative and Complementary Medicine. Vol. 20, No. 5. Oral Abstract Session 11: Clinical Manual Therapies. | Insufficient data: the study investigated Body Awareness as outcome measure but this measure has not been clearly defined in this proceeding. |
| Haller H, [Lauche R](https://www.ncbi.nlm.nih.gov/pubmed/?term=Lauche%20R%5BAuthor%5D&cauthor=true&cauthor_uid=26340656), [Cramer H](https://www.ncbi.nlm.nih.gov/pubmed/?term=Cramer%20H%5BAuthor%5D&cauthor=true&cauthor_uid=26340656), [Rampp T](https://www.ncbi.nlm.nih.gov/pubmed/?term=Rampp%20T%5BAuthor%5D&cauthor=true&cauthor_uid=26340656), [Saha FJ](https://www.ncbi.nlm.nih.gov/pubmed/?term=Saha%20FJ%5BAuthor%5D&cauthor=true&cauthor_uid=26340656), [Ostermann T](https://www.ncbi.nlm.nih.gov/pubmed/?term=Ostermann%20T%5BAuthor%5D&cauthor=true&cauthor_uid=26340656), [Dobos G](https://www.ncbi.nlm.nih.gov/pubmed/?term=Dobos%20G%5BAuthor%5D&cauthor=true&cauthor_uid=26340656). (2016). Craniosacral Therapy for the Treatment of Chronic Neck Pain. Clin J Pain. 32, 441–449. doi: 10.1097/AJP.0000000000000290 | The evaluation scale used for body awareness as a secondary outcome was referred to the "mind-body" connection: out of the scope of this review. |
| Hassan [BS](https://www.ncbi.nlm.nih.gov/pubmed/?term=Hassan%20BS%5BAuthor%5D&cauthor=true&cauthor_uid=11779753), [Mockett S](https://www.ncbi.nlm.nih.gov/pubmed/?term=Mockett%20S%5BAuthor%5D&cauthor=true&cauthor_uid=11779753), [Doherty M](https://www.ncbi.nlm.nih.gov/pubmed/?term=Doherty%20M%5BAuthor%5D&cauthor=true&cauthor_uid=11779753). (2002). Influence of elastic bandage on knee pain, proprioception, and postural sway in subjects with knee osteoarthritis. Ann Rheum Dis. 61, 24–28. doi:[10.1136/ard.61.1.24](https://doi.org/10.1136/ard.61.1.24) | The study investigated proprioception and balance: out of the scope of this review. |
| Hellström O, Bullington J, Karlsson G, Lindqvist P, Mattsson B. (1999). A phenomenological study of fibromyalgia. Patient perspectives. Scand J Prim Health Care. Mar, 17, (1), 11-6. doi:10.1080/028134399750002827 | The study investigated self-images of ill persons in relation to the meaning of the illness condition: out of the scope of the review. |
| Hildebrandt J. (2014). Prediction of Psychosocial Factors by Pain Drawing in Patients with Chronic Back Pain. Pain Medicine. 15, 1067–1069. | This editorial talked about pain drawing and the prediction of psychosocial factors: out of the scope of the review. |
| Hildebrandt M, [Fankhauser](https://www.ncbi.nlm.nih.gov/pubmed/?term=Fankhauser%20G%5BAuthor%5D&cauthor=true&cauthor_uid=28068962) G, [Meichtry](https://www.ncbi.nlm.nih.gov/pubmed/?term=Meichtry%20A%5BAuthor%5D&cauthor=true&cauthor_uid=28068962) A, [Luomajoki](https://www.ncbi.nlm.nih.gov/pubmed/?term=Luomajoki%20H%5BAuthor%5D&cauthor=true&cauthor_uid=28068962) H. (2017). Correlation between lumbar dysfunction and fat infiltration in lumbar multifidus muscles in patients with low back pain. BMC Musculoskeletal Disorders 18:12. doi: [10.1186/s12891-016-1376-1](https://dx.doi.org/10.1186%2Fs12891-016-1376-1) | In this study for body awareness was intended the tactile acuity (two-point discrimination threshold): out the scope of this review. |
| Horwitz EB, Theorell T, Anderberg UM. (2004). New technique for assessment of self-perception in fibromyalgia patients: A pilot study with video-interpretation. The Arts in Psychotherapy. 31, 153–164. doi: 10.1016/j.aip.2004.03.004 | Aim: This study analysed the perception of own's movements in fibromyalgic patients through the use of video interpretation. The perception of voluntarily movements is a criterion for exclusion. |
| Horwitz EB, Kowalskj J, Anderberg UM. (2006). Dance/movement therapy in fibromyalgia patients: Changes in self-figure drawings and their relation to verbal self-rating scales. The Arts in Psychotherapy. 33, 11–25. doi: 10.1016/j.aip.2005.05.004 | Aim: This study analysed the perception of own's movements in fibromyalgic patients through the use of video interpretation. The perception of voluntarily movements is a criterion for exclusion.  Insufficient data: for the self-image drawings the instructions ("as you see it") were generic and it is difficult to interpret the meaning under the patient's perspective: it is not clear if is required to draw how patients perceived their body or , more under a psychological point of view, their body image concerning body esteem, body satisfaction and self-acceptance |
| Horwitz EB, Theorell T, Anderberg UM. (2003). Fibromyalgia patients’ own experiences of video self-interpretation: A phenomenological-hermeneutic study. Scand J Caring Sci. 17, 257–264. | Aim: This study analysed the perception of own's movements in fibromyalgic patients through the use of video interpretation. The perception of voluntarily movements is a criteria for exclusion |
| Hotz-Boendermaker [S](https://www.ncbi.nlm.nih.gov/pubmed/?term=Hotz-Boendermaker%20S%5BAuthor%5D&cauthor=true&cauthor_uid=27244113), [Marcar VL](https://www.ncbi.nlm.nih.gov/pubmed/?term=Marcar%20VL%5BAuthor%5D&cauthor=true&cauthor_uid=27244113), [Meier ML](https://www.ncbi.nlm.nih.gov/pubmed/?term=Meier%20ML%5BAuthor%5D&cauthor=true&cauthor_uid=27244113), [Boendermaker B](https://www.ncbi.nlm.nih.gov/pubmed/?term=Boendermaker%20B%5BAuthor%5D&cauthor=true&cauthor_uid=27244113), [Humphreys BK](https://www.ncbi.nlm.nih.gov/pubmed/?term=Humphreys%20BK%5BAuthor%5D&cauthor=true&cauthor_uid=27244113). (2016). Reorganization in Secondary Somatosensory Cortex in Chronic Low Back Pain Patients. Spine (Phila Pa 1976).  Jun, 41, (11), E667-73. doi: 10.1097/BRS.0000000000001348 | This study investigated the reorganization of the sensory cortex by using functional magnetic resonance imaging (fMRI): out the scope of this review |
| Ishii Y, Tojo T, Terajima K, Terashima SG, Bechtold J. (1999). Intracapsular components do not change hip proprioception. J Bone Joint Surg [Br]. 81-B:345-8. doi: 10.1302/0301-620X.81B2.0810345 | The study investigated proprioception by evaluating the joint repositioning accuracy: out the scope of this review. |
| Kendall [SA](https://www.ncbi.nlm.nih.gov/pubmed/?term=Kendall%20SA%5BAuthor%5D&cauthor=true&cauthor_uid=14635300), [Brolin-Magnusson K](https://www.ncbi.nlm.nih.gov/pubmed/?term=Brolin-Magnusson%20K%5BAuthor%5D&cauthor=true&cauthor_uid=14635300), [Sören B](https://www.ncbi.nlm.nih.gov/pubmed/?term=S%C3%B6ren%20B%5BAuthor%5D&cauthor=true&cauthor_uid=14635300), [Gerdle B](https://www.ncbi.nlm.nih.gov/pubmed/?term=Gerdle%20B%5BAuthor%5D&cauthor=true&cauthor_uid=14635300), [Henriksson KG](https://www.ncbi.nlm.nih.gov/pubmed/?term=Henriksson%20KG%5BAuthor%5D&cauthor=true&cauthor_uid=14635300). (2000). A Pilot Study of Body Awareness Programs in the Treatment of Fibromyalgia Syndrome. Arthritis Care Res. Oct, 13, (5), 304-11. | Insufficient data: the concept of body awareness is vague; it is used as a generic term for the use of the body. It includes the body consciousness, body management, and deepened body experience. |
| Lauche [R](https://www.ncbi.nlm.nih.gov/pubmed/?term=Lauche%20R%5BAuthor%5D&cauthor=true&cauthor_uid=28146026), [Wayne PM](https://www.ncbi.nlm.nih.gov/pubmed/?term=Wayne%20PM%5BAuthor%5D&cauthor=true&cauthor_uid=28146026), [Fehr J](https://www.ncbi.nlm.nih.gov/pubmed/?term=Fehr%20J%5BAuthor%5D&cauthor=true&cauthor_uid=28146026), [Stumpe C](https://www.ncbi.nlm.nih.gov/pubmed/?term=Stumpe%20C%5BAuthor%5D&cauthor=true&cauthor_uid=28146026), [Dobos G](https://www.ncbi.nlm.nih.gov/pubmed/?term=Dobos%20G%5BAuthor%5D&cauthor=true&cauthor_uid=28146026), [Cramer H](https://www.ncbi.nlm.nih.gov/pubmed/?term=Cramer%20H%5BAuthor%5D&cauthor=true&cauthor_uid=28146026). (2017). Does Postural Awareness Contribute to Exercise-Induced Improvements in Neck Pain Intensity? A Secondary Analysis of a Randomized Controlled Trial Evaluating Tai Chi and Neck Exercises. SPINE*.* Volume 42, Number 16, pp. 1195–1200. doi: 10.1097/BRS.0000000000002078. | In this paper the scale measuring the construct of the Postural Awareness (The Postural Awareness Scale) represent a self-administered assessment of the global posture and does not contain any of the perceptual ability identified by Longo et al. (2010; 2016) in their theoretical model. |
| Leffler [AS](https://www.ncbi.nlm.nih.gov/pubmed/?term=Leffler%20AS%5BAuthor%5D&cauthor=true&cauthor_uid=10833556), [Kosek E](https://www.ncbi.nlm.nih.gov/pubmed/?term=Kosek%20E%5BAuthor%5D&cauthor=true&cauthor_uid=10833556), [Hansson P](https://www.ncbi.nlm.nih.gov/pubmed/?term=Hansson%20P%5BAuthor%5D&cauthor=true&cauthor_uid=10833556). (2000). The influence of pain intensity on somatosensory perception in patients suffering from subacute/chronic lateral epicondylalgia. European Journal of Pain. 4, 57–71. doi:[10.1053/eujp.1999.0159](https://doi.org/10.1053/eujp.1999.0159) | The study assess the sensory profile of patients by using the Quantitative Sensory Testing: out the scope of this review. |
| Loof H. et al. (2013). Psychometric Testing of the Body Awareness Questionnaire in a Swedish Sample of Patients Diagnosed with Rheumatoid Arthritis. Annals of the Rheumatic Diseases*.* 71**,** 734. | This poster of congress is a psychometric validation study of the scale Body Awareness Questionnaire in Swedish language: this scale was validated by Shields S. A., Mallroy M. E., & Simon. (The body awareness questionnaire: reliability and validity. Journal of Personality Assessment. 1989; 53: 802- 815) but the full text is not available. |
| Loof [H](https://www.ncbi.nlm.nih.gov/pubmed/?term=L%C3%B6%C3%B6f%20H%5BAuthor%5D&cauthor=true&cauthor_uid=22994423), [Johansson UB](https://www.ncbi.nlm.nih.gov/pubmed/?term=Johansson%20UB%5BAuthor%5D&cauthor=true&cauthor_uid=22994423), [Henriksson EW](https://www.ncbi.nlm.nih.gov/pubmed/?term=Henriksson%20EW%5BAuthor%5D&cauthor=true&cauthor_uid=22994423), [Lindblad S](https://www.ncbi.nlm.nih.gov/pubmed/?term=Lindblad%20S%5BAuthor%5D&cauthor=true&cauthor_uid=22994423), [Saboonchi F](https://www.ncbi.nlm.nih.gov/pubmed/?term=Saboonchi%20F%5BAuthor%5D&cauthor=true&cauthor_uid=22994423). (2013). Development and psychometric testing of the Swedish version of the Body Awareness Questionnaire. J Adv Nurs. Jul, 69, (7), 1643-51. doi: 10.1111/jan.12020 | The Body Awareness Questionnaire (BAQ) is a tool used to measure self-reported attentiveness to normal body processes. The BAQ measures the bodily focus of attention (e.g. subtle body cues and perceived body sensations). The ability to direct self-attentiveness to own's body signals may be an important prerequisite for perceptual processes described by Longo et al. (2010; 2016) in their framework model but it is not directly correlated with them. |
| Loof H, [Johansson](https://www.ncbi.nlm.nih.gov/pubmed/?term=Johansson%20UB%5BAuthor%5D&cauthor=true&cauthor_uid=25363521) UB, [Henriksson](https://www.ncbi.nlm.nih.gov/pubmed/?term=Henriksson%20EW%5BAuthor%5D&cauthor=true&cauthor_uid=25363521) EW, [Lindblad](https://www.ncbi.nlm.nih.gov/pubmed/?term=Lindblad%20S%5BAuthor%5D&cauthor=true&cauthor_uid=25363521) S, [Bullington](https://www.ncbi.nlm.nih.gov/pubmed/?term=Bullington%20J%5BAuthor%5D&cauthor=true&cauthor_uid=25363521) J. (2014). Body awareness in persons diagnosed with rheumatoid arthritis. Int J Qualitative Stud Health Well-being. 9, 24670. doi: [10.3402/qhw.v9.24670](https://dx.doi.org/10.3402%2Fqhw.v9.24670) | The study investigate the concept of the Body Awareness as the ability to notice bodily inner sensations and stimuli: The ability to direct self-attentiveness to own's body signals may be an important prerequisite for perceptual processes described by Longo et al. (2010; 2016) in their framework model but it is not directly correlated with them. |
| Loof [H](https://www.ncbi.nlm.nih.gov/pubmed/?term=L%C3%B6%C3%B6f%20H%5BAuthor%5D&cauthor=true&cauthor_uid=22994423), [Johansson UB](https://www.ncbi.nlm.nih.gov/pubmed/?term=Johansson%20UB%5BAuthor%5D&cauthor=true&cauthor_uid=22994423), Welin E, [Lindblad S](https://www.ncbi.nlm.nih.gov/pubmed/?term=Lindblad%20S%5BAuthor%5D&cauthor=true&cauthor_uid=22994423), [Saboonchi F](https://www.ncbi.nlm.nih.gov/pubmed/?term=Saboonchi%20F%5BAuthor%5D&cauthor=true&cauthor_uid=22994423). (2013). Pain and fatigue in adult patients with rheumatoid arthritis: Association with body awareness, demographic, disease-related, emotional and psychosocial factors*.* Open Journal of Nursing. 3, (02), 293-300. doi: 10.4236/ojn.2013.32040 | The construct of the Body Awareness was evaluated with the Body Awareness Questionnaire (BAQ) measuring the bodily focus of attention (e.g. subtle body cues and perceived body sensations). The ability to direct self-attentiveness to own's body signals may be an important prerequisite for perceptual processes described by Longo et al. (2010; 2016) in their framework model but it is not directly correlated with them. |
| Loret et al. Pilot Study of Functional and Morphometric Brain Abnormalities Related to Adolescent Idiopathic Scoliosis. ClinicalTrials.Gov; Identifier: NCT02302534. | The main aim of this study protocol is to find cortical and subcortical morphometric differences idiopathic scoliosis subjects compared to healthy adolescent control girls: authors did not assessed somatoperception and the proposed investigation is not feasible for clinical practice. |
| Mannerkorpi K, [Arndorw M](https://www.ncbi.nlm.nih.gov/pubmed/?term=Arndorw%20M%5BAuthor%5D&cauthor=true&cauthor_uid=15841606). (2004). Efficacy and feasibility of a combination of body awareness therapy and Qigong in patients with fibromyalgia: a pilot study. J Rehabil Med. 36, 279–281. | The study investigated the use of the Body Awareness Therapy, comprising various breathing, relaxation, concentration and postural techniques and qigong. The concept of Body Awareness is generic and the scale used as outcome measure (Body Awareness Rating Scale) evaluated movements' harmony: out of the scope of this review. |
| Mehling [WE](https://www.ncbi.nlm.nih.gov/pubmed/?term=Mehling%20WE%5BAuthor%5D&cauthor=true&cauthor_uid=23766657), [Daubenmier J](https://www.ncbi.nlm.nih.gov/pubmed/?term=Daubenmier%20J%5BAuthor%5D&cauthor=true&cauthor_uid=23766657), [Price CJ](https://www.ncbi.nlm.nih.gov/pubmed/?term=Price%20CJ%5BAuthor%5D&cauthor=true&cauthor_uid=23766657), [Acree M](https://www.ncbi.nlm.nih.gov/pubmed/?term=Acree%20M%5BAuthor%5D&cauthor=true&cauthor_uid=23766657), [Bartmess E](https://www.ncbi.nlm.nih.gov/pubmed/?term=Bartmess%20E%5BAuthor%5D&cauthor=true&cauthor_uid=23766657), [Stewart AL](https://www.ncbi.nlm.nih.gov/pubmed/?term=Stewart%20AL%5BAuthor%5D&cauthor=true&cauthor_uid=23766657). (2013). Self-reported interoceptive awareness in primary care patients with past or current low back pain. J Pain Res. May, 28, 6, 403-18. doi: 10.2147/JPR.S42418 | Study assessing Interoception through the Multidimensional Assessment of Interoceptive Awareness (MAIA). Interoceptive awareness has been defined as the sense of the physiological condition of the body and sometimes this term is used as synonym of ' body awareness'. Out of the scope of this review. |
| Mehling WE. (2006). Breath therapy for chronic low back pain. Journal of Bodywork and Movement Therapies · April. Vol 10, 2, 96-98. | Insufficient data: authors used a generic definition for body awareness (e.g. differentiating perceptions of physical sensations previously ignored by the patient or overwhelmed by nociceptive input) outcome measure. |
| Mehling WE, [Hamel KA](https://www.ncbi.nlm.nih.gov/pubmed/?term=Hamel%20KA%5BAuthor%5D&cauthor=true&cauthor_uid=16053121), [Acree M](https://www.ncbi.nlm.nih.gov/pubmed/?term=Acree%20M%5BAuthor%5D&cauthor=true&cauthor_uid=16053121), [Byl N](https://www.ncbi.nlm.nih.gov/pubmed/?term=Byl%20N%5BAuthor%5D&cauthor=true&cauthor_uid=16053121), [Hecht FM](https://www.ncbi.nlm.nih.gov/pubmed/?term=Hecht%20FM%5BAuthor%5D&cauthor=true&cauthor_uid=16053121). (2005). Randomized, controlled trial of breath therapy for patients with chronic low-back pain. Alternative Therapies in Health and Medicine. Jul/Aug, 11, 4. | Authors investigate the role of the Breath therapy as a combination of breathing techniques, meditation and movements. Authors declare that it differs from physical therapy in that it minimizes biomechanical and neuromotor control issues and adds a mind-body element of awareness training by focussing the patients' interoceptive attention on their physical sensations: out of the scope of this review. |
| Olsen [AL](https://www.ncbi.nlm.nih.gov/pubmed/?term=Olsen%20AL%5BAuthor%5D&cauthor=true&cauthor_uid=27494177), [Strand LI](https://www.ncbi.nlm.nih.gov/pubmed/?term=Strand%20LI%5BAuthor%5D&cauthor=true&cauthor_uid=27494177), [Skjaerven LH](https://www.ncbi.nlm.nih.gov/pubmed/?term=Skjaerven%20LH%5BAuthor%5D&cauthor=true&cauthor_uid=27494177), [Sundal MA](https://www.ncbi.nlm.nih.gov/pubmed/?term=Sundal%20MA%5BAuthor%5D&cauthor=true&cauthor_uid=27494177), [Magnussen LH](https://www.ncbi.nlm.nih.gov/pubmed/?term=Magnussen%20LH%5BAuthor%5D&cauthor=true&cauthor_uid=27494177). (2017). Patient education and basic body awareness therapy in hip osteoarthritis – a qualitative study of patients’ movement learning experiences. Disability and Rehabilitation. 39, 16, 1631-1638. doi: 10.1080/09638288.2016.1209578 | The study investigate the Body Awareness therapy, involving relaxation, balance and breathing techniques and balance: out of the scope of this review. |
| Malmgren-Olsson et al. (2001). A comparative outcome study of body awareness therapy, Feldenkrais, and conventional physiotherapy for patients with nonspecific musculoskeletal disorders: changes in psychological symptoms, pain, and self-image. Physiotherapy Theory and Practice. 17, 77 –95. | The study investigate the Body Awareness therapy and evaluated changes in self-image under a psychological construct: the scale used as outcome measures (SASB-Structural Analysis of Social Behaviour) assessed affiliation (love-hate) and interdependence (spontaneity-control): out of the scope of this review. |
| Osborn M, Smith JA. (2006). Living with a body separate from the self. The experience of the body in chronic benign low back pain: an interpretative phenomenological analysis. Scand J Caring Sci. 20, 216–222. doi:[10.1111/j.1471-6712.2006.00399.x](https://doi.org/10.1111/j.1471-6712.2006.00399.x) | Questions included in the interview schedule were not focussed specifically on body experience; rather on the self in relation to the body. Participants were asked about how chronic pain and illness experience had changed or influenced their feelings, attitudes and beliefs about themselves. Despite from the study emerge some aspects relative to the theme of the ‘disembodiment’, the introspective approach utilized does not allows to identify if this sense of separation and alienation is related more with the explicit perception of own’s body, or rather with the sense of self-identity integrity and acceptance with respect to an ‘ideal’ self. In this study the 'sense of self' and the 'sense of identity' were defined as the collection of core beliefs, constructs, affects or cognitions utilized to define themselves both privately and with respect to the external world. Thus, all this themes seems to refer more to the concept of the cognitive *somatorepresentation* than of the perceptual *somatorepresentation,* and for this reason, this study does not fit with the scope of this review. |
| Paolucci T, [Zangrando](https://www.hindawi.com/74527382/) F, [Piccinini](https://www.hindawi.com/67543259/) G,[Sciarra](https://www.hindawi.com/10238367/) F, [Pallotta](https://www.hindawi.com/26759317/) R,[Mannocci](https://www.hindawi.com/29378219/) A, [la Torre](https://www.hindawi.com/80763936/) G, [Bini](https://www.hindawi.com/79807389/) F, [Marinozzi](https://www.hindawi.com/26381793/) F,[Gumina](https://www.hindawi.com/63134164/) S, Padua L, Saraceni VM. (2016). New Neurocognitive Interpretation of Shoulder Position Sense during Reaching: Unexpected Competence in the Measurement of Extracorporeal Space. Biomed Res Int. 2016:9065495. | The study assessed repositioning accuracy: out of the topic of this review. |
| Paolucci [T](https://www.ncbi.nlm.nih.gov/pubmed/?term=Paolucci%20T%5BAuthor%5D&cauthor=true&cauthor_uid=27215948), [Zangrando F](https://www.ncbi.nlm.nih.gov/pubmed/?term=Zangrando%20F%5BAuthor%5D&cauthor=true&cauthor_uid=27215948), [Iosa M](https://www.ncbi.nlm.nih.gov/pubmed/?term=Iosa%20M%5BAuthor%5D&cauthor=true&cauthor_uid=27215948), [De Angelis S](https://www.ncbi.nlm.nih.gov/pubmed/?term=De%20Angelis%20S%5BAuthor%5D&cauthor=true&cauthor_uid=27215948), [Marzoli C](https://www.ncbi.nlm.nih.gov/pubmed/?term=Marzoli%20C%5BAuthor%5D&cauthor=true&cauthor_uid=27215948), [Piccinini G](https://www.ncbi.nlm.nih.gov/pubmed/?term=Piccinini%20G%5BAuthor%5D&cauthor=true&cauthor_uid=27215948), [Saraceni VM](https://www.ncbi.nlm.nih.gov/pubmed/?term=Saraceni%20VM%5BAuthor%5D&cauthor=true&cauthor_uid=27215948). (2017). Improved interoceptive awareness in chronic low back pain: a comparison of Back school versus Feldenkrais method. Disability and Rehabilitation. 39, 10, 994-100. doi: 10.1080/09638288.2016.1175035 | The study investigated interoceptive awareness as the attention toward bodily sensations. The Feldenkrais method is a mind–body therapy that is based on the generic ‘awareness’ through movement lessons: out of the scope of this review. |
| Pedler [A](https://www.ncbi.nlm.nih.gov/pubmed/?term=Pedler%20A%5BAuthor%5D&cauthor=true&cauthor_uid=22959229), [Motlagh H](https://www.ncbi.nlm.nih.gov/pubmed/?term=Motlagh%20H%5BAuthor%5D&cauthor=true&cauthor_uid=22959229), [Sterling M](https://www.ncbi.nlm.nih.gov/pubmed/?term=Sterling%20M%5BAuthor%5D&cauthor=true&cauthor_uid=22959229). (2013). Laterality judgments are not impaired in patients with chronic whiplash associated disorders. Man Ther. Feb, 18, (1), 72-6. doi: 10.1016/j.math.2012.07.006 | The study investigated the laterality recognition: out of the scope of this review. |
| Plach SK, Stevens PE, Moss VA. (2004). Corporeality Women’s Experiences of a Body With Rheumatoid Arthritis. Clin Nurs Res. May, 13, (2), 137-155. doi:10.1177/1054773803262219 | The study investigated the impact of illness on daily living: out of the scope of this review. |
| Roosink [M](https://www.ncbi.nlm.nih.gov/pubmed/?term=Roosink%20M%5BAuthor%5D&cauthor=true&cauthor_uid=25799009), [McFadyen BJ](https://www.ncbi.nlm.nih.gov/pubmed/?term=McFadyen%20BJ%5BAuthor%5D&cauthor=true&cauthor_uid=25799009), [Hébert LJ](https://www.ncbi.nlm.nih.gov/pubmed/?term=H%C3%A9bert%20LJ%5BAuthor%5D&cauthor=true&cauthor_uid=25799009), [Jackson PL](https://www.ncbi.nlm.nih.gov/pubmed/?term=Jackson%20PL%5BAuthor%5D&cauthor=true&cauthor_uid=25799009), [Bouyer LJ](https://www.ncbi.nlm.nih.gov/pubmed/?term=Bouyer%20LJ%5BAuthor%5D&cauthor=true&cauthor_uid=25799009), [Mercier C](https://www.ncbi.nlm.nih.gov/pubmed/?term=Mercier%20C%5BAuthor%5D&cauthor=true&cauthor_uid=25799009). (2015). Assessing the Perception of Trunk Movements in Military Personnel with Chronic Non-Specific Low Back Pain Using a Virtual Mirror. PLoS One. Mar, 23, 10, (3), e012025. doi: 10.1371/journal.pone.0120251 | The study investigated the perception of movement: out of inclusion criteria. |
| Seferiadis [A](https://www.ncbi.nlm.nih.gov/pubmed/?term=Seferiadis%20A%5BAuthor%5D&cauthor=true&cauthor_uid=25955823), [Ohlin P](https://www.ncbi.nlm.nih.gov/pubmed/?term=Ohlin%20P%5BAuthor%5D&cauthor=true&cauthor_uid=25955823), [Billhult A](https://www.ncbi.nlm.nih.gov/pubmed/?term=Billhult%20A%5BAuthor%5D&cauthor=true&cauthor_uid=25955823) [Gunnarsson R](https://www.ncbi.nlm.nih.gov/pubmed/?term=Gunnarsson%20R%5BAuthor%5D&cauthor=true&cauthor_uid=25955823). (2016). Basic body awareness therapy or exercise therapy for the treatment of chronic whiplash associated disorders: a randomized comparative clinical trial. Disabil Rehabil, 38, (5), 442–451. doi: 10.3109/09638288.2015.1044036. | The study investigated the effectiveness body awareness therapy (BAT), described as a method that produce a generic improvement of awareness and control of posture: out of the scope of this review. Moreover the study does not provide any outcome measure to assess somatoperception. |
| Sertel [M](https://www.ncbi.nlm.nih.gov/pubmed/?term=Sertel%20M%5BAuthor%5D&cauthor=true&cauthor_uid=28573246), [Bakar Y](https://www.ncbi.nlm.nih.gov/pubmed/?term=Bakar%20Y%5BAuthor%5D&cauthor=true&cauthor_uid=28573246), [Şimşek TT](https://www.ncbi.nlm.nih.gov/pubmed/?term=%C5%9Eim%C5%9Fek%20TT%5BAuthor%5D&cauthor=true&cauthor_uid=28573246). (2017). The effect of body awareness therapy and aerobic exercises on pain and quality of life in the patients with TTH. Afr J Tradit Complement Altern Med. 14 (2): 288-310. doi: 10.21010/ajtcam.v14i2.31 | The study investigated the effectiveness body awareness therapy (BAT). BAT contributes to the physical, mental and spiritual development of the individual and increases the individual’s awareness of these three dimensions. Also, it provides regain and enhancement of posture, balance and natural reflexes of motion to the body: out of the scope of this review. |
| Sherman K, Wellman R, Cook AJ, Cherkin DC, Ceballos RM. (2013). Mediators of Yoga and Stretching for Chronic Low Back Pain. Evidence-Based Complementary and Alternative Medicine, Volume 2013, Article ID 130818, 11 pages. doi: 10.1155/2013/130818 | This study used the Body Responsiveness Questionnaire and the Body Awareness Questionnaire. The former was a 7-item scale designed to measure responsiveness to bodily sensations, while the latter measured self-reported attentiveness to normal non-emotive bodily processes, including sensitivity to body cycles and rhythms, ability to detect small changes in normal function, and ability to anticipate bodily reactions: out of the scope of this review. |
| Sobie TJ. (2016). Body schema acuity training and feldenkrais® movements compared to core stabilization biofeedback and motor control exercises: comparative effects on chronic non-specific low back pain in an outpatient clinical setting: a randomized controlled comparative efficacy study. ProQuest. 449, 10251703. | This PhD thesis studied the ‘body schema acuity training’ through implementation of somatic education and movement therapy interventions (specifically, The Feldenkrais Method®) in conjunction with virtual reality based sensory-motor perceptual experiments: out of the scope of this review. |
| Sobie TJ. (2013). Body Schema and Anatomical Re-framing in the Feldenkrais Method for Low Back Pain. Appl Psychophysiol Biofeedback. 38, 213–238. | Study assessing Feldenkrais-based physical therapy intervention: out of the scope of this review. Authors referred to "proprioceptive touch" but there are insufficient information to judge if this intervention acts on elements of somatoperception as we refer for the scope of this review. |
| Stanton [TR](https://www.ncbi.nlm.nih.gov/pubmed/?term=Stanton%20TR%5BAuthor%5D&cauthor=true&cauthor_uid=28851924), [Moseley GL](https://www.ncbi.nlm.nih.gov/pubmed/?term=Moseley%20GL%5BAuthor%5D&cauthor=true&cauthor_uid=28851924), [Wong AYL](https://www.ncbi.nlm.nih.gov/pubmed/?term=Wong%20AYL%5BAuthor%5D&cauthor=true&cauthor_uid=28851924), [Kawchuk GN](https://www.ncbi.nlm.nih.gov/pubmed/?term=Kawchuk%20GN%5BAuthor%5D&cauthor=true&cauthor_uid=28851924). (2017). Feeling stiffness in the back: a protective perceptual inference in chronic back pain. Sci Rep. Aug 29, 7, (1), 9681. doi: 10.1038/s41598-017-09429-1 | This experimental, laboratory-based study, investigate the disparity between the perceptual phenomenon of feeling a stiffness in low back versus the mechanical response of the spine. Force perception is not considered by Longo et al. (2010; 2016) model: out of the scope of this review. |
| Strand LI, Olsen AL, Nygard H, Furnes O, Magnussen LH, Lygren H, Sundal MA, Skjaerven LH. (2016). Basic Body Awareness Therapy and patient education in hip osteoarthritis: a multiple case study. European Journal of Physiotherapy. 18, (2), 116–125. | This study used the Basic Body Awareness (BAT) as a composite intervention of education and exercise finalised to improve the movement awareness: out of the scope of this review. |
| Strand et al. (2016). Patient Education and Basic Body Awareness Therapy in Hip Osteoarthritis: a Randomized Controlled Trial. ClinicalTrials.gov, Identifier: NCT02884531. | This study used the Body Awareness Rating Scale, assessing the quality of movements: out of the scope of this review. |
| Tsay [A](https://www.ncbi.nlm.nih.gov/pubmed/?term=Tsay%20A%5BAuthor%5D&cauthor=true&cauthor_uid=25783221), [Allen TJ](https://www.ncbi.nlm.nih.gov/pubmed/?term=Allen%20TJ%5BAuthor%5D&cauthor=true&cauthor_uid=25783221), [Proske U](https://www.ncbi.nlm.nih.gov/pubmed/?term=Proske%20U%5BAuthor%5D&cauthor=true&cauthor_uid=25783221), [Giummarra MJ](https://www.ncbi.nlm.nih.gov/pubmed/?term=Giummarra%20MJ%5BAuthor%5D&cauthor=true&cauthor_uid=25783221). (2015). Sensing the body in chronic pain: A review of psychophysical studies implicating altered body representation. Neurosci Biobehav Rev. May, 52, 221-232. doi: 10.1016/j.neubiorev.2015.03.004 | This review analysed mainly the processes of somatosensation (see Longo et. al 2010; 2016): reference list was manually scanned for potential additional sources. |
| Van der Maas [LC](https://www.ncbi.nlm.nih.gov/pubmed/?term=Van%20der%20Maas%20LC%5BAuthor%5D&cauthor=true&cauthor_uid=25119509), [Köke A](https://www.ncbi.nlm.nih.gov/pubmed/?term=K%C3%B6ke%20A%5BAuthor%5D&cauthor=true&cauthor_uid=25119509), [Pont M](https://www.ncbi.nlm.nih.gov/pubmed/?term=Pont%20M%5BAuthor%5D&cauthor=true&cauthor_uid=25119509), [Bosscher RJ](https://www.ncbi.nlm.nih.gov/pubmed/?term=Bosscher%20RJ%5BAuthor%5D&cauthor=true&cauthor_uid=25119509), [Twisk JW](https://www.ncbi.nlm.nih.gov/pubmed/?term=Twisk%20JW%5BAuthor%5D&cauthor=true&cauthor_uid=25119509), [Janssen TW](https://www.ncbi.nlm.nih.gov/pubmed/?term=Janssen%20TW%5BAuthor%5D&cauthor=true&cauthor_uid=25119509), [Peters ML](https://www.ncbi.nlm.nih.gov/pubmed/?term=Peters%20ML%5BAuthor%5D&cauthor=true&cauthor_uid=25119509). (2015). Improving the Multidisciplinary Treatment of Chronic Pain by Stimulating Body Awareness A Cluster-randomized Trial. Clin J Pain. Jul, 31, (7), 660-9. doi: 10.1097/AJP.0000000000000138 | This study evaluated short-term and long-term effects of a multidisciplinary pain rehabilitation program with and without psychomotor therapy (PMT), which focused on body awareness (as a mind-body connection): out of the scope of this review. |
| Zangrado [F](https://www.ncbi.nlm.nih.gov/pubmed/?term=Zangrando%20F%5BAuthor%5D&cauthor=true&cauthor_uid=24622048), [Paolucci T](https://www.ncbi.nlm.nih.gov/pubmed/?term=Paolucci%20T%5BAuthor%5D&cauthor=true&cauthor_uid=24622048), [Vulpiani MC](https://www.ncbi.nlm.nih.gov/pubmed/?term=Vulpiani%20MC%5BAuthor%5D&cauthor=true&cauthor_uid=24622048), [Lamaro M](https://www.ncbi.nlm.nih.gov/pubmed/?term=Lamaro%20M%5BAuthor%5D&cauthor=true&cauthor_uid=24622048), [Isidori R](https://www.ncbi.nlm.nih.gov/pubmed/?term=Isidori%20R%5BAuthor%5D&cauthor=true&cauthor_uid=24622048), [Saraceni VM](https://www.ncbi.nlm.nih.gov/pubmed/?term=Saraceni%20VM%5BAuthor%5D&cauthor=true&cauthor_uid=24622048). (2014). Chronic pain and motor imagery: a rehabilitative experience in a case report. Eur J Phys Rehabil Med. Feb, 50, (1), 67-72. | This study investigated the motor imagery: out of the scope of this review. |
